# Author Correction: Design of a recombinant asparaginyl ligase for site-specific modification using efficient recognition and nucleophile motifs

**DOI:** 10.1038/s42004-024-01196-1

**Published:** 2024-06-03

**Authors:** Jiabao Tang, Mengling Hao, Junxian Liu, Yaling Chen, Gulimire Wufuer, Jie Zhu, Xuejie Zhang, Tingquan Zheng, Mujin Fang, Shiyin Zhang, Tingdong Li, Shengxiang Ge, Jun Zhang, Ningshao Xia

**Affiliations:** 1 State Key Laboratory of Vaccines for Infectious Diseases, School of Public Health, Xiamen University, 361102 Xiamen, China; 2 National Institute of Diagnostics and Vaccine Development in Infectious Diseases, School of Public Health, Xiamen University, 361102 Xiamen, China; 3 National Innovation Platform for Industry-Education Integration in Vaccine Research, School of Public Health, Xiamen University, 361102 Xiamen, China; 4 NMPA Key Laboratory for Research and Evaluation of Infectious Disease Diagnostic Technology, School of Public Health, Xiamen University, 361102 Xiamen, China; 5 Department of Laboratory Medicine, School of Public Health, Xiamen University, 361102 Xiamen, China; 6 Xiang An Biomedicine Laboratory, 361102 Xiamen, China; 7 Jiangsu Key Laboratory of Advanced Catalytic Materials and Technology, School of Petrochemical Engineering, Changzhou University, 213164 Changzhou, China

**Keywords:** Protein design, Ligases, Biocatalysis

Correction to: *Communications Chemistry* 10.1038/s42004-024-01173-8, published online 18 April 2024

An earlier publication by Chua and colleagues reported a truncated construct of oaAEP1b, termed oaAEP1b-C247A-Δ351, which is similar to that reported in this study. In contrast to the previous study, oaAEP1b-C247A-aa55-351 is expressed in soluble form in *E. coli*, facilitated by an N-terminal fusion of a TrxA tag. This strategy eliminates the requirement for the denaturation and subsequent refolding of inclusion bodies using urea, thereby offering a more straightforward approach and ensuring the retention of protein activity.

While this prior publication was initially omitted from the reference list of this Article, the authors acknowledge that given the overlap between the two studies, citation of this earlier work is appropriate.

Chua N. et al. On the design of a constitutively active peptide asparaginyl ligase for facile protein conjugation. *FEBS Open Bio*, **13**, 1195–1106 (2023).

