# Supplementary material for: Design of a recombinant asparaginyl ligase for site-specific modification using efficient recognition and nucleophile motifs
Source: Commun Chem. 2024 Apr 18;7:87. doi: 10.1038/s42004-024-01173-8 (PMC11026461; doi:10.1038/s42004-024-01173-8)
Supplement: Supplementary file 2 — Supplementary Information [file 42004_2024_1173_MOESM2_ESM.pdf]

Design of a recombinant asparaginyl ligase for site-specific modification using efficient recognition and nucleophile motifs

Jiabao Tang‡[1,2,3,4,5,6], Mengling Hao‡[1,2,3,4,5,6], Junxian Liu[1,2,3,4,5,6], Yaling Chen[1,2,3,4,5,6], Gulimire Wufuer[1,2,3,4,5,6], Jie Zhu[7], Xuejie Zhang[1,2,3,4,5,6], Tingquan Zheng[1,2,3,4,5,6], Mujin Fang[1,2,3,4,5,6], Shiyin Zhang[1,2,3,4,5,6], Tingdong Li✉[1,2,3,4,5,6], Shengxiang Ge✉[1,2,3,4,5,6], Jun Zhang[1,2,3,4,5,6], Ningshao Xia[1,2,3,4,5,6]

[1] State Key Laboratory of Vaccines for Infectious Diseases, School of Public Health, Xiamen University, Xiamen 361102, China
[2] National Institute of Diagnostics and Vaccine Development in Infectious Diseases, School of Public Health, Xiamen University, Xiamen 361102, China
[3] National Innovation Platform for Industry-Education Integration in Vaccine Research, School of Public Health, Xiamen University, Xiamen 361102, China
[4] NMPA Key Laboratory for Research and Evaluation of Infectious Disease Diagnostic Technology, School of Public Health, Xiamen University, Xiamen 361102, China
[5] Department of laboratory medicine, School of Public Health, Xiamen University, Xiamen 361102, China
[6] Xiang An Biomedicine Laboratory, Xiamen 361102, China
[7] Jiangsu Key Laboratory of Advanced Catalytic Materials and Technology, School of Petrochemical Engineering, Changzhou University, Changzhou 213164, China

‡These authors contributed equally: Jiabao Tang, Mengling Hao.

✉email: sxge@xmu.edu.cn; litingdong@xmu.edu.cn

Table of Contents:

1. Supplementary Methods...S2

2. Supplementary Figure 1. Design and purification of protein ligase..........S5

3. Supplementary Figure 2. Hydrolysis and nucleophile for peptide ligation catalyzed by OaAEP1-C247A-aa55-351..S6

4. Supplementary Figure 3. OaAEP1-C247A-aa55-351 mediated highly efficient ligation of long peptide or properly-folded protein..........................S7

5. Supplementary Table 1. The information of peptide used in this study....S8

Supplementary Methods

The nucleotide sequence of truncated ligase is shown below:

aa24-351:

GCGCGTGACGGTGATTATCTGCACCTGCCGAGCGAGGTGTCTCGTTTCT
TTCGTCCGCAGGAGACCAACGACGATCACGGCGAAGACAGCGTGGGTA
CCCGTTGGGCTGTTCTGATAGCTGGTTCTAAAGGTTACGCTAACTACCGT
CATCAGGCGGGTGTATGCCACGCCTACCAGATACTGAAACGTGGTGGTC
TGAAAGACGAAAACATCGTTGTTTTCATGTACGACGACATCGCTTACAAC
GAATCTAACCCGCGTCCGGGTGTTATCATCAACTCTCCGCACGGTTCTGA
CGTTTACGCTGGTGTTCCGAAAGACTACACCGGTGAAGAAGTTAACGCTA
AAAACTTCCTGGCTGCTATCCTGGGTAACAAATCTGCTATCACCGGTGGT
TCTGGTAAAGTTGTTGACTCTGGTCCGAACGACCACATCTTCATCTACTAC
ACCGACCACGGTGCTGCTGGTGTTATCGGTATGCCGTCTAAACCGTACCT
GTACGCTGACGAACTGAACGACGCTCTGAAAAAAAAACACGCTTCTGGTA
CCTACAAATCTCTGGTTTTCTACCTGGAAGCGTGCGAATCTGGTTCTATGT
TCGAAGGTATCCTGCCGGAAGACCTGAACATCTACGCTCTGACCTCTACC
AACACCACCGAATCTTCTTGGGCTTACTACTGCCCGGCTCAGGAAAACCC
GCCGCCGCCGGAATACAACGTTTGCCTGGGTGACCTGTTCTCTGTTGCT
TGGCTGGAAGACTCTGACGTTCAGAACTCTTGGTACGAAACCCTGAACC
AGCAGTACCACCACGTTGACAAACGTATCTCTCACGCTTCTCACGCTACC
CAGTACGGTAACCTGAAACTGGGTGAAGAAGGTCTGTTCGTTTACATGGG
TTCTAACCCGGCGAACGACAACTATACCAGCCTGGATGGTAACGCGCTGA
CCCCGAGCAGCATCGTGGTTAACCAGCGTGACGCGGATTGA

aa55-351:

GGTACCCGTTGGGCTGTTCTGATAGCTGGTTCTAAAGGTTACGCTAACTA
CCGTCATCAGGCGGGTGTATGCCACGCCTACCAGATACTGAAACGTGGT
GGTCTGAAAGACGAAAACATCGTTGTTTTCATGTACGACGACATCGCTTA
CAACGAATCTAACCCGCGTCCGGGTGTTATCATCAACTCTCCGCACGGTT

CTGACGTTTACGCTGGTGTTCCGAAAGACTACACCGGTGAAGAAGTTAAC
GCTAAAAACTTCCTGGCTGCTATCCTGGGTAACAAATCTGCTATCACCGGT
GGTTCTGGTAAAGTTGTTGACTCTGGTCCGAACGACCACATCTTCATCTA
CTACACCGACCACGGTGCTGCTGGTGTTATCGGTATGCCGTCTAAACCGT
ACCTGTACGCTGACGAACTGAACGACGCTCTGAAAAAAAAACACGCTTCT
GGTACCTACAAATCTCTGGTTTTCTACCTGGAAGCGTGCGAATCTGGTTC
TATGTTCGAAGGTATCCTGCCGGAAGACCTGAACATCTACGCTCTGACCT
CTACCAACACCACCGAATCTTCTTGGGCTTACTACTGCCCGGCTCAGGAA
AACCCGCCGCCGCCGGAATACAACGTTTGCCTGGGTGACCTGTTCTCTG
TTGCTTGGCTGGAAGACTCTGACGTTCAGAACTCTTGGTACGAAACCCTG
AACCAGCAGTACCACCACGTTGACAAACGTATCTCTCACGCTTCTCACGC
TACCCAGTACGGTAACCTGAAACTGGGTGAAGAAGGTCTGTTCGTTTACA
TGGGTTCTAACCCGGCGAACGACAACTATACCAGCCTGGATGGTAACGC
GCTGACCCCGAGCAGCATCGTGGTTAACCAGCGTGACGCGGATTGA

aa24-325:

GCGCGTGACGGTGATTATCTGCACCTGCCGAGCGAGGTGTCTCGTTTCT
TTCGTCCGCAGGAGACCAACGACGATCACGGCGAAGACAGCGTGGGTA
CCCGTTGGGCTGTTCTGATAGCTGGTTCTAAAGGTTACGCTAACTACCGT
CATCAGGCGGGTGTATGCCACGCCTACCAGATACTGAAACGTGGTGGTC
TGAAAGACGAAAACATCGTTGTTTTCATGTACGACGACATCGCTTACAAC
GAATCTAACCCGCGTCCGGGTGTTATCATCAACTCTCCGCACGGTTCTGA
CGTTTACGCTGGTGTTCCGAAAGACTACACCGGTGAAGAAGTTAACGCTA
AAAACTTCCTGGCTGCTATCCTGGGTAACAAATCTGCTATCACCGGTGGT
TCTGGTAAAGTTGTTGACTCTGGTCCGAACGACCACATCTTCATCTACTAC
ACCGACCACGGTGCTGCTGGTGTTATCGGTATGCCGTCTAAACCGTACCT
GTACGCTGACGAACTGAACGACGCTCTGAAAAAAAAACACGCTTCTGGTA
CCTACAAATCTCTGGTTTTCTACCTGGAAGCGTGCGAATCTGGTTCTATGT
TCGAAGGTATCCTGCCGGAAGACCTGAACATCTACGCTCTGACCTCTACC
AACACCACCGAATCTTCTTGGGCTTACTACTGCCCGGCTCAGGAAAACCC

GCCGCCGCCGGAATACAACGTTTGCCTGGGTGACCTGTTCTCTGTTGCT
TGGCTGGAAGACTCTGACGTTCAGAACTCTTGGTACGAAACCCTGAACC
AGCAGTACCACCACGTTGACAAACGTATCTCTCACGCTTCTCACGCTACC
CAGTACGGTAACCTGAAACTGGGTGAAGAAGGTCTGTTCGTTTACATGGG
TTCTAACCCGTGA

aa55-325:

GGTACCCGTTGGGCTGTTCTGATAGCTGGTTCTAAAGGTTACGCTAACTA
CCGTCATCAGGCGGGTGTATGCCACGCCTACCAGATACTGAAACGTGGT
GGTCTGAAAGACGAAAACATCGTTGTTTTCATGTACGACGACATCGCTTA
CAACGAATCTAACCCGCGTCCGGGTGTTATCATCAACTCTCCGCACGGTT
CTGACGTTTACGCTGGTGTTCCGAAAGACTACACCGGTGAAGAAGTTAAC
GCTAAAAACTTCCTGGCTGCTATCCTGGGTAACAAATCTGCTATCACCGGT
GGTTCTGGTAAAGTTGTTGACTCTGGTCCGAACGACCACATCTTCATCTA
CTACACCGACCACGGTGCTGCTGGTGTTATCGGTATGCCGTCTAAACCGT
ACCTGTACGCTGACGAACTGAACGACGCTCTGAAAAAAAAACACGCTTCT
GGTACCTACAAATCTCTGGTTTTCTACCTGGAAGCGTGCGAATCTGGTTC
TATGTTCGAAGGTATCCTGCCGGAAGACCTGAACATCTACGCTCTGACCT
CTACCAACACCACCGAATCTTCTTGGGCTTACTACTGCCCGGCTCAGGAA
AACCCGCCGCCGCCGGAATACAACGTTTGCCTGGGTGACCTGTTCTCTG
TTGCTTGGCTGGAAGACTCTGACGTTCAGAACTCTTGGTACGAAACCCTG
AACCAGCAGTACCACCACGTTGACAAACGTATCTCTCACGCTTCTCACGC
TACCCAGTACGGTAACCTGAAACTGGGTGAAGAAGGTCTGTTCGTTTACA
TGGGTTCTAACCCGTGA

Supplementary Figures

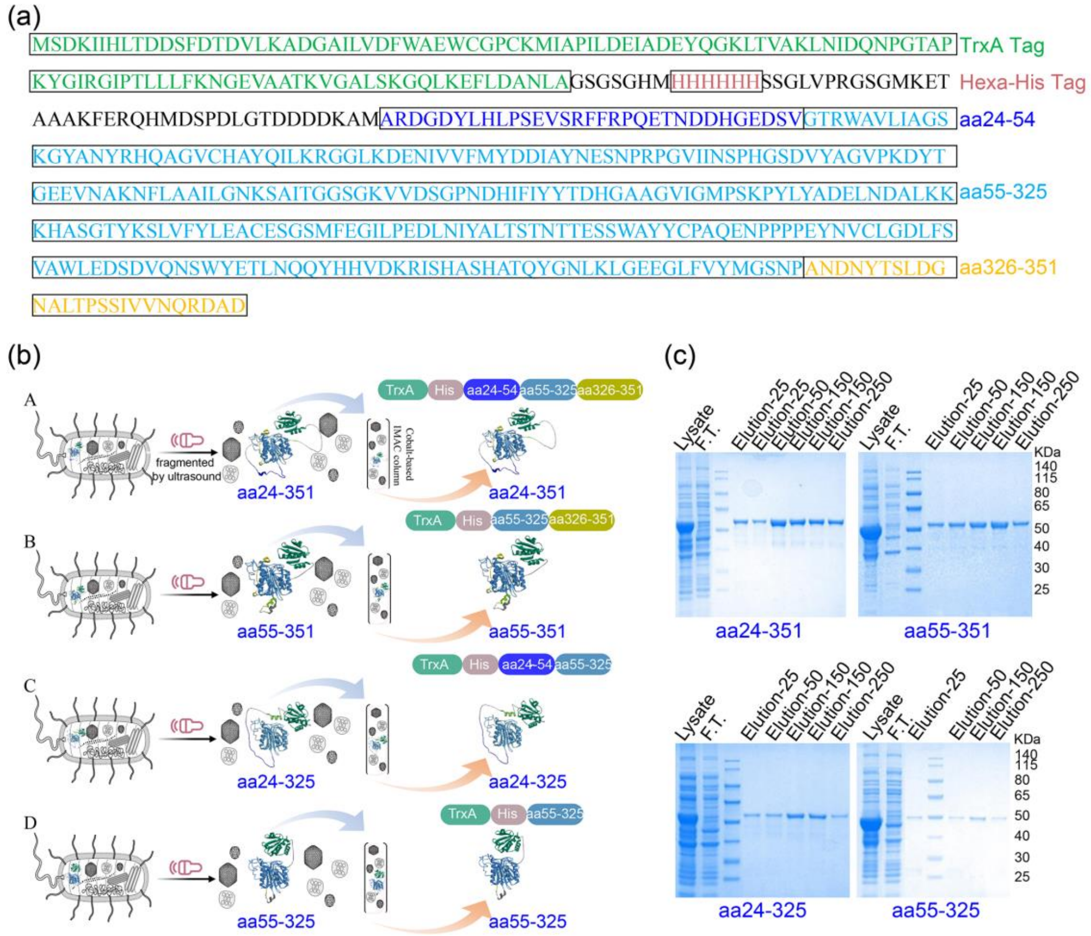

Supplementary Figure 1. Design and purification of protein ligase. (a) Amino acid sequence of the protein OaAEP1-C247A-aa24-351 expressed in this study. OaAEP1-C247A-aa55-351, OaAEP1-C247A-aa24-325 and OaAEP1-C247A-aa55-325 were removed the aa24-54, aa326-351 and both, respectively. (b) Schematic diagram of the four truncated ligases express and purified processes. The structures of OaAEP1-C247A truncations were predicted by AlphaFold2. (c) SDS-PAGE analysis of fractions after purification of the OaAEP1-C247A truncations by cobalt-based IMAC column. Elution-No. was represented fractions eluted with 25, 50, 150 and 250 mM imidazole, respectively.

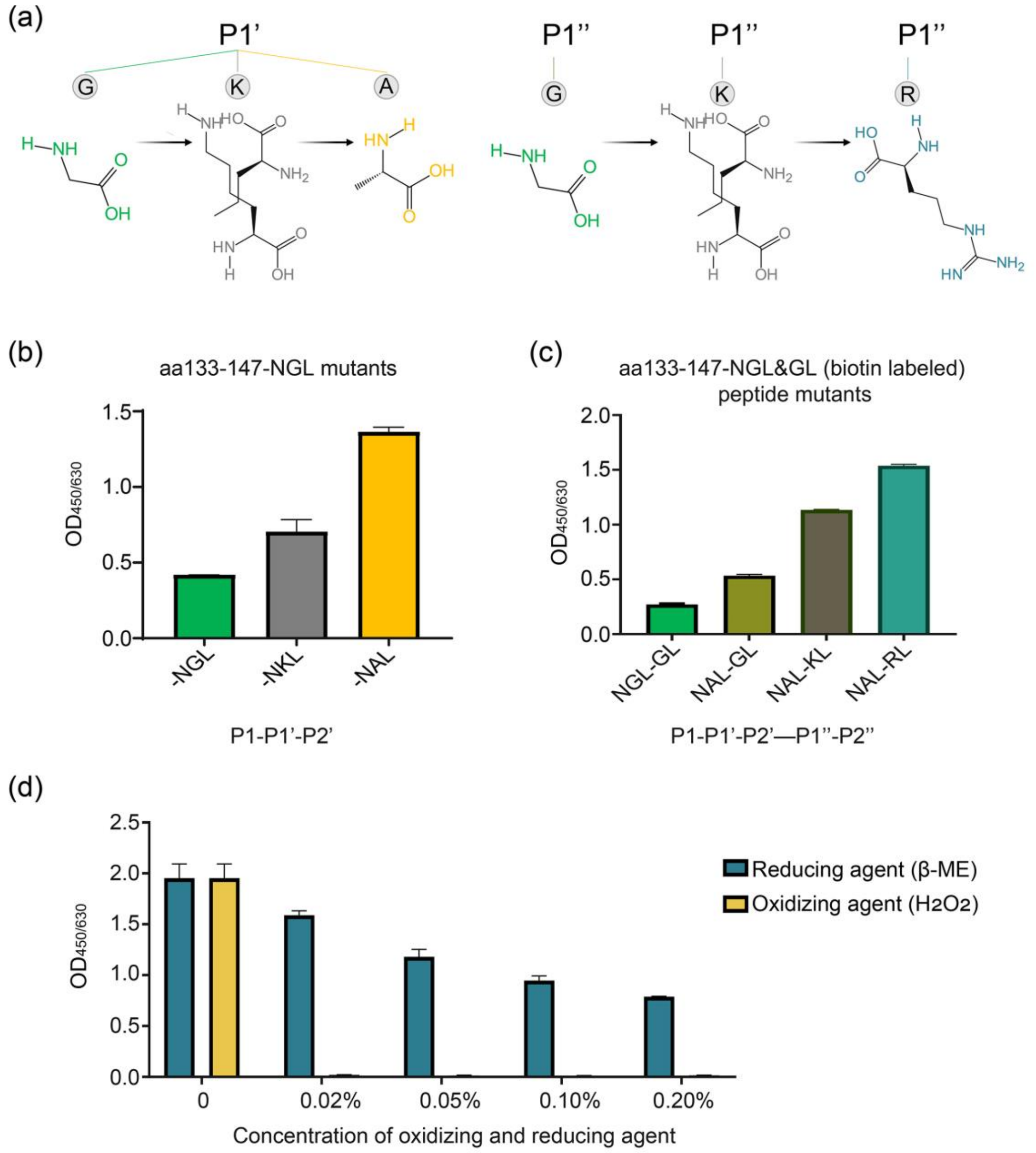

Supplementary Figure 2. Hydrolysis and nucleophile for peptide ligation catalyzed by OaAEP1-C247A-aa55-351. (a) Representative amino acid structures of P1' and P1''. (b) Comparison of the capacity of OaAEP1-C247A-aa55-351 to ligate "Asn-Gly-Leu", "Asn-Lys-Leu" and "Asn-Ala-Leu" to "Gly-Leu"-based peptide. To further compare the ligation efficiency, the ligation products were detected after further dilution at a higher-fold (100-fold dilution). (c) Peptide ligation efficiency of multiple recognition motif and nucleophile sequence mutants. (d) The effect of oxi-reductive addictive in promoting the ligating efficiency of OaAEP1-C247A-aa55-351. 2 μM Pep133-NAL and 10 μM RL-(biotin labeled) peptide were ligated by 0.2 μM OaAEP1-C247A-aa55-351 with different concentrations of β-mercaptoethanol (β-ME, reducing agent) or H_2O_2 (Oxidizing agent) at 37℃ for 30 min.

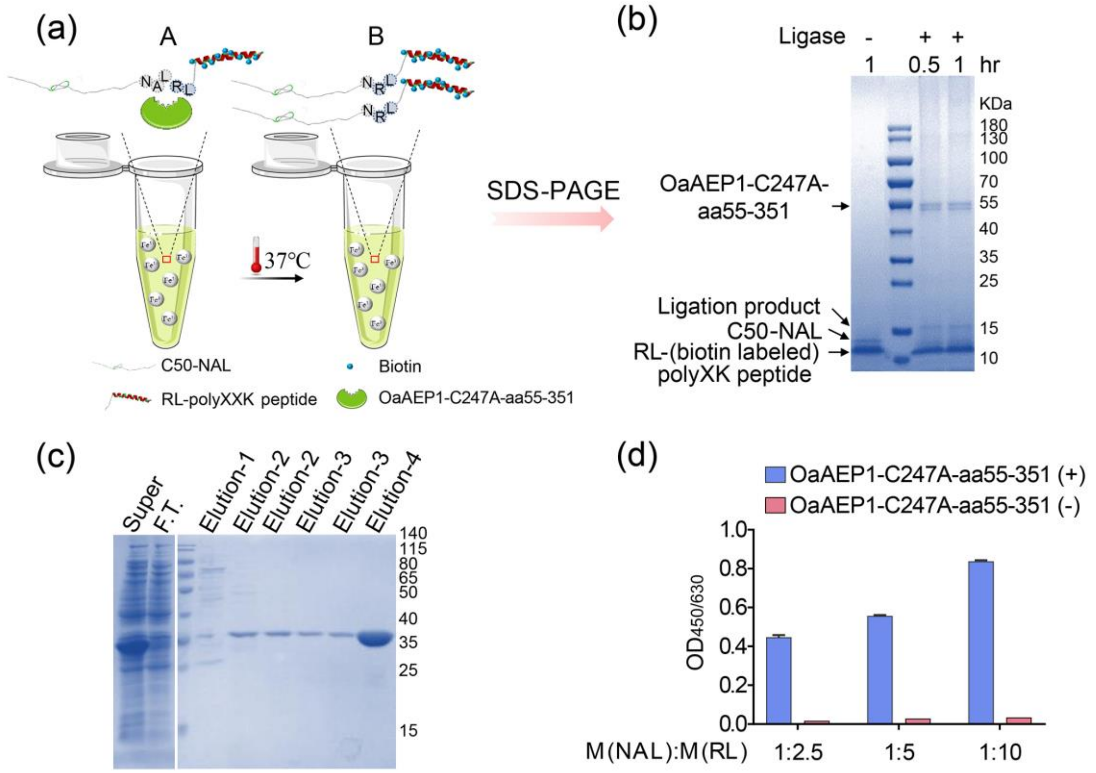

Supplementary Figure 3. OaAEP1-C247A-aa55-351 mediated highly efficient ligation of long peptide or properly-folded protein. (a) Schematic diagram of evaluating the product yield of longer peptides C50-NAL and RL-(biotin labeled) polyXXK peptide ligating by OaAEP1-C247A-aa55-351 with Fe^{3+}. (b) SDS-PAGE analysis of the yield of product after ligating by OaAEP1-C247A-aa55-351. 5 μM C50-NAL and 50 μM RL- (biotin labeled) polyXXK peptide were ligated by 1 μM OaAEP1-C247A-aa55-351 and 1mM Fe^{3+} in phosphate buffer, pH 7.2 at 37℃ for 30 min and 1 hour, respectively. The ligating product were subjected to analyzed by SDS-PAGE. The bands of OaAEP1-C247A-aa55-351, ligation product, C50-NAL and RL- (biotin labeled) polyXXK peptide were annotated in the figure. (c) SDS-PAGE analysis of fractions after purification of the rtNP-NAL by HiTrap SP HP cation exchange chromatography column. Super.: supernatant of bacteria lysates after sonication and centrifugation; F.T.: The flow-through fractions of column purification. Elution-1~4 represent the elution fractions of 100, 200, 300, 400 mM NaCl. (d) The ligation activity of OaAEP1-C247A-aa55-351 in ligating properly-folded protein substrates. 2 μM rtNP-NAL and 5 μM, 10 μM, 20 μM RL-(biotin labeled) peptide were ligated by 0.5 μM OaAEP1-C247A-aa55-351 at 37℃for 30 min, respectively. The product was detected by ELISA with rtNP antibody, 17H11.

Supplementary Tables

Supplementary Table 1. The information of peptide used in this study

Peptide name	Peptide sequence	Expected mass (m/z) [M]+	Observed mass (m/z) [M+H] +
Recognition peptides			
Pep133-NGL	SAAERKHRHLPVADANGL	1942.153	1942.050
Pep133-AGL	SAAERKHRHLPVADAAGL	1899.128	1899.300
Pep133-QGL	SAAERKHRHLPVADAQGL	1956.180	1956.000
Pep133-FGL	SAAERKHRHLPVADAFGL	1975.226	1975.200
Pep133-DGL	SAAERKHRHLPVADADGL	1943.130	1943.100
Pep133-KGL	SAAERKHRHLPVADAKGL	1956.219	1956.400
Pep133-NAL	SAAERKHRHLPVADANAL	1956.180	1956.150
Pep133-NQL	SAAERKHRHLPVADANQL	2013.232	2013.232
Pep133-NFL	SAAERKHRHLPVADANFL	2032.278	2032.200
Pep133-NDL	SAAERKHRHLPVADANDL	2000.190	2000.600
Pep133-NKL	SAAERKHRHLPVADANKL	2013.271	2013.400
Pep133-NGA	SAAERKHRHLPVADANGA	1900.072	1900.200
Pep133-NGQ	SAAERKHRHLPVADANGQ	1957.124	1957.050
Pep133-NGF	SAAERKHRHLPVADANGF	1976.170	1976.100
Pep133-NGD	SAAERKHRHLPVADANGD	1944.082	1944.000
Pep133-NGK	SAAERKHRHLPVADANGK	1957.163	1957.200
Pep133-NVL	SAAERKHRHLPVADANVL	1984.234	1984.400
Pep133-NLL	SAAERKHRHLPVADANLL	1998.261	1998.450
Pep133-NPL	SAAERKHRHLPVADANPL	1982.218	1982.250
Pep133-NAL (FAM labeled)	SAAERKHRHLPVADA KNAL	2442.650	2442.600
C50-NAL	PQRKTKRNTNRRPQDVKFPG GGQIVGGVYLLPRRGPRLGVR ATRKTSERSNAL	5941.838	5943.60

continued			
Nucleophilic peptides			
GL-(biotin labeled) peptide	GLPVK(biotin labeled)AR	966.220	965.600
AL-(biotin-labeled) peptide	ALPVK(biotin labeled)AR	980.236	980.300
QL-(biotin-labeled) peptide	QLPVK(biotin labeled)AR	1037.288	1037.300
FL-(biotin-labeled) peptide	FLPVK(biotin labeled)AR	1056.334	1056.200
DL-(biotin-labeled) peptide	DLPVK(biotin labeled)AR	1024.246	1024.300
KL-(biotin-labeled) peptide	KLPVK(biotin labeled)AR	1037.327	1037.300
GA-(biotin-labeled) peptide	GAPVK(biotin labeled)AR	924.128	924.100
GQ-(biotin-labeled) peptide	GQPVK(biotin labeled)AR	981.180	981.000
GF-(biotin-labeled) peptide	GFPVK(biotin labeled)AR	1000.226	1000.100
GD-(biotin-labeled) peptide	GDPVK(biotin labeled)AR	968.138	968.000
GK-(biotin-labeled) peptide	GKPVK(biotin labeled)AR	981.219	981.200
AGL-(biotin labeled) peptide	AGLPVK(biotin labeled)AR	1037.288	1037.300
QGL-(biotin-labeled) peptide	QGLPVK(biotin labeled)AR	1094.340	1094.200
FGL-(biotin-labeled) peptide	FGLPVK(biotin labeled)AR	1113.386	1113.200
DGL-(biotin-labeled) peptide	DGLPVK(biotin labeled)AR	1081.298	1081.300
KGL-(biotin-labeled) peptide	KGLPVK(biotin labeled)AR	1094.379	1094.300
HL-(biotin labeled) peptide	HLPVK(biotin labeled)AR	1046.298	1046.400
RL-(biotin labeled) peptide	RLPVK(biotin labeled)AR	1065.345	1065.400
RL-(Dabcyl labeled) peptide	RLPVK(Dabcyl labeled)AR	1090.335	1090.05
RL-polyXXK (biotin labeled) peptide	RLGGGGSKENKRHKQRKHEK GLKNRKHEKGLKNEK (All K labeled with biotin)	6354.741	6355.400
