# Supplementary material for: Design of a recombinant asparaginyl ligase for site-specific modification using efficient recognition and nucleophile motifs
Source: Commun Chem. 2024 Apr 18;7:87. doi: 10.1038/s42004-024-01173-8 (PMC11026461; doi:10.1038/s42004-024-01173-8)
Supplement: Supplementary file 3 — Description of Additional Supplementary Files [file 42004_2024_1173_MOESM3_ESM.pdf]

Description of Additional Supplementary Files

File name: Supplementary Data 1

Description: HPLC chromatograms and LC/MS spectra of the peptides

File name: Supplementary Data 2

Description: Source data of the figures
