# Supplementary material for: Design of a recombinant asparaginyl ligase for site-specific modification using efficient recognition and nucleophile motifs
Source: Commun Chem. 2024 Apr 18;7:87. doi: 10.1038/s42004-024-01173-8 (PMC11026461; doi:10.1038/s42004-024-01173-8)
Supplement: Supplementary file 4 — Supplementary Data 1 [file 42004_2024_1173_MOESM4_ESM.pdf]

Design of a recombinant asparaginyl ligase for site-specific modification using efficient recognition and nucleophile motifs

Jiabao Tang[‡1,2,3,4,5,6], Mengling Hao[‡1,2,3,4,5,6], Junxian Liu[1,2,3,4,5,6], Yaling Chen[1,2,3,4,5,6], Gulimire Wufuer[1,2,3,4,5,6], Jie Zhu[7], Xuejie Zhang[1,2,3,4,5,6], Tingquan Zheng[1,2,3,4,5,6], Mujin Fang[1,2,3,4,5,6], Shiyin Zhang[1,2,3,4,5,6], Tingdong Li[✉1,2,3,4,5,6], Shengxiang Ge[✉1,2,3,4,5,6], Jun Zhang[1,2,3,4,5,6], Ningshao Xia[1,2,3,4,5,6]

[1] State Key Laboratory of Vaccines for Infectious Diseases, School of Public Health, Xiamen University, Xiamen 361102, China
[2] National Institute of Diagnostics and Vaccine Development in Infectious Diseases, School of Public Health, Xiamen University, Xiamen 361102, China
[3] National Innovation Platform for Industry-Education Integration in Vaccine Research, School of Public Health, Xiamen University, Xiamen 361102, China
[4] NMPA Key Laboratory for Research and Evaluation of Infectious Disease Diagnostic Technology, School of Public Health, Xiamen University, Xiamen 361102, China
[5] Department of laboratory medicine, School of Public Health, Xiamen University, Xiamen 361102, China
[6] Xiang An Biomedicine Laboratory, Xiamen 361102, China
[7] Jiangsu Key Laboratory of Advanced Catalytic Materials and Technology, School of Petrochemical Engineering, Changzhou University, Changzhou 213164, China

[‡]These authors contributed equally: Jiabao Tang, Mengling Hao.
[✉]email: sxge@xmu.edu.cn; litingdong@xmu.edu.cn

HPLC chromatograms and LC/MS spectra of the peptides

Table of contents

HPLC traces of peptides

Pep133-NGL······1
Pep133-AGL······2
Pep133-QGL······3
Pep133-FGL······4
Pep133-DGL······5
Pep133-KGL······6
Pep133-NAL······7
Pep133-NQL······8
Pep133-NFL······9
Pep133-NDL······10
Pep133-NKL······11
Pep133-NGA······12
Pep133-NGQ······13
Pep133-NGF······14
Pep133-NGD······15
Pep133-NGK······16
Pep133-NVL······17
Pep133-NLL······18
Pep133-NPL······19
Pep133-NAL (FAM labeled)······20
C50-NAL······21
GL-(biotin labeled) peptide······22
AL-(biotin labeled) peptide······23
QL-(biotin labeled) peptide······24
FL-(biotin labeled) peptide······25
DL-(biotin labeled) peptide······26
KL-(biotin labeled) peptide······27
GA-(biotin labeled) peptide······28
GQ-(biotin labeled) peptide······29
GF-(biotin labeled) peptide······30
GD-(biotin labeled) peptide······31
GK-(biotin labeled) peptide······32
AGL-(biotin labeled) peptide······33
QGL-(biotin labeled) peptide······34
FGL-(biotin labeled) peptide······35
DGL-(biotin labeled) peptide······36
KGL-(biotin labeled) peptide······37
HL-(biotin labeled) peptide······38
RL-(biotin labeled) peptide······39
RL-(Dabcyl labeled) peptide······40

LC/MS traces of peptides

Pep133-NGL······41
Pep133-AGL······42
Pep133-QGL······43
Pep133-FGL······44
Pep133-DGL······45
Pep133-KGL······46
Pep133-NAL······47
Pep133-NQL······48
Pep133-NFL······49
Pep133-NDL······50
Pep133-NKL······51
Pep133-NGA······52
Pep133-NGQ······53
Pep133-NGF······54
Pep133-NGD······55
Pep133-NGK······56
Pep133-NVL······57
Pep133-NLL······58
Pep133-NPL······59
Pep133-NAL (FAM labeled)······60
C50-NAL······61
GL-(biotin labeled) peptide······62
AL-(biotin labeled) peptide······63
QL-(biotin labeled) peptide······64
FL-(biotin labeled) peptide······65
DL-(biotin labeled) peptide······66
KL-(biotin labeled) peptide······67
GA-(biotin labeled) peptide······68
GQ-(biotin labeled) peptide······69
GF-(biotin labeled) peptide······70
GD-(biotin labeled) peptide······71
GK-(biotin labeled) peptide······72
AGL-(biotin labeled) peptide······73
QGL-(biotin labeled) peptide······74
FGL-(biotin labeled) peptide······75
DGL-(biotin labeled) peptide······76
KGL-(biotin labeled) peptide······77
HL-(biotin labeled) peptide······78
RL-(biotin labeled) peptide······79
RL-(Dabcyl labeled) peptide······80

Sample Information

Name : P22101 (Pep133-NGL)
Sequence : SAAERKHRHLPVADANGL
Modification : N/A
Lot.No : P22101 (Pep133-NGL)
Pump A : 0.1%trifluoroacetic in 100%water
Pump B : 0.1%trifluoroacetic in 100%acetonrtrile
Total Flow : 1.0ml/min
Wavelength : 214nm
Analytical column type : NanoChrom Chromcore TM120 C18(4.6*250MM*5UM)
Dissolution method : 0.5mg sample dissolved to 0.5mL by 100%H2O
Inj.Volume : 60ul

Time	Module	Action	Value
0.01	Pumps	B.Conc	15
20.00	Pumps	B.Conc	35

Chromatogram

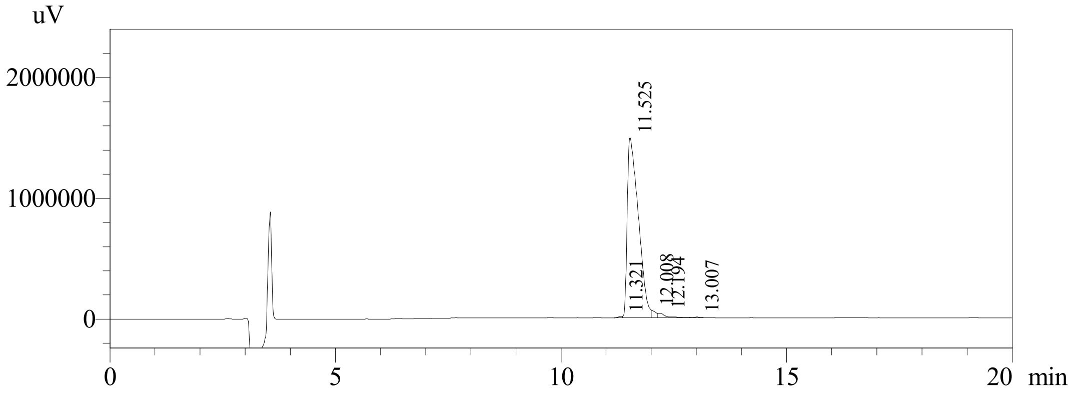

PeakTable

Detector A Ch1 214nm

Peak#	Ret. Time	Area	Height	Area %	Height %
1	11.321	64374	10477	0.243	0.654
2	11.525	25497681	1487300	96.352	92.880
3	12.008	399002	62642	1.508	3.912
4	12.194	462029	35527	1.746	2.219
5	13.007	40094	5366	0.152	0.335
Total		26463180	1601312	100.000	100.000

生工生物工程（上海）股份有限公司

地址: 上海市松江区香闵路698号 Add: 698 Xiang Min Road SongJiang Shanghai China
电话/Tel: 400-821-0268 传真/Fax: 86-21-37772170
邮箱/Email: sales@sangon.com 网址/Web: www.sangon.com

生工® Sangon Biotech

Sample Information

Name	:	P20571-1 (Pep133-AGL)
Sequence	:	SAAERKHRHLPVADAAGL
Modification	:	N/A
Lot.No	:	P20571-1 (Pep133-AGL)
Pump A	:	0.1%trifluoroacetic in 100%water
Pump B	:	0.1%trifluoroacetic in 100%acetonrtrile
Total Flow	:	1.0ml/min
Wavelength	:	214nm
Analytical column type	:	SHIMADZU Inertsil ODS-SP(4.6*150MM*5UM)
Dissolution method	:	0.5mg sample dissolved to 0.5mL by 100%H2O
Inj.Volume	:	60ul

Time	Module	Action	Value
0.01	Pumps	B.Conc	21
20.00	Pumps	B.Conc	41

Chromatogram

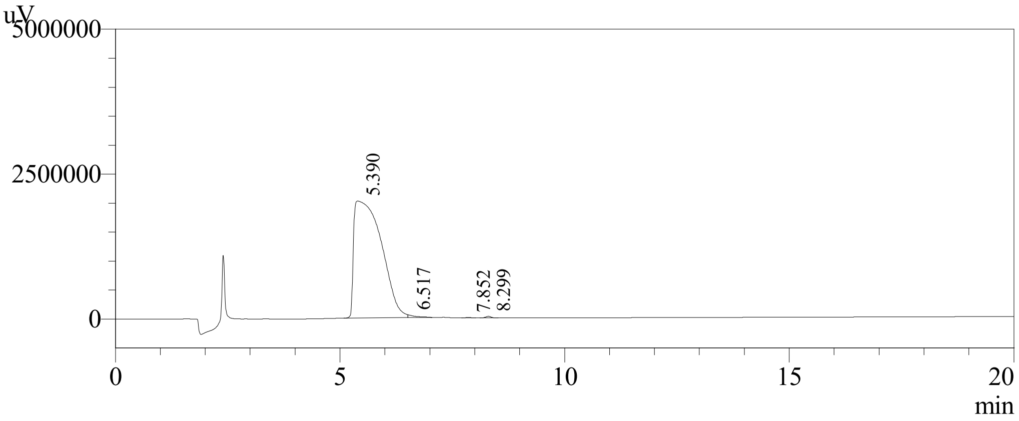

1 Det.A Ch1 / 214nm

PeakTable

Detector A Ch1 214nm

Peak#	Ret. Time	Area	Height	Area %	Height %
1	5.390	86423744	2018426	99.027	96.020
2	6.517	554680	48063	0.636	2.286
3	7.852	62359	7961	0.071	0.379
4	8.299	231919	27629	0.266	1.314
Total		87272702	2102079	100.000	100.000

生工生物工程（上海）股份有限公司

地址: 上海市松江区香闵路698号 Add: 698 Xiang Min Road SongJiang Shanghai China
电话/Tel: 400-821-0268 传真/Fax: 86-21-37772170
邮箱/Email: sales@sangon.com 网址/Web: www.sangon.com

Sample Information

Name	:	P20571-2 (Pep133-QGL)
Sequence	:	SAAERKHRHLPVADAQGL
Modification	:	N/A
Lot.No	:	P20571-2（Pep133-QGL)
Pump A	:	0.1%trifluoroacetic in 100%water
Pump B	:	0.1%trifluoroacetic in 100%acetonrtrile
Total Flow	:	1.0ml/min
Wavelength	:	214nm
Analytical column type	:	SHIMADZU Inertsil ODS-SP(4.6*150MM*5UM)
Dissolution method	:	0.5mg sample dissolved to 0.5mL by 100%H2O
Inj.Volume	:	60ul

Time	Module	Action	Value
0.01	Pumps	B.Conc	17
20.00	Pumps	B.Conc	37

Chromatogram

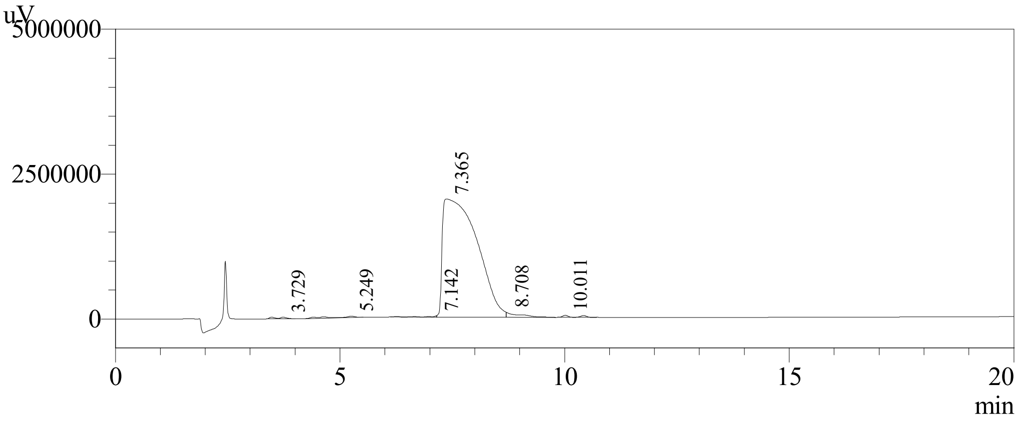

1 Det.A Ch1 / 214nm

PeakTable

Detector A Ch1 214nm

Peak#	Ret. Time	Area	Height	Area %	Height %
1	3.729	529939	28164	0.468	1.262
2	5.249	898094	23140	0.794	1.037
3	7.142	272625	23070	0.241	1.034
4	7.365	109220744	2039244	96.532	91.385
5	8.708	1670280	82050	1.476	3.677
6	10.011	552639	35830	0.488	1.606
Total		113144321	2231497	100.000	100.000

生工生物工程（上海）股份有限公司

地址: 上海市松江区香闵路698号 Add: 698 Xiang Min Road SongJiang Shanghai China
电话/Tel: 400-821-0268 传真/Fax: 86-21-37772170
邮箱/Email: sales@sangon.com 网址/Web: www.sangon.com

生工® Sangon Biotech

Sample Information

Name	:	P20571-3 (Pep133-FGL)
Sequence	:	SAAERKHRHLPVADAFGL
Modification	:	N/A
Lot.No	:	P20571-3 (Pep133-FGL)
Pump A	:	0.1%trifluoroacetic in 100%water
Pump B	:	0.1%trifluoroacetic in 100%acetonrtrile
Total Flow	:	1.0ml/min
Wavelength	:	214nm
Analytical column type	:	SHIMADZU Inertsil ODS-SP(4.6*150MM*5UM)
Dissolution method	:	0.5mg sample dissolved to 0.5mL by 100%H2O
Inj.Volume	:	60ul

Time	Module	Action	Value
0.01	Pumps	B.Conc	17
20.00	Pumps	B.Conc	37

Chromatogram

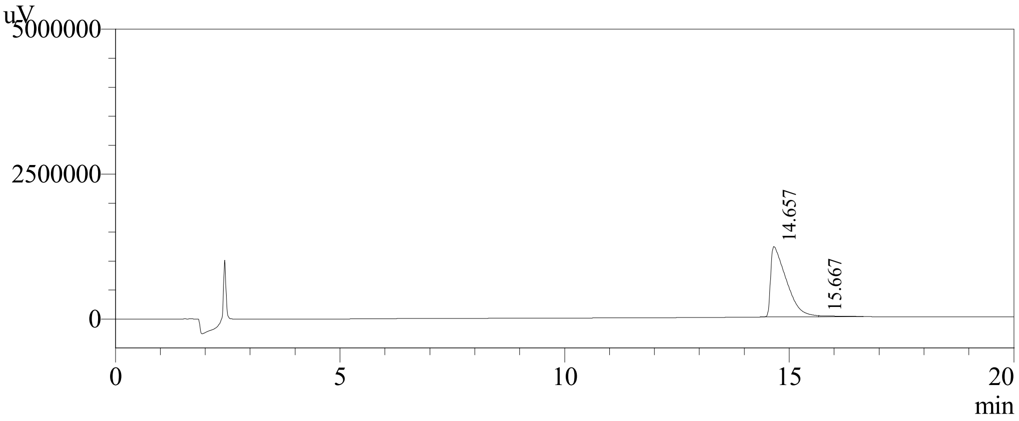

1 Det.A Ch1 / 214nm

PeakTable

Detector A Ch1 214nm

Peak#	Ret. Time	Area	Height	Area %	Height %
1	14.657	29524239	1214208	98.279	98.520
2	15.667	517152	18235	1.721	1.480
Total		30041392	1232443	100.000	100.000

生工生物工程（上海）股份有限公司

地址: 上海市松江区香闵路698号
电话/Tel: 400-821-0268
邮箱/Email: sales@sangon.com

Add: 698 Xiang Min Road SongJiang Shanghai China
传真/Fax: 86-21-37772170
网址/Web: www.sangon.com

Sample Information

Name	:	P20571-4 (Pep133-DGL)
Sequence	:	SAAERKHRHLPVADADGL
Modification	:	N/A
Lot.No	:	P20571-4 (Pep133-DGL)
Pump A	:	0.1%trifluoroacetic in 100%water
Pump B	:	0.1%trifluoroacetic in 100%acetonrtrile
Total Flow	:	1.0ml/min
Wavelength	:	214nm
Analytical column type	:	SHIMADZU Inertsil ODS-SP(4.6*150MM*5UM)
Dissolution method	:	0.5mg sample dissolved to 0.5mL by 100%H2O
Inj.Volume	:	60ul

Time	Module	Action	Value
0.01	Pumps	B.Conc	17
20.00	Pumps	B.Conc	37

Chromatogram

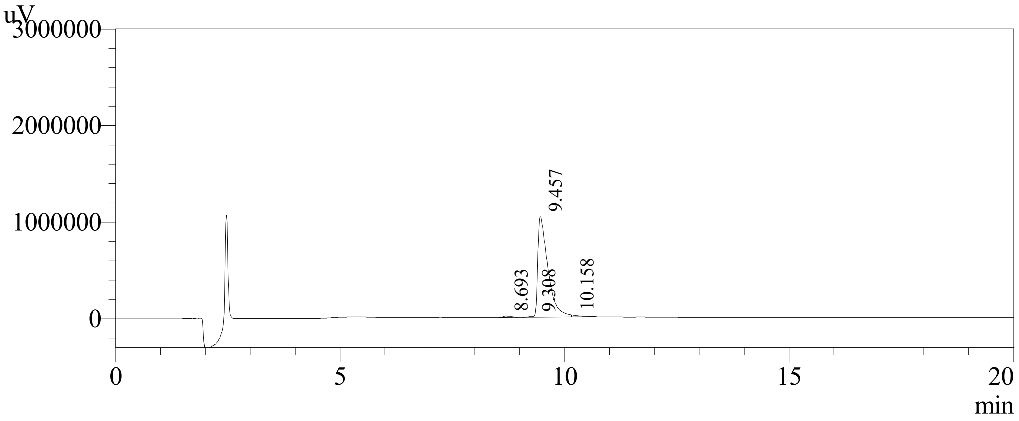

1 Det.A Ch1 / 214nm

PeakTable

Detector A Ch1 214nm

Peak#	Ret. Time	Area	Height	Area %	Height %
1	8.693	188429	14825	1.126	1.367
2	9.308	63973	8424	0.382	0.777
3	9.457	16241049	1041058	97.089	95.983
4	10.158	234631	20318	1.403	1.873
Total		16728082	1084625	100.000	100.000

生工生物工程（上海）股份有限公司

地址: 上海市松江区香闵路698号
电话/Tel: 400-821-0268
邮箱/Email: sales@sangon.com

Add: 698 Xiang Min Road SongJiang Shanghai China
传真/Fax: 86-21-37772170
网址/Web: www.sangon.com

生工® Sangon Biotech

Sample Information

Name : P20571-5 (Pep133-KGL)
Sequence : SAAERKHRHLPVADAKGL
Modification : N/A
Lot.No : P20571-5 (Pep133-KGL)
Pump A : 0.1%trifluoroacetic in 100%water
Pump B : 0.1%trifluoroacetic in 100%acetonrtrile
Total Flow : 1.0ml/min
Wavelength : 214nm
Analytical column type : SHIMADZU Inertsil ODS-SP(4.6*150MM*5UM)
Dissolution method : 0.5mg sample dissolved to 0.5mL by 100%H2O
Inj.Volume : 60ul

Time	Module	Action	Value
0.01	Pumps	B.Conc	15
20.00	Pumps	B.Conc	35

Chromatogram

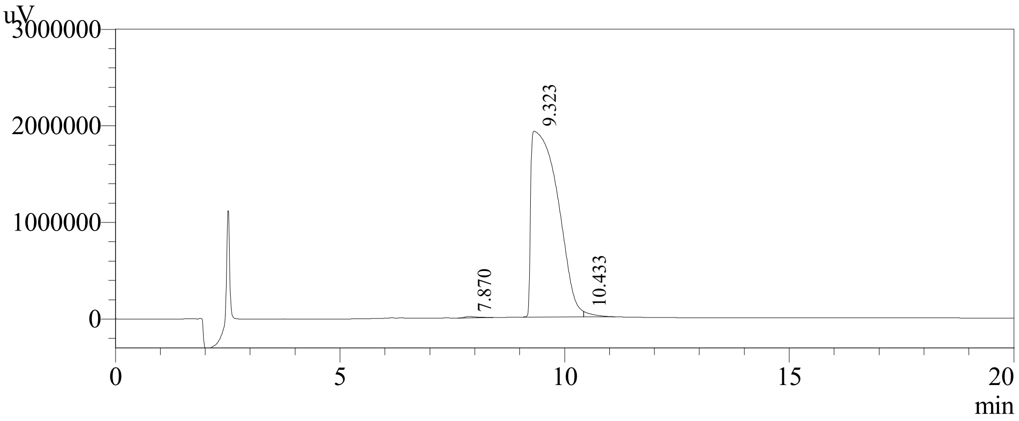

1 Det.A Ch1 / 214nm

PeakTable

Detector A Ch1 214nm

Peak#	Ret. Time	Area	Height	Area %	Height %
1	7.870	264548	13369	0.326	0.671
2	9.323	80194445	1923915	98.805	96.597
3	10.433	705599	54409	0.869	2.732
Total		81164592	1991692	100.000	100.000

生工生物工程（上海）股份有限公司

地址: 上海市松江区香闵路698号
电话/Tel: 400-821-0268
邮箱/Email: sales@sangon.com

Add: 698 Xiang Min Road SongJiang Shanghai China
传真/Fax: 86-21-37772170
网址/Web: www.sangon.com

Sample Information

Name	:	P20571-6 (Pep133-NAL)
Sequence	:	SAAERKHRHLPVADANAL
Modification	:	N/A
Lot.No	:	P20571-6 (Pep133-NAL)
Pump A	:	0.1%trifluoroacetic in 100%water
Pump B	:	0.1%trifluoroacetic in 100%acetonrtrile
Total Flow	:	1.0ml/min
Wavelength	:	214nm
Analytical column type	:	SHIMADZU Inertsil ODS-SP(4.6*250MM*5UM)
Dissolution method	:	0.5mg sample dissolved to 0.5mL by 100%H2O
Inj.Volume	:	60ul

Time	Module	Action	Value
0.01	Pumps	B.Conc	17
20.00	Pumps	B.Conc	37
20.01	Controller	Stop	

Chromatogram

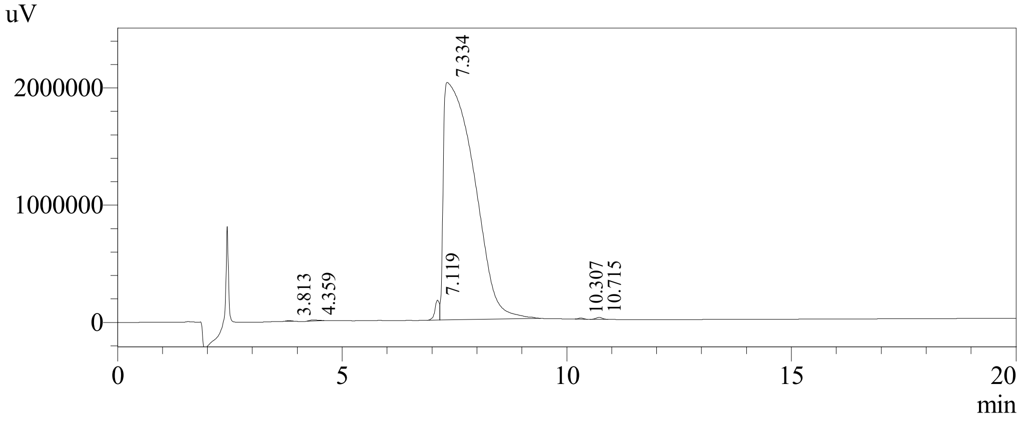

1 Det.A Ch1 / 214nm

PeakTable

Detector A Ch1 214nm

Peak#	Ret. Time	Area	Height	Area %	Height %
1	3.813	51748	6182	0.053	0.276
2	4.359	110312	9276	0.113	0.414
3	7.119	1217367	171479	1.243	7.661
4	7.334	96298305	2026091	98.355	90.514
5	10.307	74080	9567	0.076	0.427
6	10.715	157408	15840	0.161	0.708
Total		97909219	2238436	100.000	100.000

生工生物工程（上海）股份有限公司

地址: 上海市松江区香闵路698号
电话/Tel: 400-821-0268
邮箱/Email: sales@sangon.com
Add: 698 Xiang Min Road SongJiang Shanghai China
传真/Fax: 86-21-37772170
网址/Web: www.sangon.com

生工® Sangon Biotech

Sample Information

Name	:	P20571-7 (Pep133-NQL)
Sequence	:	SAAERKHRHLPVADANQL
Modification	:	N/A
Lot.No	:	P20571-7 (Pep133-NQL)
Pump A	:	0.1%trifluoroacetic in 100%water
Pump B	:	0.1%trifluoroacetic in 100%acetonrtrile
Total Flow	:	1.0ml/min
Wavelength	:	214nm
Analytical column type	:	SHIMADZU Inertsil ODS-SP(4.6*250MM*5UM)
Dissolution method	:	0.5mg sample dissolved to 0.5mL by 100%H2O
Inj.Volume	:	60ul

Time	Module	Action	Value
0.01	Pumps	B.Conc	16
20.00	Pumps	B.Conc	36
20.01	Controller	Stop	

Chromatogram

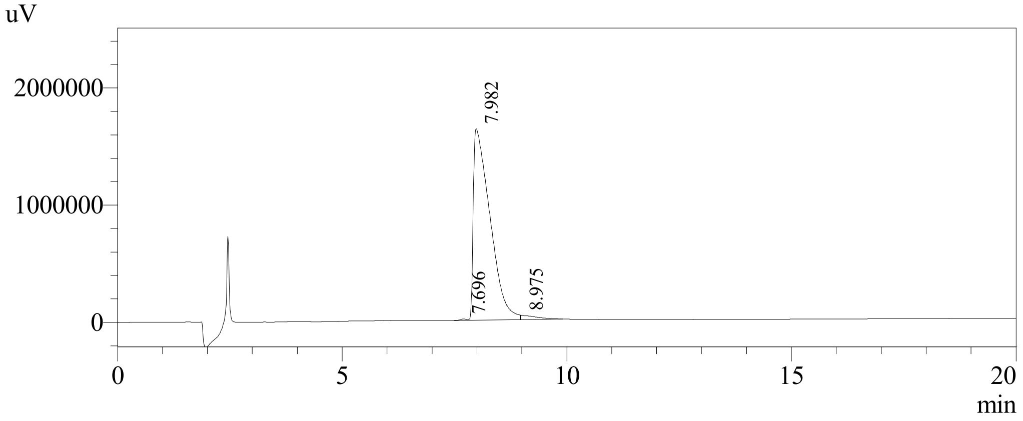

1 Det.A Ch1 / 214nm

PeakTable

Detector A Ch1 214nm

Peak#	Ret. Time	Area	Height	Area %	Height %
1	7.696	114805	12197	0.266	0.725
2	7.982	42171634	1632608	97.743	97.073
3	8.975	858771	37033	1.990	2.202
Total		43145209	1681838	100.000	100.000

生工生物工程（上海）股份有限公司

地址: 上海市松江区香闵路698号
电话/Tel: 400-821-0268
邮箱/Email: sales@sangon.com
Add: 698 Xiang Min Road SongJiang Shanghai China
传真/Fax: 86-21-37772170
网址/Web: www.sangon.com

Sample Information

Name : P20571-8 (Pep133-NFL)
Sequence : SAAERKHRHLPVADANFL
Modification : N/A
Lot.No : P20571- 8 (Pep133-NFL)
Pump A : 0.1%trifluoroacetic in 100%water
Pump B : 0.1%trifluoroacetic in 100%acetonrtrile
Total Flow : 1.0ml/min
Wavelength : 214nm
Analytical column type : NanoChrom Chromcore TM120 C18(4.6*250MM*5UM)
Dissolution method : 0.5mg sample dissolved to 0.5mL by 100%H2O
Inj.Volume : 60ul

Time	Module	Action	Value
0.01	Pumps	B.Conc	18
20.00	Pumps	B.Conc	38

Chromatogram

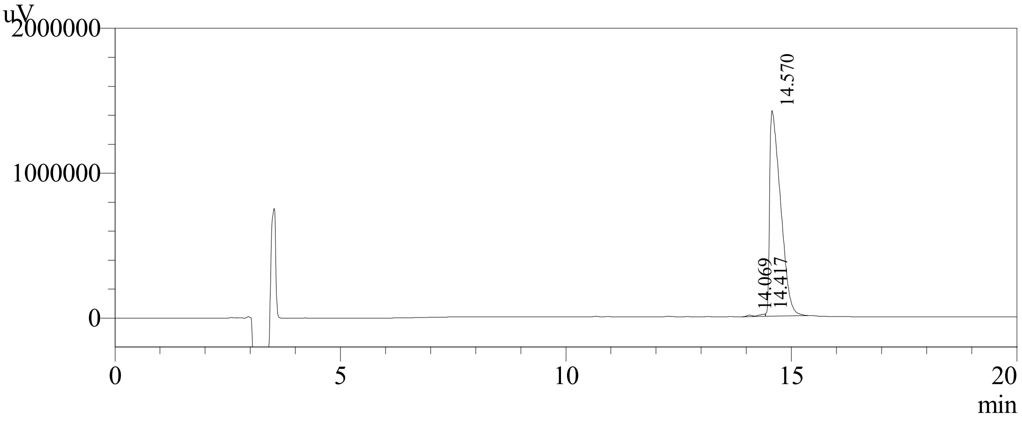

1 Det.A Ch1 / 214nm

PeakTable

Detector A Ch1 214nm

Peak#	Ret. Time	Area	Height	Area %	Height %
1	14.069	81922	10399	0.337	0.719
2	14.417	153332	15581	0.632	1.078
3	14.570	24038251	1420063	99.031	98.203
Total		24273505	1446043	100.000	100.000

生工生物工程（上海）股份有限公司

地址: 上海市松江区香闵路698号
电话/Tel: 400-821-0268
邮箱/Email: sales@sangon.com

Add: 698 Xiang Min Road SongJiang Shanghai China
传真/Fax: 86-21-37772170
网址/Web: www.sangon.com

生工® Sangon Biotech

Sample Information

Name	:	P20571-9 (Pep133-NDL)
Sequence	:	SAAERKHRHLPVADANDL
Modification	:	N/A
Lot.No	:	P20571-9 (Pep133-NDL)
Pump A	:	0.1%trifluoroacetic in 100%water
Pump B	:	0.1%trifluoroacetic in 100%acetonrtrile
Total Flow	:	1.0ml/min
Wavelength	:	214nm
Analytical column type	:	SHIMADZU Inertsil ODS-SP(4.6*250MM*5UM)
Dissolution method	:	0.5mg sample dissolved to 0.5mL by 100%H2O
Inj.Volume	:	60ul

Time	Module	Action	Value
0.01	Pumps	B.Conc	16
20.00	Pumps	B.Conc	36

Chromatogram

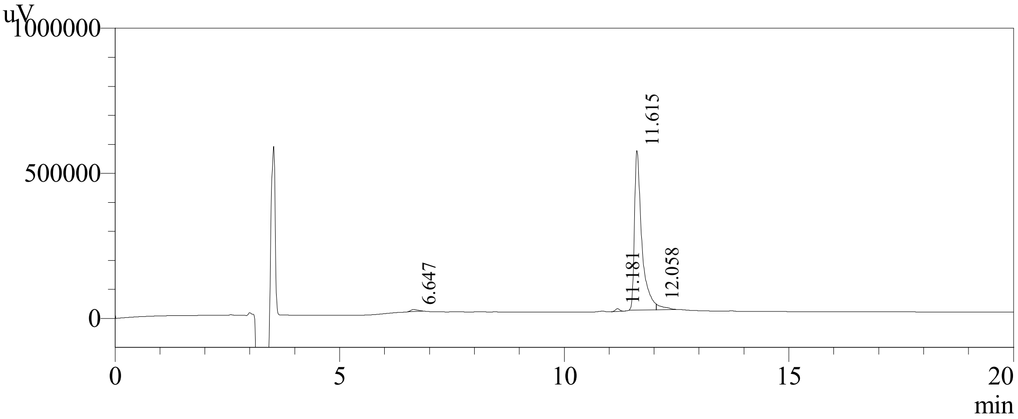

1 Det.A Ch1 / 214nm

PeakTable

Detector A Ch1 214nm

Peak#	Ret. Time	Area	Height	Area %	Height %
1	6.647	81975	7060	1.253	1.208
2	11.181	64730	9502	0.989	1.625
3	11.615	6209662	549908	94.894	94.074
4	12.058	187443	18082	2.864	3.093
Total		6543808	584551	100.000	100.000

生工生物工程（上海）股份有限公司

地址: 上海市松江区香闵路698号
电话/Tel: 400-821-0268
邮箱/Email: sales@sangon.com

Add: 698 Xiang Min Road SongJiang Shanghai China
传真/Fax: 86-21-37772170
网址/Web: www.sangon.com

Sample Information

Name	:	P20571-10 (Pep133-NKL)
Sequence	:	SAAERKHRHLPVADANKL
Modification	:	N/A
Lot.No	:	P20571-10 (Pep133-NKL)
Pump A	:	0.1%trifluoroacetic in 100%water
Pump B	:	0.1%trifluoroacetic in 100%acetonrtrile
Total Flow	:	1.0ml/min
Wavelength	:	214nm
Analytical column type	:	SHIMADZU Inertsil ODS-SP(4.6*150MM*5UM)
Dissolution method	:	0.5mg sample dissolved to 0.5mL by 100%H2O
Inj.Volume	:	60ul

Time	Module	Action	Value
0.01	Pumps	B.Conc	14
20.00	Pumps	B.Conc	34

Chromatogram

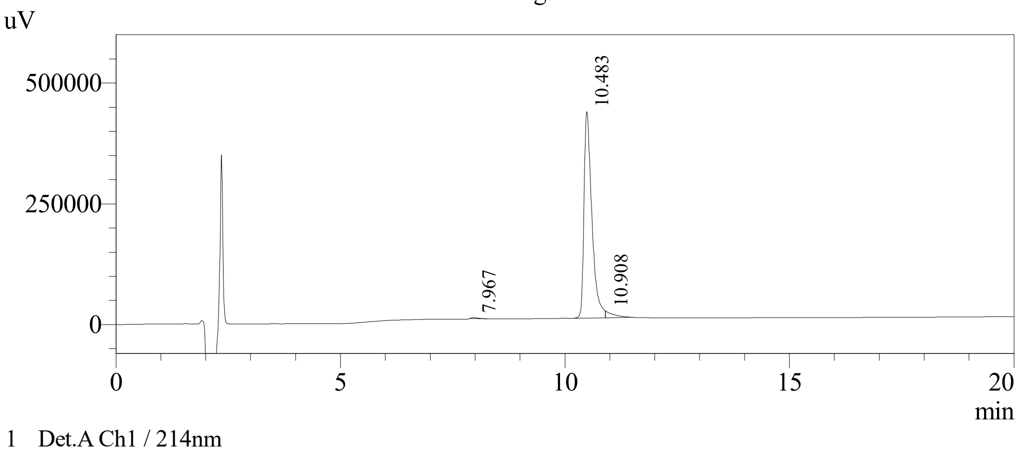

1 Det.A Ch1 / 214nm

PeakTable

Detector A Ch1 214nm

Peak#	Ret. Time	Area	Height	Area %	Height %
1	7.967	19782	2108	0.367	0.476
2	10.483	5201839	426732	96.482	96.376
3	10.908	169867	13941	3.151	3.148
Total		5391488	442781	100.000	100.000

生工生物工程（上海）股份有限公司

地址: 上海市松江区香闵路698号
电话/Tel: 400-821-0268
邮箱/Email: sales@sangon.com

Add: 698 Xiang Min Road SongJiang Shanghai China
传真/Fax: 86-21-37772170
网址/Web: www.sangon.com

Sample Information

Name : P20571-11 (Pep133-NGA)
Sequence : SAAERKHRHLPVADANGA
Modification : N/A
Lot.No : P20571-11 (Pep133-NGA)
Pump A : 0.1%trifluoroacetic in 100%water
Pump B : 0.1%trifluoroacetic in 100%acetonrtrile
Total Flow : 1.0ml/min
Wavelength : 214nm
Analytical column type : NanoChrom Chromcore TM120 C18(4.6*250MM*5UM)
Dissolution method : 0.5mg sample dissolved to 0.5mL by 100%H2O
Inj.Volume : 60ul

Time	Module	Action	Value
0.01	Pumps	B.Conc	10
20.00	Pumps	B.Conc	30

Chromatogram

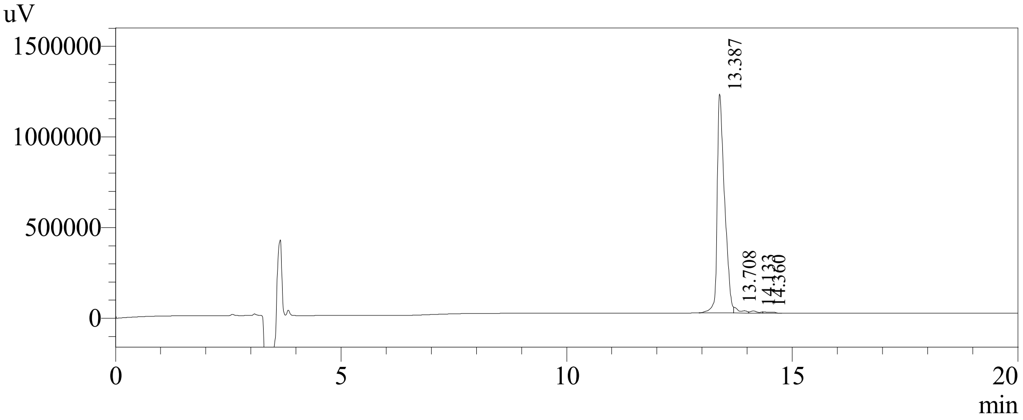

1 Det.A Ch1 / 214nm

PeakTable

Detector A Ch1 214nm

Peak#	Ret. Time	Area	Height	Area %	Height %
1	13.387	13776377	1207727	96.319	96.032
2	13.708	293988	30953	2.055	2.461
3	14.133	139950	11996	0.978	0.954
4	14.360	92582	6957	0.647	0.553
Total		14302897	1257634	100.000	100.000

生工生物工程（上海）股份有限公司

地址: 上海市松江区香闵路698号
电话/Tel: 400-821-0268
邮箱/Email: sales@sangon.com
Add: 698 Xiang Min Road SongJiang Shanghai China
传真/Fax: 86-21-37772170
网址/Web: www.sangon.com

生工® Sangon Biotech

Sample Information

Name	:	P20571-12 (Pep133-NGQ)
Sequence	:	SAAERKHRHLPVADANGQ
Modification	:	N/A
Lot.No	:	P20571-12 (Pep133-NGQ)
Pump A	:	0.1%trifluoroacetic in 100%water
Pump B	:	0.1%trifluoroacetic in 100%acetonrtrile
Total Flow	:	1.0ml/min
Wavelength	:	214nm
Analytical column type	:	SHIMADZU Inertsil ODS-SP(4.6*250MM*5UM)
Dissolution method	:	0.5mg sample dissolved to 0.5mL by 100%H2O
Inj.Volume	:	60ul

Time	Module	Action	Value
0.01	Pumps	B.Conc	12
20.00	Pumps	B.Conc	32

Chromatogram

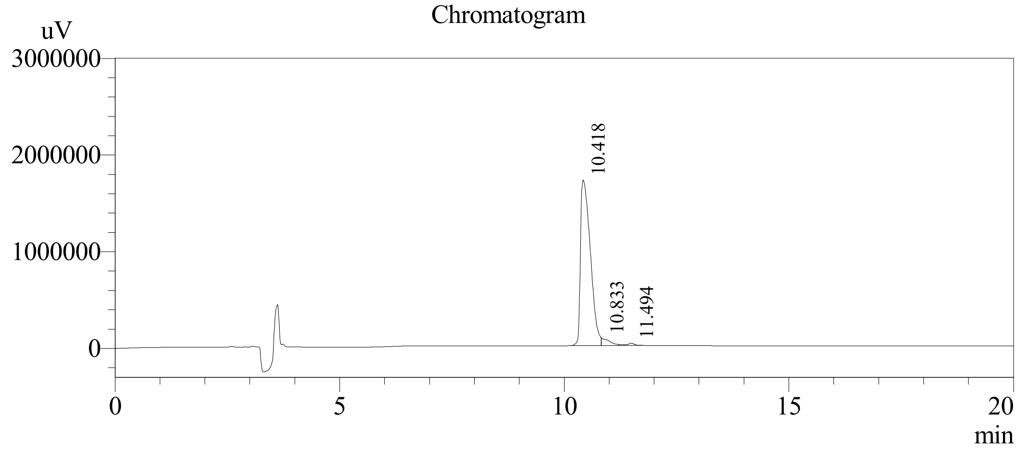

1 Det.A Ch1 / 214nm

PeakTable

Detector A Ch1 214nm

Peak#	Ret. Time	Area	Height	Area %	Height %
1	10.418	26226319	1714292	95.553	94.447
2	10.833	937595	77690	3.416	4.280
3	11.494	282948	23097	1.031	1.272
Total		27446862	1815079	100.000	100.000

生工生物工程（上海）股份有限公司

地址: 上海市松江区香闵路698号 Add: 698 Xiang Min Road SongJiang Shanghai China
电话/Tel: 400-821-0268 传真/Fax: 86-21-37772170
邮箱/Email: sales@sangon.com 网址/Web: www.sangon.com

生工® Sangon Biotech

Sample Information

Name : P20571-13 (Pep133-NGF)
Sequence : SAAERKHRHLPVADANGF
Modification : N/A
Lot.No : P20571-13 (Pep133-NGF)
Pump A : 0.1%trifluoroacetic in 100%water
Pump B : 0.1%trifluoroacetic in 100%acetonrtrile
Total Flow : 1.0ml/min
Wavelength : 214nm
Analytical column type : NanoChrom Chromcore TM120 C18(4.6*250MM*5UM)
Dissolution method : 0.5mg sample dissolved to 0.5mL by 100%H2O
Inj.Volume : 60ul

Time	Module	Action	Value
0.01	Pumps	B.Conc	18
20.00	Pumps	B.Conc	38

Chromatogram

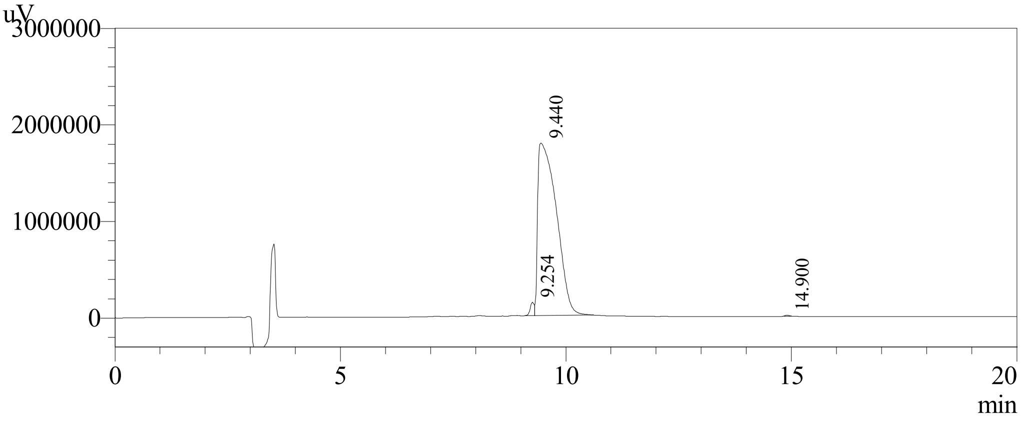

1 Det.A Ch1 / 214nm

PeakTable

Detector A Ch1 214nm

Peak#	Ret. Time	Area	Height	Area %	Height %
1	9.254	862905	138069	1.648	7.131
2	9.440	51415958	1786601	98.178	92.268
3	14.900	91271	11648	0.174	0.602
Total		52370134	1936319	100.000	100.000

生工生物工程（上海）股份有限公司

地址: 上海市松江区香闵路698号
电话/Tel: 400-821-0268
邮箱/Email: sales@sangon.com

Add: 698 Xiang Min Road SongJiang Shanghai China
传真/Fax: 86-21-37772170
网址/Web: www.sangon.com

生工® Sangon Biotech

Sample Information

Name	:	P20571-14 (Pep133-NGD)
Sequence	:	SAAERKHRHLPVADANGD
Modification	:	N/A
Lot.No	:	P20571-14 (Pep133-NGD)
Pump A	:	0.1%trifluoroacetic in 100%water
Pump B	:	0.1%trifluoroacetic in 100%acetonrtrile
Total Flow	:	1.0ml/min
Wavelength	:	214nm
Analytical column type	:	SHIMADZU Inertsil ODS-SP(4.6*250MM*5UM)
Dissolution method	:	0.5mg sample dissolved to 0.5mL by 100%H2O
Inj.Volume	:	60ul

Time	Module	Action	Value
0.01	Pumps	B.Conc	15
20.00	Pumps	B.Conc	35

Chromatogram

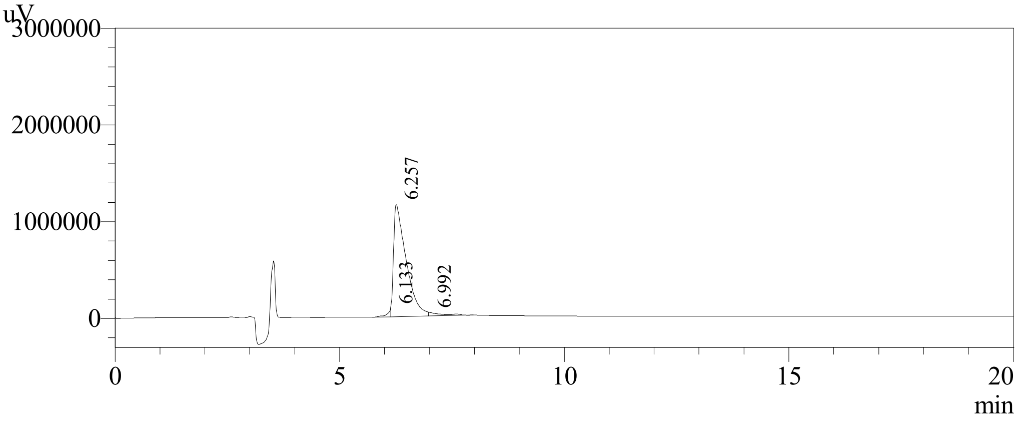

1 Det.A Ch1 / 214nm

PeakTable

Detector A Ch1 214nm

Peak#	Ret. Time	Area	Height	Area %	Height %
1	6.133	467571	83791	1.976	6.540
2	6.257	22477288	1159017	94.978	90.467
3	6.992	720854	38345	3.046	2.993
Total		23665713	1281153	100.000	100.000

生工生物工程（上海）股份有限公司

地址: 上海市松江区香闵路698号 Add: 698 Xiang Min Road SongJiang Shanghai China
电话/Tel: 400-821-0268 传真/Fax: 86-21-37772170
邮箱/Email: sales@sangon.com 网址/Web: www.sangon.com

Sample Information

Name	:	P20571-15 (Pep133-NGK)
Sequence	:	SAAERKHRHLPVADANGK
Modification	:	N/A
Lot.No	:	P20571-15 (Pep133-NGK)
Pump A	:	0.1%trifluoroacetic in 100%water
Pump B	:	0.1%trifluoroacetic in 100%acetonrtrile
Total Flow	:	1.0ml/min
Wavelength	:	214nm
Analytical column type	:	SHIMADZU Inertsil ODS-SP(4.6*250MM*5UM)
Dissolution method	:	0.5mg sample dissolved to 0.5mL by 100%H2O
Inj.Volume	:	60ul

Time	Module	Action	Value
0.01	Pumps	B.Conc	12
20.00	Pumps	B.Conc	32
20.01	Controller	Stop	

Chromatogram

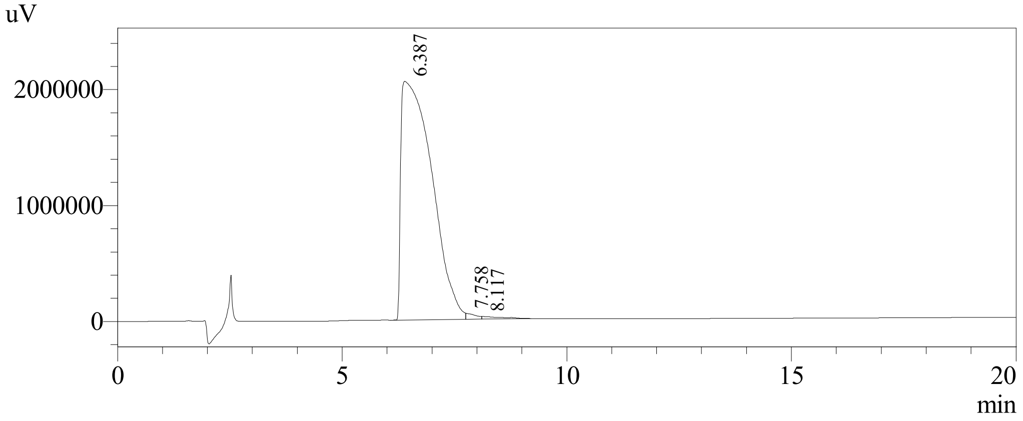

1 Det.A Ch1 / 214nm

PeakTable

Detector A Ch1 214nm

Peak#	Ret. Time	Area	Height	Area %	Height %
1	6.387	98760111	2058691	98.547	96.531
2	7.758	775378	51899	0.774	2.434
3	8.117	680602	22082	0.679	1.035
Total		100216090	2132671	100.000	100.000

生工生物工程（上海）股份有限公司

地址: 上海市松江区香闵路698号
电话/Tel: 400-821-0268
邮箱/Email: sales@sangon.com

Add: 698 Xiang Min Road SongJiang Shanghai China
传真/Fax: 86-21-37772170
网址/Web: www.sangon.com

Sample Information

Name	:	P21421-3-Pep133-NVL
Sequence	:	SAAERKHRHLPVADANVL
Modification	:	N/A
Lot.No	:	P21421-3-Pep133-NVL
Pump A	:	0.1%trifluoroacetic in 100%water
Pump B	:	0.1%trifluoroacetic in 100%acetonrtrile
Total Flow	:	1.0ml/min
Wavelength	:	214nm
Analytical column type	:	SHIMADZU Inertsil ODS-SP(4.6*250MM*5UM)
Dissolution method	:	0.5mg sample dissolved to 0.5mL by 100%H2O
Inj.Volume	:	60ul

Time	Module	Action	Value
0.01	Pumps	B.Conc	18
20.00	Pumps	B.Conc	38

Chromatogram

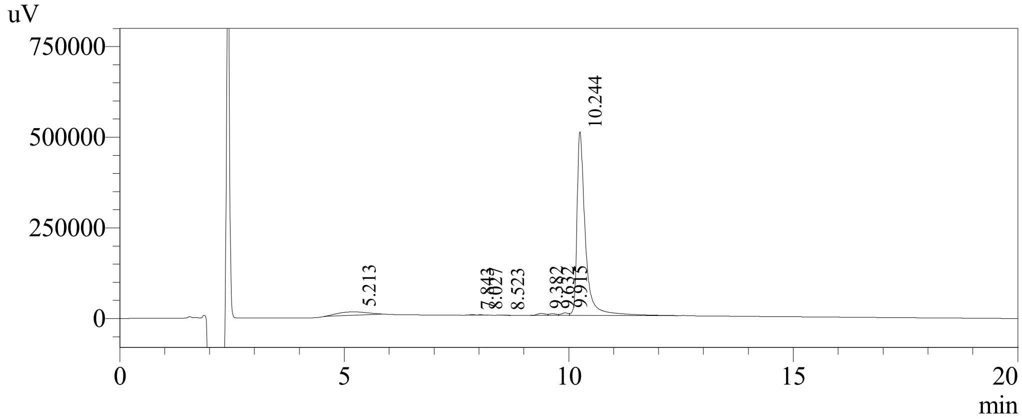

1 Det.A Ch1 / 214nm

PeakTable

Detector A Ch1 214nm

Peak#	Ret. Time	Area	Height	Area %	Height %
1	5.213	444761	9451	6.096	1.764
2	7.843	9901	1182	0.136	0.221
3	8.027	7331	763	0.100	0.142
4	8.523	4811	594	0.066	0.111
5	9.382	75993	5651	1.041	1.055
6	9.632	53261	4570	0.730	0.853
7	9.915	79589	7339	1.091	1.370
8	10.244	6620871	506128	90.740	94.484
Total		7296517	535678	100.000	100.000

生工生物工程（上海）股份有限公司

地址: 上海市松江区香闵路698号 Add: 698 Xiang Min Road SongJiang Shanghai China
电话/Tel: 400-821-0268 传真/Fax: 86-21-37772170
邮箱/Email: sales@sangon.com 网址/Web: www.sangon.com

Sample Information

Name	:	P21421-4-Pep133-NLL
Sequence	:	SAAERKHRHLPVADANLL
Modification	:	N/A
Lot.No	:	P21421-4-Pep133-NLL
Pump A	:	0.1%trifluoroacetic in 100%water
Pump B	:	0.1%trifluoroacetic in 100%acetonrtrile
Total Flow	:	1.0ml/min
Wavelength	:	214nm
Analytical column type	:	NanoChrom Chromcore TM120 C18(4.6*250MM*5UM)
Dissolution method	:	0.1mg sample dissolved to 0.5mL by 100%H2O
Inj.Volume	:	60ul

Time	Module	Action	Value
0.01	Pumps	B.Conc	23
20.01	Pumps	B.Conc	43

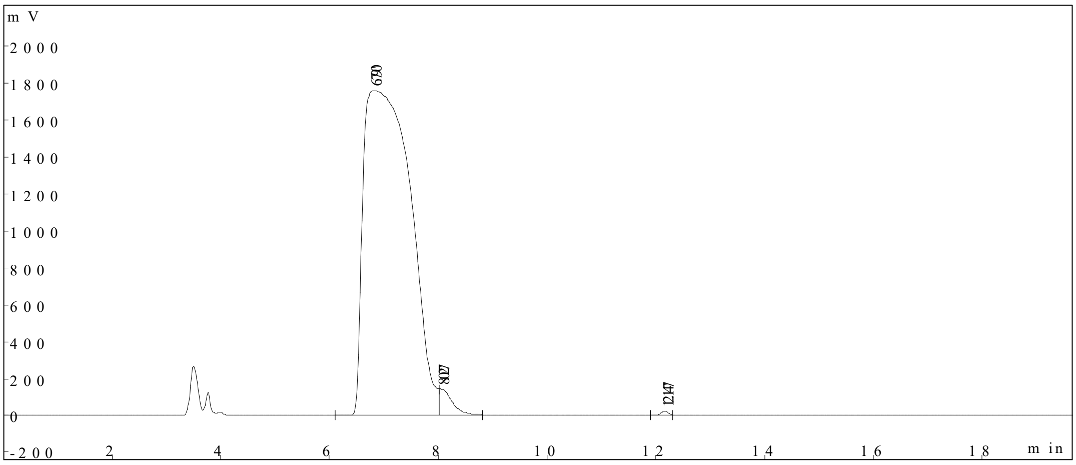

PeakTable

Detector A Ch 214nm

Peak#	Ret.Time	Area	Height	Area%	Height%
1	6.790	105231098	1760328	97.911	91.474
2	8.027	2049845	141287	1.907	7.342
3	12.147	201706	22784	0.182	1.184
Total		107482649	1924399	100	100

生工生物工程（上海）股份有限公司

地址: 上海市松江区香闵路698号
电话/Tel: 400-821-0268
邮箱/Email: sales@sangon.com

Add: 698 Xiang Min Road SongJiang Shanghai China
传真/Fax: 86-21-37772170
网址/Web: www.sangon.com

Sample Information

Name	:	P21421-6-Pep133-NPL
Sequence	:	SAAERKHRHLPVADANPL
Modification	:	N/A
Lot.No	:	P21421-6-Pep133-NPL
Pump A	:	0.1%trifluoroacetic in 100%water
Pump B	:	0.1%trifluoroacetic in 100%acetonrtrile
Total Flow	:	1.0ml/min
Wavelength	:	214nm
Analytical column type	:	SHIMADZU Inertsil ODS-SP(4.6*250MM*5UM)
Dissolution method	:	0.5mg sample dissolved to 0.5mL by 100%H2O
Inj.Volume	:	60ul

Time	Module	Action	Value
0.01	Pumps	B.Conc	16
20.00	Pumps	B.Conc	36

Chromatogram

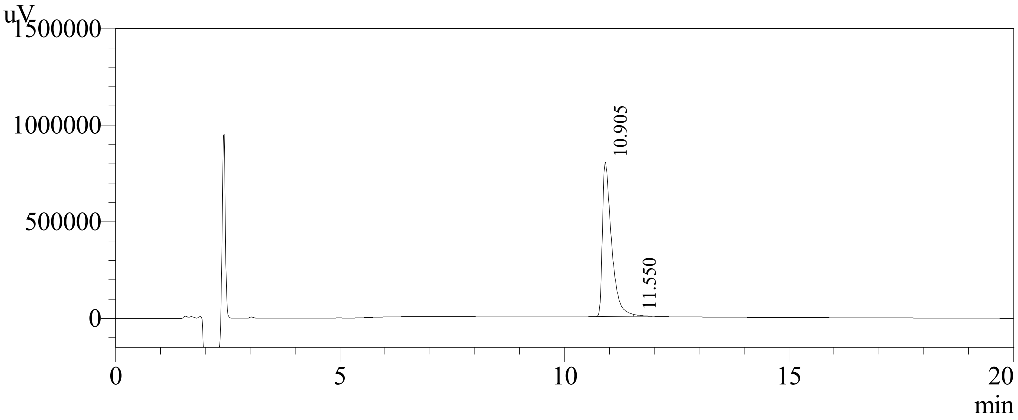

1 Det.A Ch1 / 214nm

PeakTable

Detector A Ch1 214nm

Peak#	Ret. Time	Area	Height	Area %	Height %
1	10.905	11257265	798273	99.262	98.836
2	11.550	83654	9399	0.738	1.164
Total		11340920	807673	100.000	100.000

生工生物工程（上海）股份有限公司

地址: 上海市松江区香闵路698号
电话/Tel: 400-821-0268
邮箱/Email: sales@sangon.com
Add: 698 Xiang Min Road SongJiang Shanghai China
传真/Fax: 86-21-37772170
网址/Web: www.sangon.com

Sample Information

Name	:	P27704-2-Pep133-NAL(Fam labeled)
Sequence	:	SAAERKHRHLPVADAK（Fam）NAL
Modification	:	K（Fam）
Lot.No	:	P27704-2-Pep133-NAL(Fam labeled)
Pump A	:	0.1%trifluoroacetic in 100%water
Pump B	:	0.1%trifluoroacetic in 100%acetonrtrile
Total Flow	:	1.0ml/min
Wavelength	:	214nm
Analytical column type	:	SHIMADZU shim-pack GIST(4.6*250MM*5UM)
Dissolution method	:	0.1mg sample dissolved to 0.5mL by 5%HCOOH 20%ACN and 75%H2O
Inj.Volume	:	30ul

Time	Module	Action	Value
0.01	Pumps	B.Conc	27
20.00	Pumps	B.Conc	47

Chromatogram

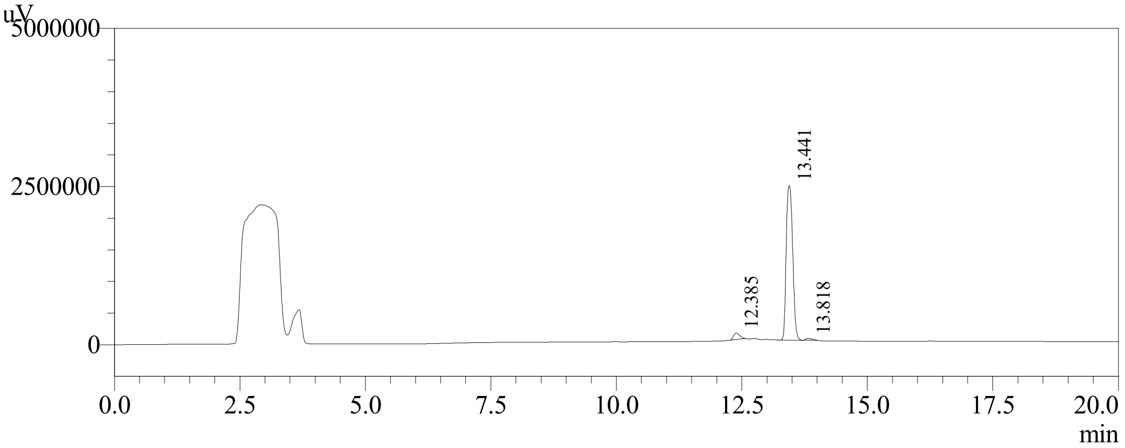

1 Det.A Ch1 / 214nm

PeakTable

Detector A Ch1 214nm

Peak#	Ret. Time	Area	Height	Area %	Height %
1	12.385	878283	100745	3.835	3.919
2	13.441	21773957	2441522	95.083	94.975
3	13.818	247687	28440	1.082	1.106
Total		22899927	2570707	100.000	100.000

生工生物工程（上海）股份有限公司

地址: 上海市松江区香闵路698号
电话/Tel: 400-821-0268
邮箱/Email: sales@sangon.com

Add: 698 Xiang Min Road SongJiang Shanghai China
传真/Fax: 86-21-37772170
网址/Web: www.sangon.com

生工® Sangon Biotech

Sample Information

Name	:	P20955-1 (C50-NAL)
Sequence	:	PQRKTKRNTNRRPQDVKFPGGGQIVGGVYLLPRRGPRLGVRATRKTSERSNAL
Modification	:	N/A
Lot.No	:	P20955-1 (C50-NAL)
Pump A	:	0.1%trifluoroacetic in 100%water
Pump B	:	0.1%trifluoroacetic in 100%acetonrtrile
Total Flow	:	1.0ml/min
Wavelength	:	214nm
Analytical column type	:	NanoChrom Chromcore TM120 C18(4.6*250MM*5UM)
Dissolution method	:	0.5mg sample dissolved to 0.5mL by 100%H2O
Inj.Volume	:	60ul

Time	Module	Action	Value
0.01	Pumps	B.Conc	19
20.00	Pumps	B.Conc	39

Chromatogram

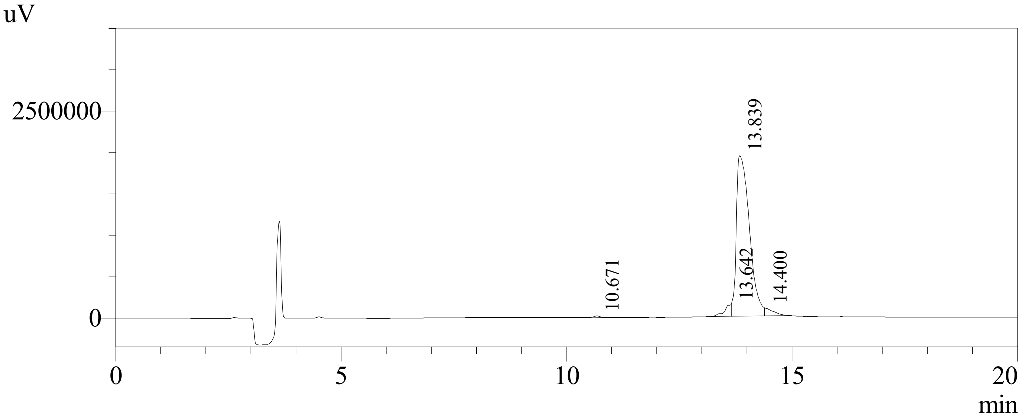

1 Det.A Ch1 / 214nm

PeakTable

Detector A Ch1 214nm

Peak#	Ret. Time	Area	Height	Area %	Height %
1	10.671	139732	17618	0.332	0.804
2	13.642	1455869	140799	3.459	6.426
3	13.839	39234370	1937672	93.211	88.431
4	14.400	1262091	95091	2.998	4.340
Total		42092061	2191180	100.000	100.000

生工生物工程（上海）股份有限公司

地址: 上海市松江区香闵路698号
电话/Tel: 400-821-0268
邮箱/Email: sales@sangon.com

Add: 698 Xiang Min Road SongJiang Shanghai China
传真/Fax: 86-21-37772170
网址/Web: www.sangon.com

Sample Information

Name	:	P16698（GL-(biotin-labeled peptide)）
Sequence	:	GLPV(K-Biotin)AR
Modification	:	K-Biotin
Lot.No	:	P16698（GL-(biotin-labeled peptide)）
Pump A	:	0.1%trifluoroacetic in 100%water
Pump B	:	0.1%trifluoroacetic in 100%acetonrtrile
Total Flow	:	1.0ml/min
Wavelength	:	214nm
Analytical column type	:	SHIMADZU Inertsil ODS-SP(4.6*250MM*5UM)
Dissolution method	:	0.1mg sample dissolved to 0.5mL by 100%H2O
Inj.Volume	:	70ul

Time	Module	Action	Value
0.01	Pumps	B.Conc	11
20.00	Pumps	B.Conc	31
20.01	Pumps	B.Conc	95
27.01	Pumps	B.Conc	95
27.02	Controller	Stop	

Chromatogram

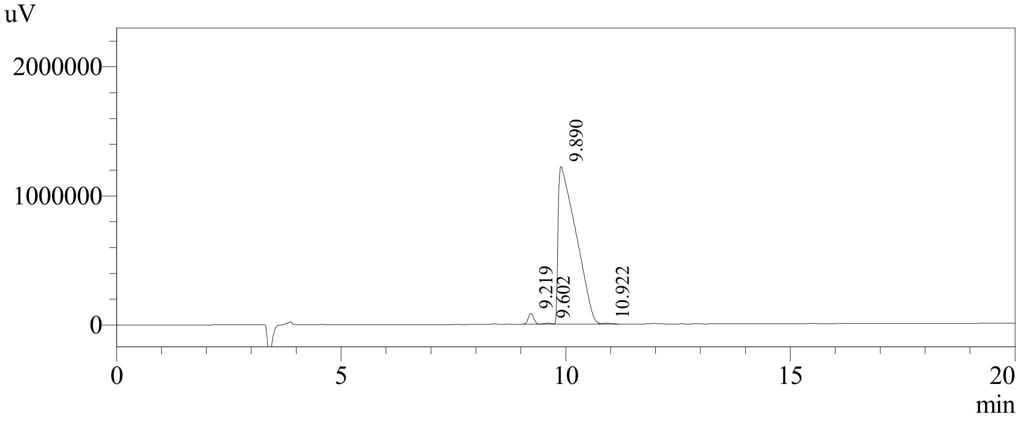

1 Det.A Ch1 / 214nm

PeakTable

Detector A Ch1 214nm

Peak#	Ret. Time	Area	Height	Area %	Height %
1	9.219	718653	83695	2.075	6.337
2	9.602	92937	8560	0.268	0.648
3	9.890	33716598	1221273	97.346	92.476
4	10.922	107473	7110	0.310	0.538
Total		34635662	1320639	100.000	100.000

生工® Sangon Biotech

Sample Information

Name	:	P20955-2（AL-(biotin-labeled) peptide）
Sequence	:	ALPVKAR
Modification	:	K-Biotin
Lot.No	:	P20955-2（AL-(biotin-labeled) peptide）
Pump A	:	0.1%trifluoroacetic in 100%water
Pump B	:	0.1%trifluoroacetic in 100%acetonrtrile
Total Flow	:	1.0ml/min
Wavelength	:	214nm
Analytical column type	:	SHIMADZU Inertsil ODS-SP(4.6*150MM*5UM)
Dissolution method	:	0.5mg sample dissolved to 0.5mL by 100%H2O
Inj.Volume	:	60ul

Time	Module	Action	Value
0.01	Pumps	B.Conc	19
20.00	Pumps	B.Conc	39

Chromatogram

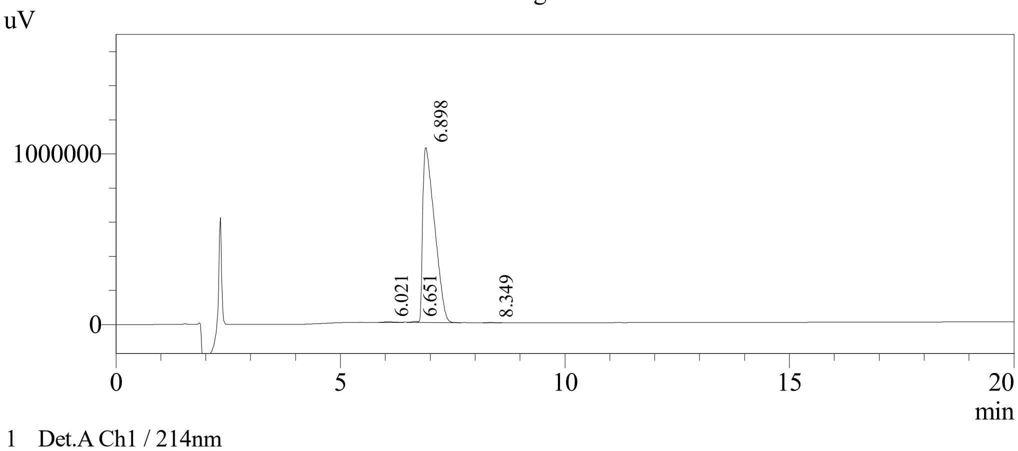

1 Det.A Ch1 / 214nm

PeakTable

Detector A Ch1 214nm

Peak#	Ret. Time	Area	Height	Area %	Height %
1	6.021	47183	3420	0.250	0.330
2	6.651	43434	5527	0.230	0.534
3	6.898	18781745	1024622	99.453	98.964
4	8.349	12719	1780	0.067	0.172
Total		18885081	1035350	100.000	100.000

生工生物工程（上海）股份有限公司

地址: 上海市松江区香闵路698号
电话/Tel: 400-821-0268
邮箱/Email: sales@sangon.com

Add: 698 Xiang Min Road SongJiang Shanghai China
传真/Fax: 86-21-37772170
网址/Web: www.sangon.com

Sangon Biotech

Sample Information

Name	:	P20955-3（QL-(biotin-labeled) peptide）
Sequence	:	QLPVKAR
Modification	:	K-Biotin
Lot.No	:	P20955-3（QL-(biotin-labeled) peptide）
Pump A	:	0.1%trifluoroacetic in 100%water
Pump B	:	0.1%trifluoroacetic in 100%acetonrtrile
Total Flow	:	1.0ml/min
Wavelength	:	214nm
Analytical column type	:	SHIMADZU Inertsil ODS-SP(4.6*150MM*5UM)
Dissolution method	:	0.5mg sample dissolved to 0.5mL by 100%H2O
Inj.Volume	:	60ul

Time	Module	Action	Value
0.01	Pumps	B.Conc	20
20.00	Pumps	B.Conc	40

Chromatogram

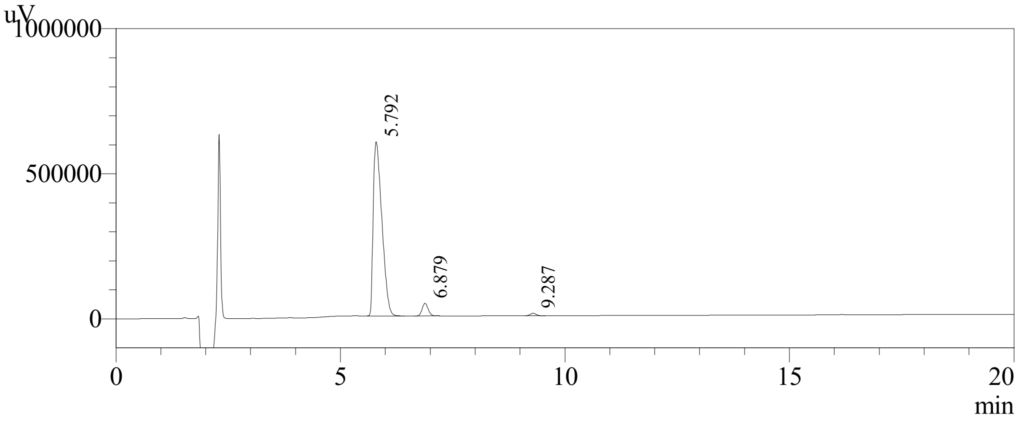

1 Det.A Ch1 / 214nm

PeakTable

Detector A Ch1 214nm

Peak#	Ret. Time	Area	Height	Area %	Height %
1	5.792	8073977	600155	94.359	92.004
2	6.879	409340	43756	4.784	6.708
3	9.287	73336	8405	0.857	1.288
Total		8556653	652316	100.000	100.000

生工生物工程（上海）股份有限公司

地址: 上海市松江区香闵路698号
电话/Tel: 400-821-0268
邮箱/Email: sales@sangon.com

Add: 698 Xiang Min Road SongJiang Shanghai China
传真/Fax: 86-21-37772170
网址/Web: www.sangon.com

生工® Sangon Biotech

Sample Information

Name	:	P20955-4（FL-(biotin-labeled) peptide）
Sequence	:	FLPVKAR
Modification	:	K-Biotin
Lot.No	:	P20955-4（FL-(biotin-labeled) peptide）
Pump A	:	0.1%trifluoroacetic in 100%water
Pump B	:	0.1%trifluoroacetic in 100%acetonrtrile
Total Flow	:	1.0ml/min
Wavelength	:	214nm
Analytical column type	:	SHIMADZU Inertsil ODS-SP(4.6*150MM*5UM)
Dissolution method	:	0.5mg sample dissolved to 0.5mL by 100%H2O
Inj.Volume	:	60ul

Time	Module	Action	Value
0.01	Pumps	B.Conc	21
20.00	Pumps	B.Conc	41

Chromatogram

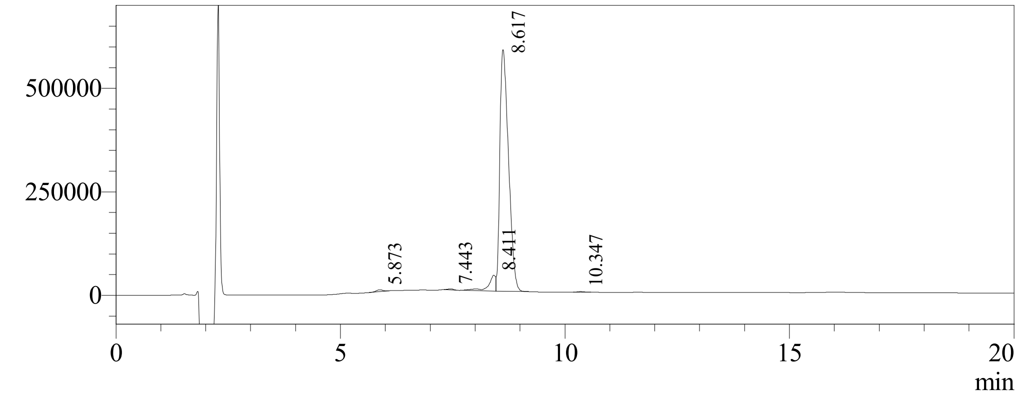

1 Det.A Ch1 / 214nm

PeakTable

Detector A Ch1 214nm

Peak#	Ret. Time	Area	Height	Area %	Height %
1	5.873	45830	4361	0.548	0.693
2	7.443	22693	2620	0.271	0.416
3	8.411	438429	37987	5.244	6.036
4	8.617	7839877	583001	93.770	92.631
5	10.347	13894	1410	0.166	0.224
Total		8360723	629378	100.000	100.000

生工生物工程（上海）股份有限公司

地址: 上海市松江区香闵路698号
电话/Tel: 400-821-0268
邮箱/Email: sales@sangon.com

Add: 698 Xiang Min Road SongJiang Shanghai China
传真/Fax: 86-21-37772170
网址/Web: www.sangon.com

Sample Information

Name	:	P20955-5（DL-(biotin-labeled) peptide）
Sequence	:	DLPVKAR
Modification	:	K-Biotin
Lot.No	:	P20955-5（DL-(biotin-labeled) peptide）
Pump A	:	0.1%trifluoroacetic in 100%water
Pump B	:	0.1%trifluoroacetic in 100%acetonrtrile
Total Flow	:	1.0ml/min
Wavelength	:	214nm
Analytical column type	:	SHIMADZU Inertsil ODS-SP(4.6*150MM*5UM)
Dissolution method	:	0.5mg sample dissolved to 0.5mL by 100%H2O
Inj.Volume	:	60ul

Time	Module	Action	Value
0.01	Pumps	B.Conc	16
20.00	Pumps	B.Conc	36

Chromatogram

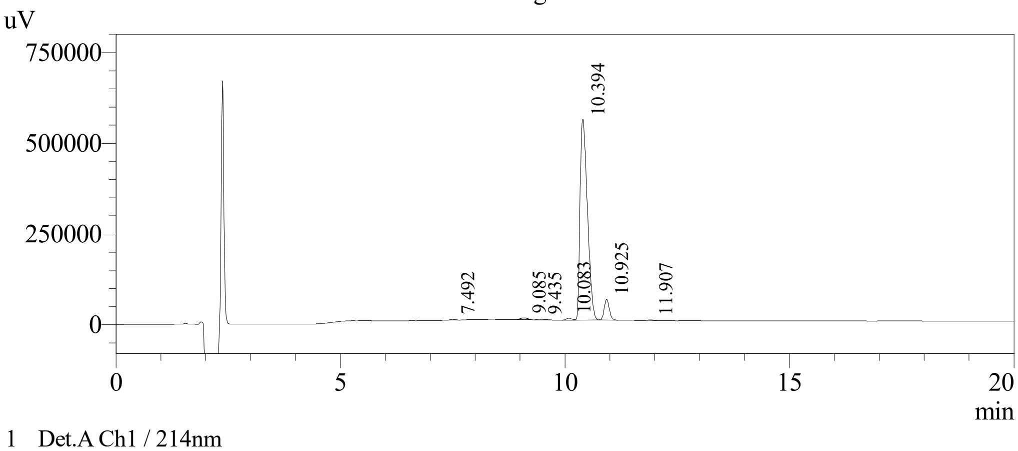

1 Det.A Ch1 / 214nm

PeakTable

Detector A Ch1 214nm

Peak#	Ret. Time	Area	Height	Area %	Height %
1	7.492	8508	1550	0.123	0.248
2	9.085	47277	4655	0.684	0.745
3	9.435	21217	1949	0.307	0.312
4	10.083	40119	4881	0.580	0.781
5	10.394	6331972	553356	91.583	88.523
6	10.925	454369	57077	6.572	9.131
7	11.907	10422	1630	0.151	0.261
Total		6913883	625097	100.000	100.000

生工生物工程（上海）股份有限公司

地址: 上海市松江区香闵路698号
电话/Tel: 400-821-0268
邮箱/Email: sales@sangon.com

Add: 698 Xiang Min Road SongJiang Shanghai China
传真/Fax: 86-21-37772170
网址/Web: www.sangon.com

生工® Sangon Biotech

Sample Information

Name : P20955-6（KL-(biotin-labeled) peptide）
Sequence : KLPVKAR
Modification : K-Biotin
Lot.No : P20955-6（KL-(biotin-labeled) peptide）
Pump A : 0.1%trifluoroacetic in 100%water
Pump B : 0.1%trifluoroacetic in 100%acetonrtrile
Total Flow : 1.0ml/min
Wavelength : 214nm
Analytical column type : SHIMADZU Inertsil ODS-SP(4.6*150MM*5UM)
Dissolution method : 0.5mg sample dissolved to 0.5mL by 100%H2O
Inj.Volume : 60ul

Time	Module	Action	Value
0.01	Pumps	B.Conc	12
20.00	Pumps	B.Conc	32

Chromatogram

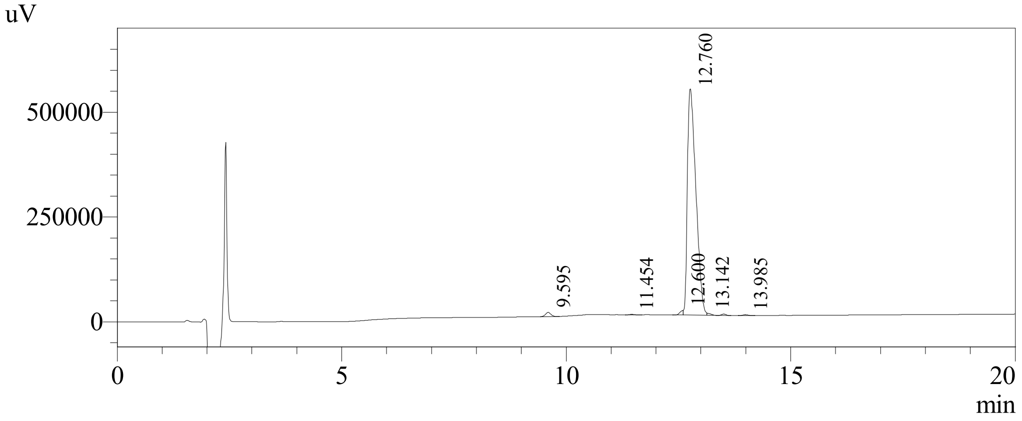

1 Det.A Ch1 / 214nm

PeakTable

Detector A Ch1 214nm

Peak#	Ret. Time	Area	Height	Area %	Height %
1	9.595	82993	9869	1.135	1.735
2	11.454	10937	1402	0.150	0.247
3	12.600	57758	10969	0.790	1.929
4	12.760	7089690	539184	96.924	94.803
5	13.142	56427	5245	0.771	0.922
6	13.985	16884	2072	0.231	0.364
Total		7314689	568741	100.000	100.000

生工生物工程（上海）股份有限公司

地址: 上海市松江区香闵路698号
电话/Tel: 400-821-0268
邮箱/Email: sales@sangon.com
Add: 698 Xiang Min Road SongJiang Shanghai China
传真/Fax: 86-21-37772170
网址/Web: www.sangon.com

生工® Sangon Biotech

Sample Information

Name : P20955-7（GA-(biotin-labeled) peptide）
Sequence : GAPVKAR
Modification : K-Biotin
Lot.No : P20955-7（GA-(biotin-labeled) peptide）
Pump A : 0.1%trifluoroacetic in 100%water
Pump B : 0.1%trifluoroacetic in 100%acetonrtrile
Total Flow : 1.0ml/min
Wavelength : 214nm
Analytical column type : NanoChrom Chromcore TM120 C18(4.6*250MM*5UM)
Dissolution method : 0.5mg sample dissolved to 0.5mL by 100%H2O
Inj.Volume : 60ul

Time	Module	Action	Value
0.01	Pumps	B.Conc	13
20.00	Pumps	B.Conc	33

Chromatogram

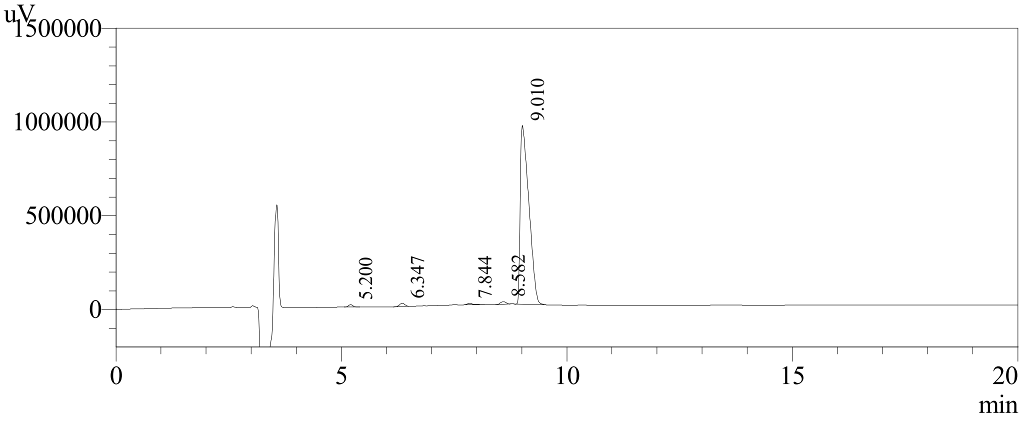

1 Det.A Ch1 / 214nm

PeakTable

Detector A Ch1 214nm

Peak#	Ret. Time	Area	Height	Area %	Height %
1	5.200	80042	12134	0.609	1.209
2	6.347	136844	17023	1.041	1.696
3	7.844	64688	7210	0.492	0.718
4	8.582	118754	13326	0.903	1.327
5	9.010	12749986	954210	96.956	95.050
Total		13150314	1003904	100.000	100.000

生工® Sangon Biotech

Sample Information

Name	:	P20955-8（GQ-(biotin-labeled) peptide）
Sequence	:	GQPVKAR
Modification	:	K-Biotin
Lot.No	:	P20955-8（GQ-(biotin-labeled) peptide）
Pump A	:	0.1%trifluoroacetic in 100%water
Pump B	:	0.1%trifluoroacetic in 100%acetonrtrile
Total Flow	:	1.0ml/min
Wavelength	:	214nm
Analytical column type	:	SHIMADZU Inertsil ODS-SP(4.6*150MM*5UM)
Dissolution method	:	0.5mg sample dissolved to 0.5mL by 100%H2O
Inj.Volume	:	60ul

Time	Module	Action	Value
0.01	Pumps	B.Conc	12
20.00	Pumps	B.Conc	32

Chromatogram

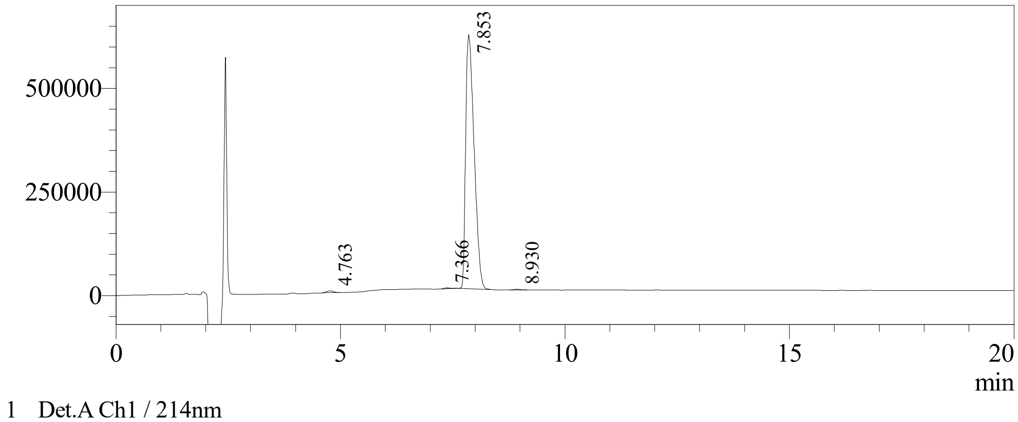

1 Det.A Ch1 / 214nm

PeakTable

Detector A Ch1 214nm

Peak#	Ret. Time	Area	Height	Area %	Height %
1	4.763	48779	4703	0.601	0.756
2	7.366	20522	2133	0.253	0.343
3	7.853	8033086	613291	98.957	98.610
4	8.930	15330	1809	0.189	0.291
Total		8117717	621936	100.000	100.000

生工生物工程（上海）股份有限公司

地址: 上海市松江区香闵路698号
电话/Tel: 400-821-0268
邮箱/Email: sales@sangon.com

Add: 698 Xiang Min Road SongJiang Shanghai China
传真/Fax: 86-21-37772170
网址/Web: www.sangon.com

生工® Sangon Biotech

Sample Information

Name	:	P20955-9（GF-(biotin-labeled) peptide）
Sequence	:	GFPVKAR
Modification	:	K-Biotin
Lot.No	:	P20955-9（GF-(biotin-labeled) peptide）
Pump A	:	0.1%trifluoroacetic in 100%water
Pump B	:	0.1%trifluoroacetic in 100%acetonrtrile
Total Flow	:	1.0ml/min
Wavelength	:	214nm
Analytical column type	:	SHIMADZU Inertsil ODS-SP(4.6*150MM*5UM)
Dissolution method	:	0.5mg sample dissolved to 0.5mL by 100%H2O

Inj.Volume : 60ul

Time	Module	Action	Value
0.01	Pumps	B.Conc	18
20.00	Pumps	B.Conc	38

Chromatogram

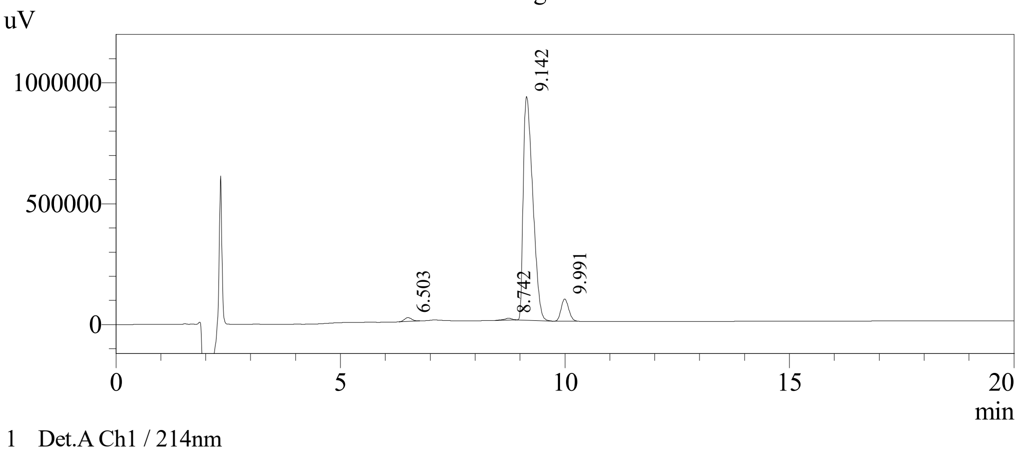

1 Det.A Ch1 / 214nm

PeakTable

Detector A Ch1 214nm

Peak#	Ret. Time	Area	Height	Area %	Height %
1	6.503	175515	15675	1.168	1.507
2	8.742	92715	7445	0.617	0.716
3	9.142	13702723	925410	91.184	88.992
4	9.991	1056663	91351	7.031	8.785
Total		15027616	1039881	100.000	100.000

生工生物工程（上海）股份有限公司

地址: 上海市松江区香闵路698号
电话/Tel: 400-821-0268
邮箱/Email: sales@sangon.com

Add: 698 Xiang Min Road SongJiang Shanghai China
传真/Fax: 86-21-37772170
网址/Web: www.sangon.com

生工® Sangon Biotech

Sample Information

Name	:	P20955-10（GD-(biotin-labeled) peptide）
Sequence	:	GDPVKAR
Modification	:	K-Biotin
Lot.No	:	P20955-10（GD-(biotin-labeled) peptide）
Pump A	:	0.1%trifluoroacetic in 100%water
Pump B	:	0.1%trifluoroacetic in 100%acetonrtrile
Total Flow	:	1.0ml/min
Wavelength	:	214nm
Analytical column type	:	NanoChrom Chromcore TM120 C18(4.6*250MM*5UM)
Dissolution method	:	0.5mg sample dissolved to 0.5mL by 100%H2O
Inj.Volume	:	60ul

Time	Module	Action	Value
0.01	Pumps	B.Conc	16
20.00	Pumps	B.Conc	36

Chromatogram

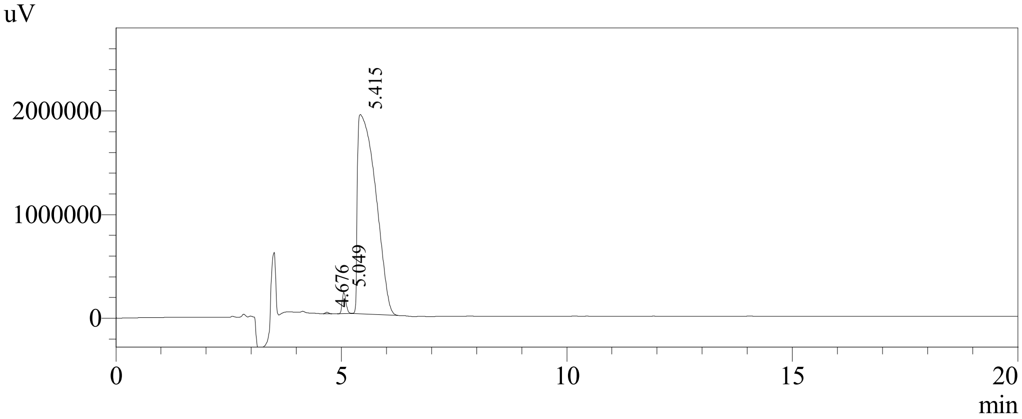

1 Det.A Ch1 / 214nm

PeakTable

Detector A Ch1 214nm

Peak#	Ret. Time	Area	Height	Area %	Height %
1	4.676	76399	14387	0.139	0.671
2	5.049	1216919	204700	2.210	9.553
3	5.415	53775016	1923777	97.651	89.776
Total		55068333	2142864	100.000	100.000

生工生物工程（上海）股份有限公司

地址: 上海市松江区香闵路698号
电话/Tel: 400-821-0268
邮箱/Email: sales@sangon.com

Add: 698 Xiang Min Road SongJiang Shanghai China
传真/Fax: 86-21-37772170
网址/Web: www.sangon.com

Sample Information

Name	:	P20955-11（GK-(biotin-labeled) peptide）
Sequence	:	GKPVKAR
Modification	:	K-Biotin
Lot.No	:	P20955-11（GK-(biotin-labeled) peptide）
Pump A	:	0.1%trifluoroacetic in 100%water
Pump B	:	0.1%trifluoroacetic in 100%acetonrtrile
Total Flow	:	1.0ml/min
Wavelength	:	214nm
Analytical column type	:	SHIMADZU Inertsil ODS-SP(4.6*150MM*5UM)
Dissolution method	:	0.5mg sample dissolved to 0.5mL by 100%H2O
Inj.Volume	:	60ul

Time	Module	Action	Value
0.01	Pumps	B.Conc	14
20.00	Pumps	B.Conc	34

Chromatogram

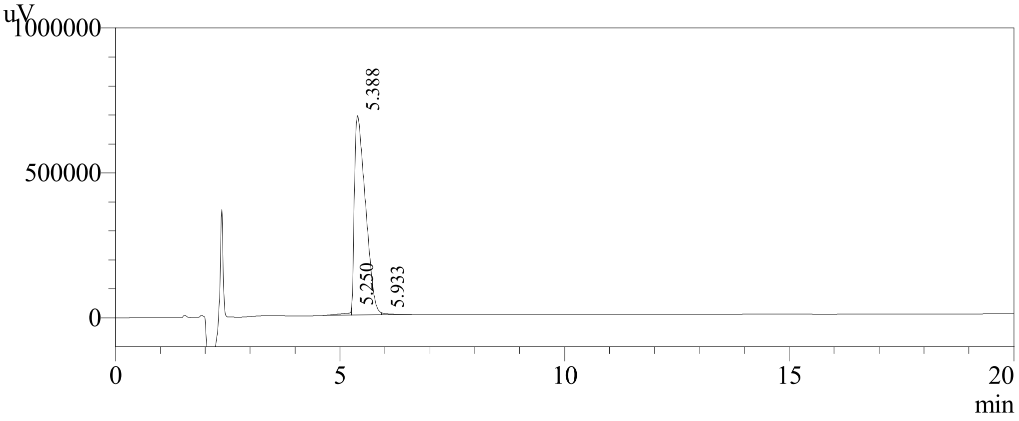

1 Det.A Ch1 / 214nm

PeakTable

Detector A Ch1 214nm

Peak#	Ret. Time	Area	Height	Area %	Height %
1	5.250	159678	15883	1.330	2.235
2	5.388	11783795	688295	98.183	96.872
3	5.933	58348	6341	0.486	0.892
Total		12001821	710520	100.000	100.000

生工生物工程（上海）股份有限公司

地址: 上海市松江区香闵路698号
电话/Tel: 400-821-0268
邮箱/Email: sales@sangon.com

Add: 698 Xiang Min Road SongJiang Shanghai China
传真/Fax: 86-21-37772170
网址/Web: www.sangon.com

生工® Sangon Biotech

Sample Information

Name	:	P20955-12（AGL-(biotin-labeled peptide)）
Sequence	:	AGLPVKAR
Modification	:	K-Biotin
Lot.No	:	P20955-12（AGL-(biotin-labeled peptide)）
Pump A	:	0.1%trifluoroacetic in 100%water
Pump B	:	0.1%trifluoroacetic in 100%acetonrtrile
Total Flow	:	1.0ml/min
Wavelength	:	214nm
Analytical column type	:	SHIMADZU Inertsil ODS-SP(4.6*150MM*5UM)
Dissolution method	:	0.5mg sample dissolved to 0.5mL by 100%H2O
Inj.Volume	:	60ul

Time	Module	Action	Value
0.01	Pumps	B.Conc	16
20.00	Pumps	B.Conc	36

Chromatogram

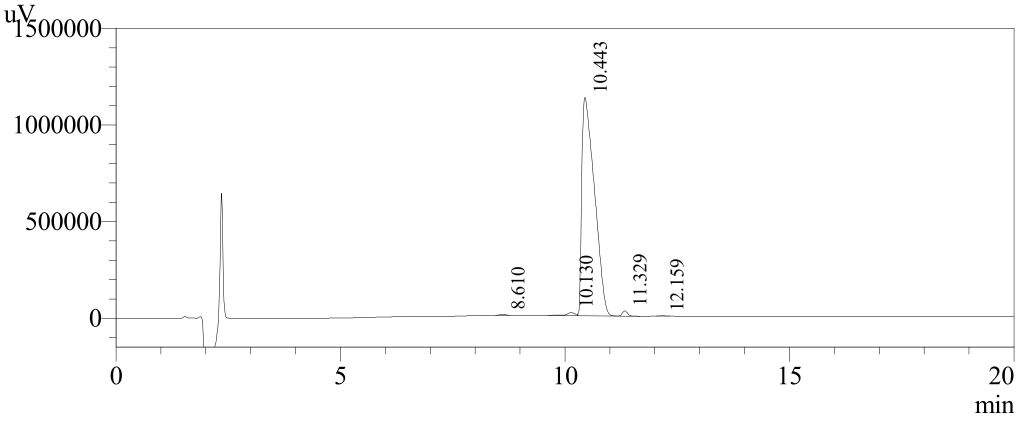

1 Det.A Ch1 / 214nm

PeakTable

Detector A Ch1 214nm

Peak#	Ret. Time	Area	Height	Area %	Height %
1	8.610	44899	4670	0.198	0.395
2	10.130	225297	16221	0.992	1.374
3	10.443	22189408	1130480	97.727	95.741
4	11.329	227474	26854	1.002	2.274
5	12.159	18460	2543	0.081	0.215
Total		22705538	1180768	100.000	100.000

生工生物工程（上海）股份有限公司

地址: 上海市松江区香闵路698号
电话/Tel: 400-821-0268
邮箱/Email: sales@sangon.com

Add: 698 Xiang Min Road SongJiang Shanghai China
传真/Fax: 86-21-37772170
网址/Web: www.sangon.com

Sample Information

Name	:	P20955-13（QGL-(biotin-labeled peptide)）
Sequence	:	QGLPVKAR
Modification	:	K-Biotin
Lot.No	:	P20955-13（QGL-(biotin-labeled peptide)）
Pump A	:	0.1%trifluoroacetic in 100%water
Pump B	:	0.1%trifluoroacetic in 100%acetonrtrile
Total Flow	:	1.0ml/min
Wavelength	:	214nm
Analytical column type	:	SHIMADZU Inertsil ODS-SP(4.6*150MM*5UM)
Dissolution method	:	0.5mg sample dissolved to 0.5mL by 100%H2O
Inj.Volume	:	60ul

Time	Module	Action	Value
0.01	Pumps	B.Conc	18
20.00	Pumps	B.Conc	38

Chromatogram

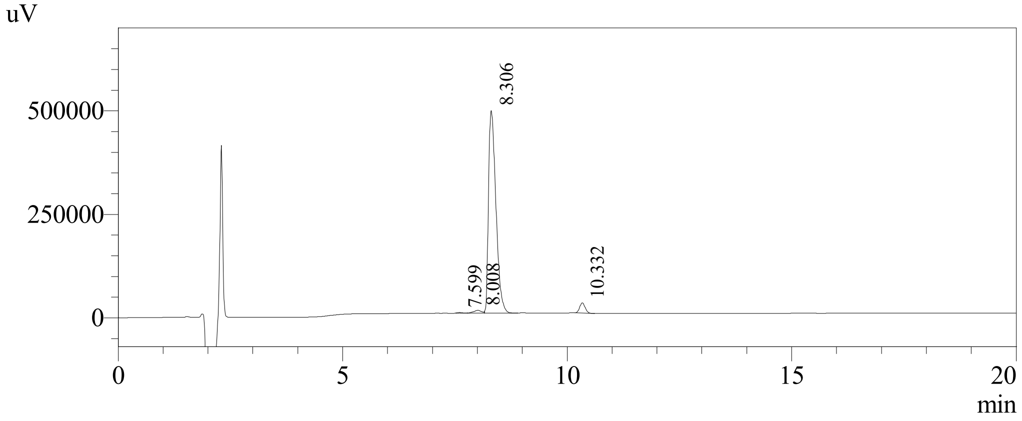

1 Det.A Ch1 / 214nm

PeakTable

Detector A Ch1 214nm

Peak#	Ret. Time	Area	Height	Area %	Height %
1	7.599	8756	1317	0.148	0.252
2	8.008	84926	6823	1.435	1.307
3	8.306	5610878	489316	94.840	93.722
4	10.332	211587	24635	3.576	4.719
Total		5916147	522092	100.000	100.000

生工生物工程（上海）股份有限公司

地址: 上海市松江区香闵路698号
电话/Tel: 400-821-0268
邮箱/Email: sales@sangon.com

Add: 698 Xiang Min Road SongJiang Shanghai China
传真/Fax: 86-21-37772170
网址/Web: www.sangon.com

生工® Sangon Biotech

Sample Information

Name	:	P20955-14（FGL-(biotin-labeled peptide) ）
Sequence	:	FGLPVKAR
Modification	:	K-Biotin
Lot.No	:	P20955-14（FGL-(biotin-labeled peptide) ）
Pump A	:	0.1%trifluoroacetic in 100%water
Pump B	:	0.1%trifluoroacetic in 100%acetonrtrile
Total Flow	:	1.0ml/min
Wavelength	:	214nm
Analytical column type	:	SHIMADZU Inertsil ODS-SP(4.6*150MM*5UM)
Dissolution method	:	0.5mg sample dissolved to 0.5mL by 100%H2O
Inj.Volume	:	60ul

Time	Module	Action	Value
0.01	Pumps	B.Conc	22
20.00	Pumps	B.Conc	42

Chromatogram

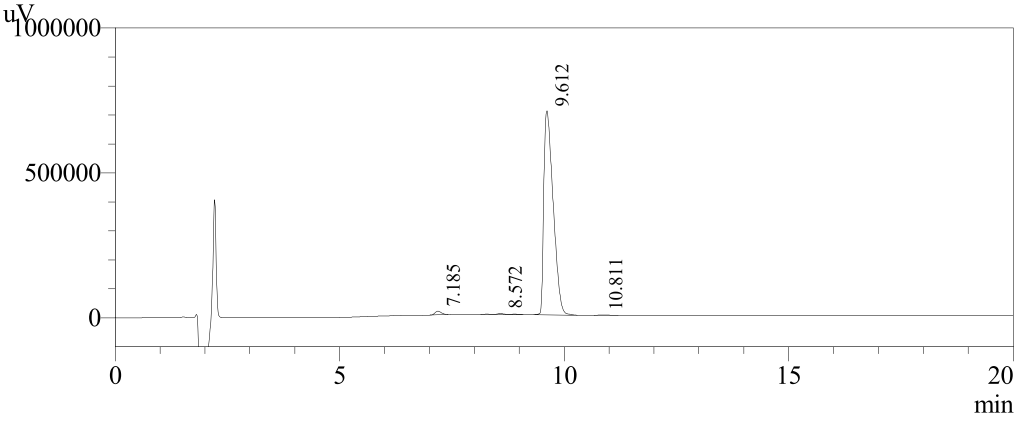

1 Det.A Ch1 / 214nm

PeakTable

Detector A Ch1 214nm

Peak#	Ret. Time	Area	Height	Area %	Height %
1	7.185	125592	12653	1.205	1.755
2	8.572	57894	3462	0.555	0.480
3	9.612	10227545	703926	98.094	97.643
4	10.811	15266	880	0.146	0.122
Total		10426297	720920	100.000	100.000

生工生物工程（上海）股份有限公司

地址: 上海市松江区香闵路698号
电话/Tel: 400-821-0268
邮箱/Email: sales@sangon.com

Add: 698 Xiang Min Road SongJiang Shanghai China
传真/Fax: 86-21-37772170
网址/Web: www.sangon.com

生工® Sangon Biotech

Sample Information

Name	:	P20955-15（DGL-(biotin-labeled peptide)）
Sequence	:	DGLPVKAR
Modification	:	K-Biotin
Lot.No	:	P20955-15（DGL-(biotin-labeled peptide)）
Pump A	:	0.1%trifluoroacetic in 100%water
Pump B	:	0.1%trifluoroacetic in 100%acetonrtrile
Total Flow	:	1.0ml/min
Wavelength	:	214nm
Analytical column type	:	SHIMADZU Inertsil ODS-SP(4.6*150MM*5UM)
Dissolution method	:	0.5mg sample dissolved to 0.5mL by 100%H2O
Inj.Volume	:	60ul

Time	Module	Action	Value
0.01	Pumps	B.Conc	18
20.00	Pumps	B.Conc	38

Chromatogram

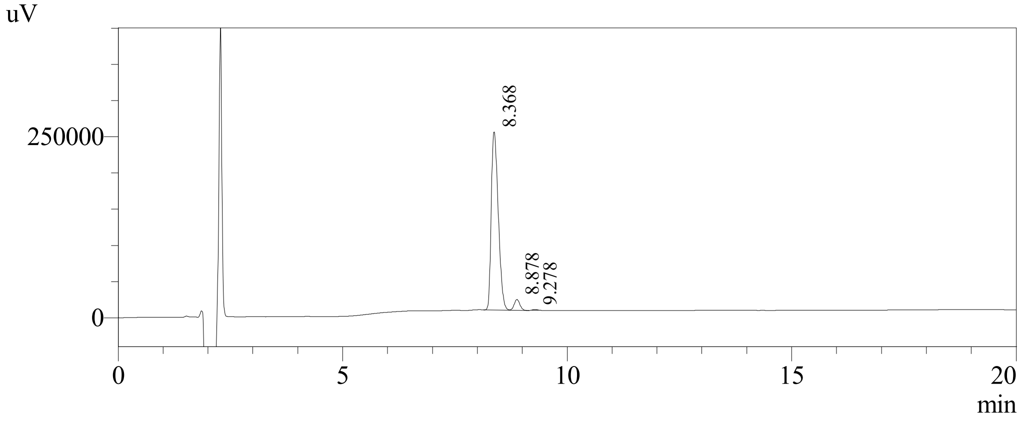

1 Det.A Ch1 / 214nm

PeakTable

Detector A Ch1 214nm

Peak#	Ret. Time	Area	Height	Area %	Height %
1	8.368	2662864	246023	95.104	93.871
2	8.878	129943	14985	4.641	5.718
3	9.278	7142	1079	0.255	0.412
Total		2799949	262087	100.000	100.000

生工生物工程（上海）股份有限公司

地址: 上海市松江区香闵路698号
电话/Tel: 400-821-0268
邮箱/Email: sales@sangon.com
Add: 698 Xiang Min Road SongJiang Shanghai China
传真/Fax: 86-21-37772170
网址/Web: www.sangon.com

Sample Information

Name	:	P20955-16（KGL-(biotin-labeled peptide)）
Sequence	:	KGLPVKAR
Modification	:	K-Biotin
Lot.No	:	P20955-16（KGL-(biotin-labeled peptide)）
Pump A	:	0.1%trifluoroacetic in 100%water
Pump B	:	0.1%trifluoroacetic in 100%acetonrtrile
Total Flow	:	1.0ml/min
Wavelength	:	214nm
Analytical column type	:	SHIMADZU Inertsil ODS-SP(4.6*150MM*5UM)
Dissolution method	:	0.5mg sample dissolved to 0.5mL by 100%H2O
Inj.Volume	:	60ul

Time	Module	Action	Value
0.01	Pumps	B.Conc	16
20.00	Pumps	B.Conc	36

Chromatogram

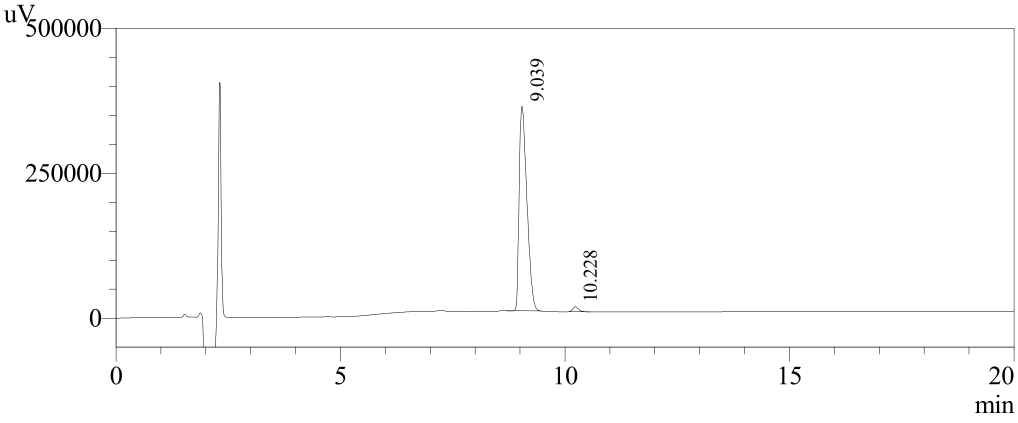

1 Det.A Ch1 / 214nm

PeakTable

Detector A Ch1 214nm

Peak#	Ret. Time	Area	Height	Area %	Height %
1	9.039	4247306	353400	98.274	97.704
2	10.228	74583	8306	1.726	2.296
Total		4321890	361706	100.000	100.000

生工生物工程（上海）股份有限公司

地址: 上海市松江区香闵路698号
电话/Tel: 400-821-0268
邮箱/Email: sales@sangon.com

Add: 698 Xiang Min Road SongJiang Shanghai China
传真/Fax: 86-21-37772170
网址/Web: www.sangon.com

生工® Sangon Biotech

Sample Information

Name	:	P21421-2-HL-(biotin labeled)peptide
Sequence	:	HLPVK(Biotin)AR
Modification	:	K(Biotin)
Lot.No	:	P21421-2-HL-(biotin labeled)peptide
Pump A	:	0.1%trifluoroacetic in 100%water
Pump B	:	0.1%trifluoroacetic in 100%acetonrtrile
Total Flow	:	1.0ml/min
Wavelength	:	214nm
Analytical column type	:	SHIMADZU Inertsil ODS-SP(4.6*250MM*5UM)
Dissolution method	:	0.5mg sample dissolved to 0.5mL by 100%H2O
Inj.Volume	:	60ul

Time	Module	Action	Value
0.01	Pumps	B.Conc	18
20.00	Pumps	B.Conc	38

Chromatogram

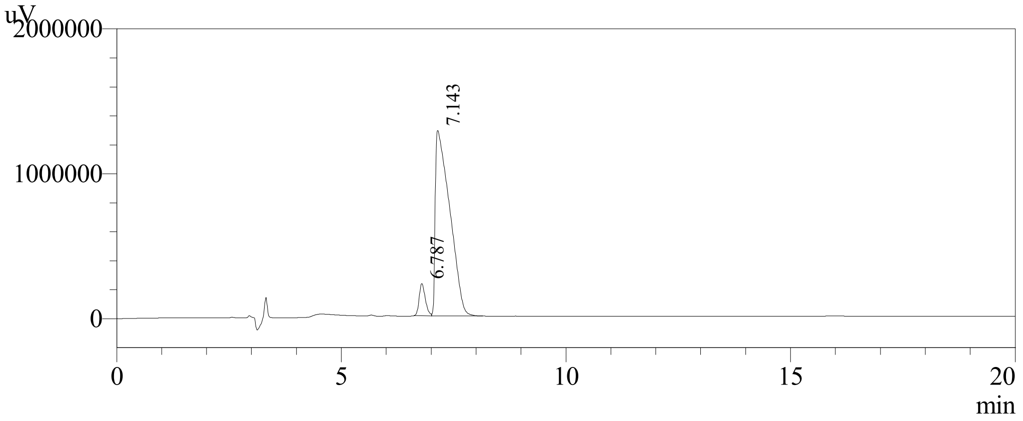

PeakTable

Detector A Ch1 214nm

Peak#	Ret. Time	Area	Height	Area %	Height %
1	6.787	2115828	222415	6.868	14.810
2	7.143	28691478	1279386	93.132	85.190
Total		30807306	1501801	100.000	100.000

生工生物工程（上海）股份有限公司

地址: 上海市松江区香闵路698号
电话/Tel: 400-821-0268
邮箱/Email: sales@sangon.com

Add: 698 Xiang Min Road SongJiang Shanghai China
传真/Fax: 86-21-37772170
网址/Web: www.sangon.com

生工® Sangon Biotech

Sample Information

Name : P21421-1-RL-(biotin labeled)peptide
Sequence : RLPVK(Biotin)AR
Modification : K(Biotin)
Lot.No : P21421-1-RL-(biotin labeled)peptide
Pump A : 0.1%trifluoroacetic in 100%water
Pump B : 0.1%trifluoroacetic in 100%acetonrtrile
Total Flow : 1.0ml/min
Wavelength : 214nm
Analytical column type : SHIMADZU Inertsil ODS-SP(4.6*250MM*5UM)
Dissolution method : 0.5mg sample dissolved to 0.5mL by 100%H2O
Inj.Volume : 60ul

Time	Module	Action	Value
0.01	Pumps	B.Conc	16
20.00	Pumps	B.Conc	36

Chromatogram

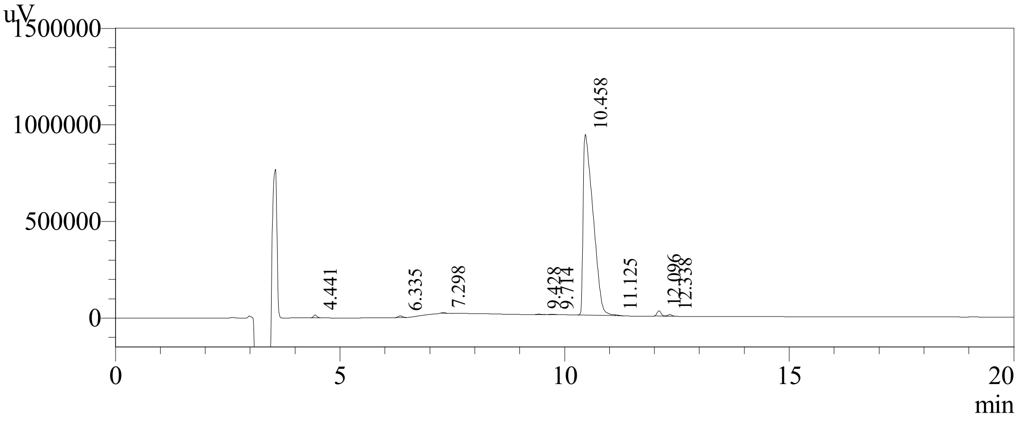

1 Det.A Ch1 / 214nm

PeakTable

Detector A Ch1 214nm

Peak#	Ret. Time	Area	Height	Area %	Height %
1	4.441	74090	14226	0.501	1.416
2	6.335	56013	8532	0.379	0.849
3	7.298	11109	2333	0.075	0.232
4	9.428	15088	2608	0.102	0.260
5	9.714	15058	2448	0.102	0.244
6	10.458	14364974	934957	97.216	93.060
7	11.125	20550	4319	0.139	0.430
8	12.096	165757	26916	1.122	2.679
9	12.338	53728	8344	0.364	0.830
Total		14776368	1004682	100.000	100.000

生工生物工程（上海）股份有限公司

地址: 上海市松江区香闵路698号
电话/Tel: 400-821-0268
邮箱/Email: sales@sangon.com

Add: 698 Xiang Min Road SongJiang Shanghai China
传真/Fax: 86-21-37772170
网址/Web: www.sangon.com

生工® Sangon Biotech

Sample Information

Name	:	P27704-3（RL-(Dabcyl labeled) peptide）
Sequence	:	RLPVK（Dabcyl）AR
Modification	:	K（Dabcyl）
Lot.No	:	P27704-3（RL-(Dabcyl labeled) peptide）
Pump A	:	0.1%trifluoroacetic in 100%water
Pump B	:	0.1%trifluoroacetic in 100%acetonrtrile
Total Flow	:	1.0ml/min
Wavelength	:	214nm
Analytical column type	:	SHIMADZU shim-pack GIST(4.6*250MM*5UM)
Dissolution method	:	0.1mg sample dissolved to 0.5mL by 10%ACN and 90%H2O
Inj.Volume	:	30ul

Time	Module	Action	Value
0.01	Pumps	B.Conc	29
20.00	Pumps	B.Conc	49

Chromatogram

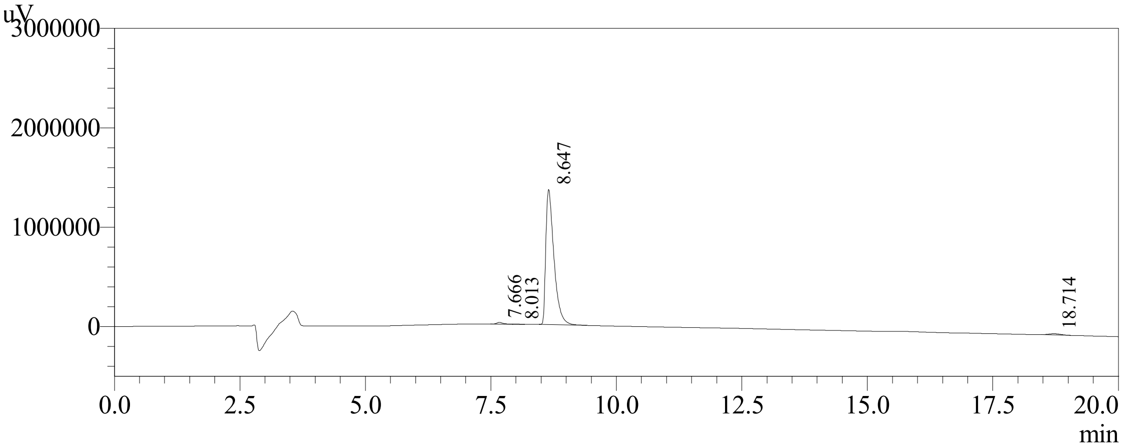

1 Det.A Ch1 / 214nm

PeakTable

Detector A Ch1 214nm

Peak#	Ret. Time	Area	Height	Area %	Height %
1	7.666	156483	17227	0.997	1.240
2	8.013	17992	2428	0.115	0.175
3	8.647	15311317	1357152	97.559	97.660
4	18.714	208700	12861	1.330	0.925
Total		15694491	1389667	100.000	100.000

生工生物工程（上海）股份有限公司

地址: 上海市松江区香闵路698号
电话/Tel: 400-821-0268
邮箱/Email: sales@sangon.com

Add: 698 Xiang Min Road SongJiang Shanghai China
传真/Fax: 86-21-37772170
网址/Web: www.sangon.com

生工® Sangon Biotech

MS Spectrum

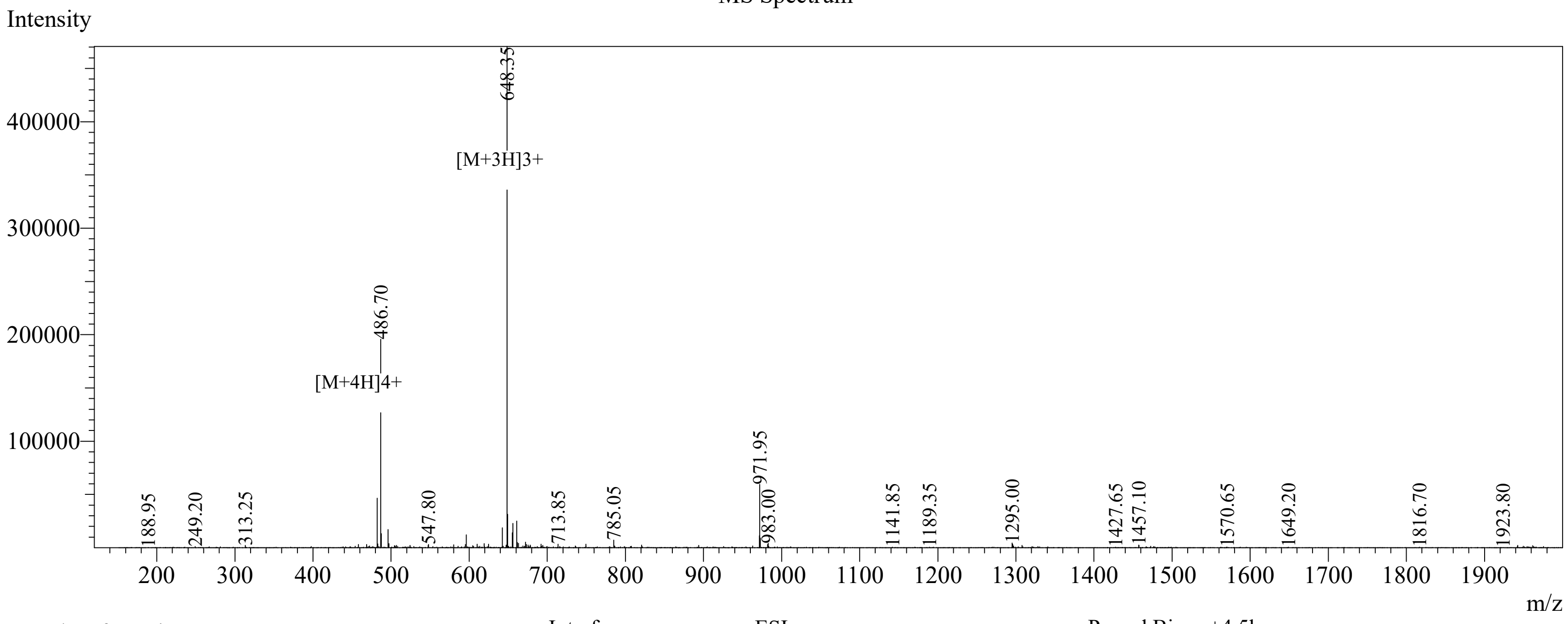

Sample Information
Dissolution method :0.1mg sample dissolved to 0.5mL by 100%H2O
Injection Volume :1ul

Name :P22101(Pep133-NGL)
Sequence :SAAERKHRHLPVADANGL
Modification :N/A
Lot No. :P22101(Pep133-NGL)
Theoretical :1942.153
observed :1942.05

Interface :ESI
Nebulizing Gas Flow :1.50L/min
CDL Temp :250℃
CDL Volt :0v
Block Temp :200

Prerod Bias :+4.5kv
Detector :-0.2kv
T.Flow :0.2ml/min
B.conc :50%H2O/50%MeOH

生工生物工程（上海）股份有限公司

地址：上海市松江区香闵路698号
电话/Tel: 400-821-0268
邮箱/Email: Sales@sangon.com

Add: 698 Xiang Min Road Songjiang Shanghai China
传真/Fax: 86-21-37772170
网址/Web: wwww.sangon.com

MS Spectrum

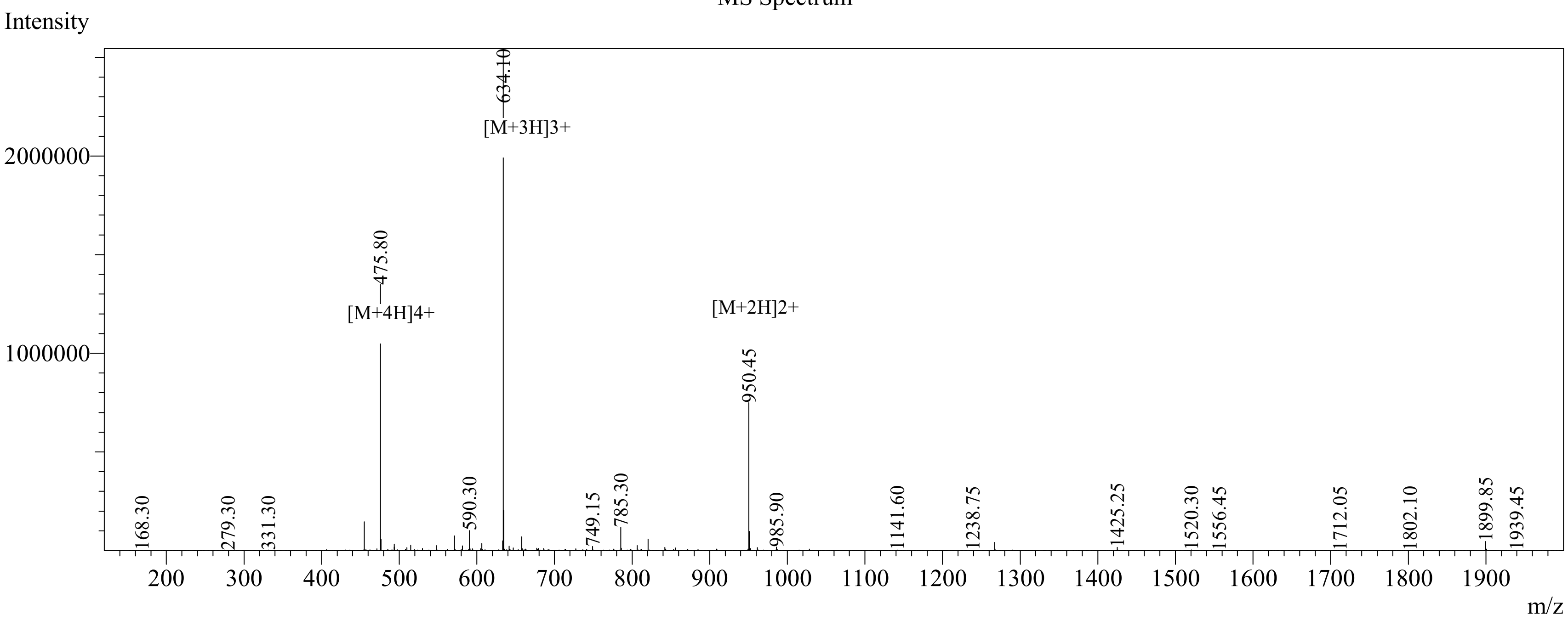

Sample Information
Dissolution method :0.1mg sample dissolved to 0.5mL by 100%H2O
Injection Volume :1ul

Name :P20571-1 (Pep133-AGL)
Sequence :SAAERKHRHLPVADAAGL
Modification :N/A
Lot No. :P20571-1 (Pep133-AGL)
Theoretical :1899.128
observed :1899.3

Interface :ESI
Nebulizing Gas Flow :1.50L/min
CDL Temp :250℃
CDL Volt :0v
Block Temp :200

Prerod Bias :+4.5kv
Detector :-0.2kv
T.Flow :0.2ml/min
B.conc :50%H2O/50%MeOH

生工生物工程（上海）股份有限公司

地址：上海市松江区香闵路698号
电话/Tel: 400-821-0268
邮箱/Email: Sales@sangon.com

Add: 698 Xiang Min Road Songjiang Shanghai China
传真/Fax: 86-21-37772170
网址/Web: wwww.sangon.com

MS Spectrum

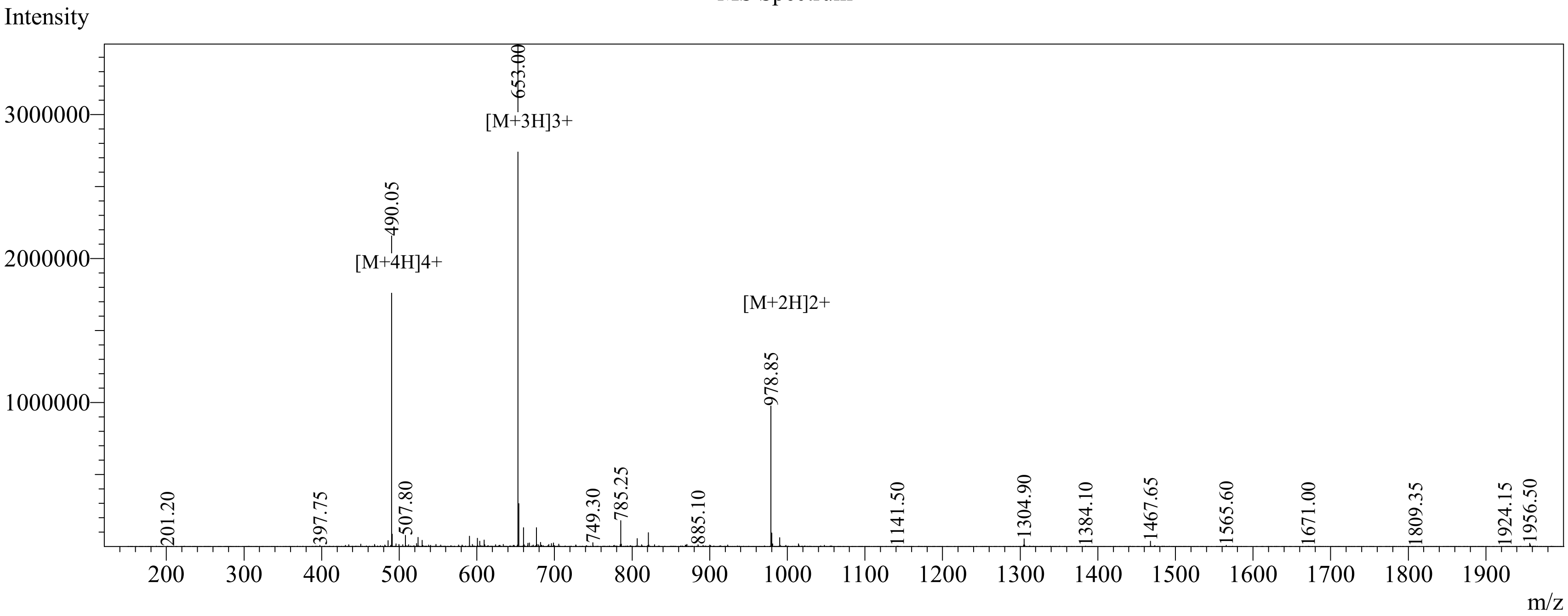

Sample Information
Dissolution method :0.1mg sample dissolved to 0.5mL by 100%H2O
Injection Volume :1ul

Interface :ESI
Nebulizing Gas Flow :1.50L/min
CDL Temp :250℃
CDL Volt :0v
Block Temp :200

Prerod Bias :+4.5kv
Detector :-0.2kv
T.Flow :0.2ml/min
B.conc :50%H2O/50%MeOH

Name :P20571-2 (Pep133-QGL)
Sequence :SAAERKHRHLPVADAQGL
Modification :N/A
Lot No. :P20571-2 (Pep133-QGL)
Theoretical :1956.18
observed :1956.0

生工生物工程（上海）股份有限公司

地址：上海市松江区香闵路698号
Add: 698 Xiang Min Road Songjiang Shanghai China
电话/Tel: 400-821-0268
传真/Fax: 86-21-37772170
邮箱/Email: Sales@sangon.com
网址/Web: wwww.sangon.com

MS Spectrum

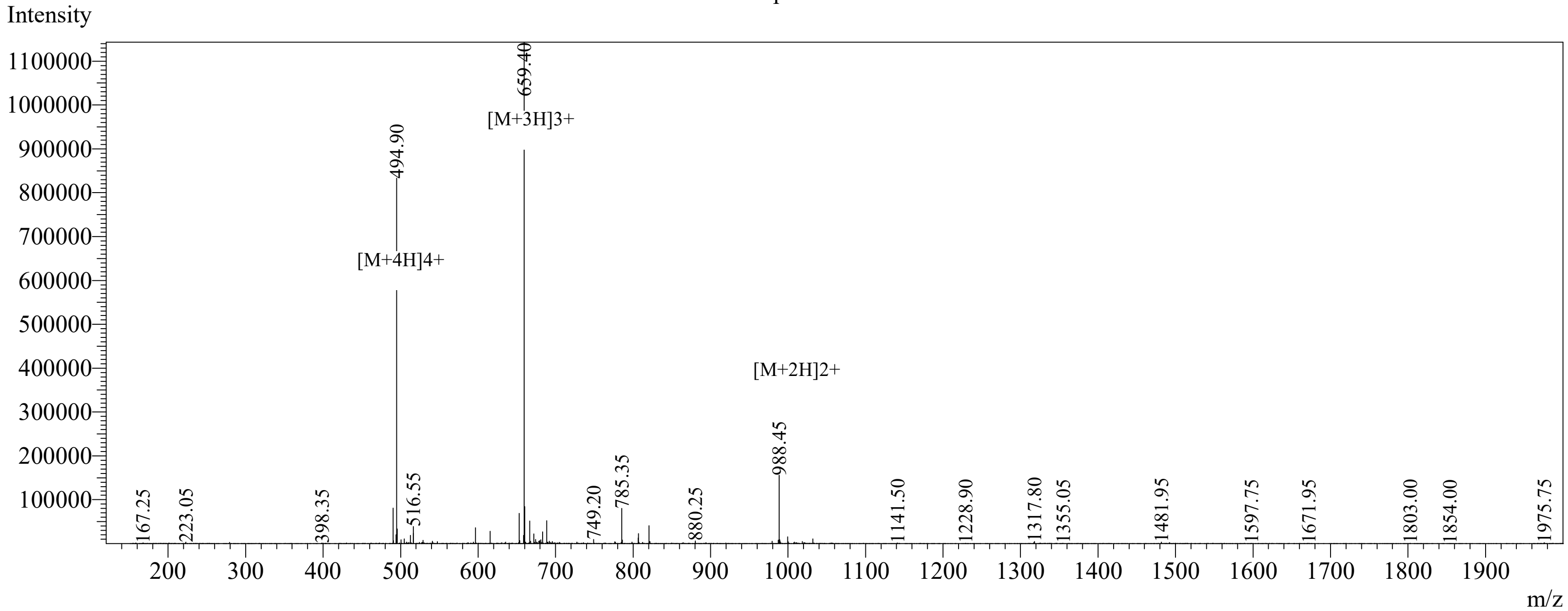

Sample Information
Dissolution method :0.1mg sample dissolved to 0.5mL by 100%H2O
Injection Volume :1ul

Name :P20571-3(Pep133-FGL)
Sequence :SAAERKHRHLPVADAFGL
Modification :N/A
Lot No. :P20571-3(Pep133-FGL)
Theoretical :1975.226
observed :1975.20

Interface :ESI
Nebulizing Gas Flow :1.50L/min
CDL Temp :250°C
CDL Volt :0v
Block Temp :200

Prerod Bias :+4.5kv
Detector :-0.2kv
T.Flow :0.2ml/min
B.conc :50%H2O/50%MeOH

生工生物工程（上海）股份有限公司

地址：上海市松江区香闵路698号
电话/Tel: 400-821-0268
邮箱/Email: Sales@sangon.com

Add: 698 Xiang Min Road Songjiang Shanghai China
传真/Fax: 86-21-37772170
网址/Web: wwww.sangon.com

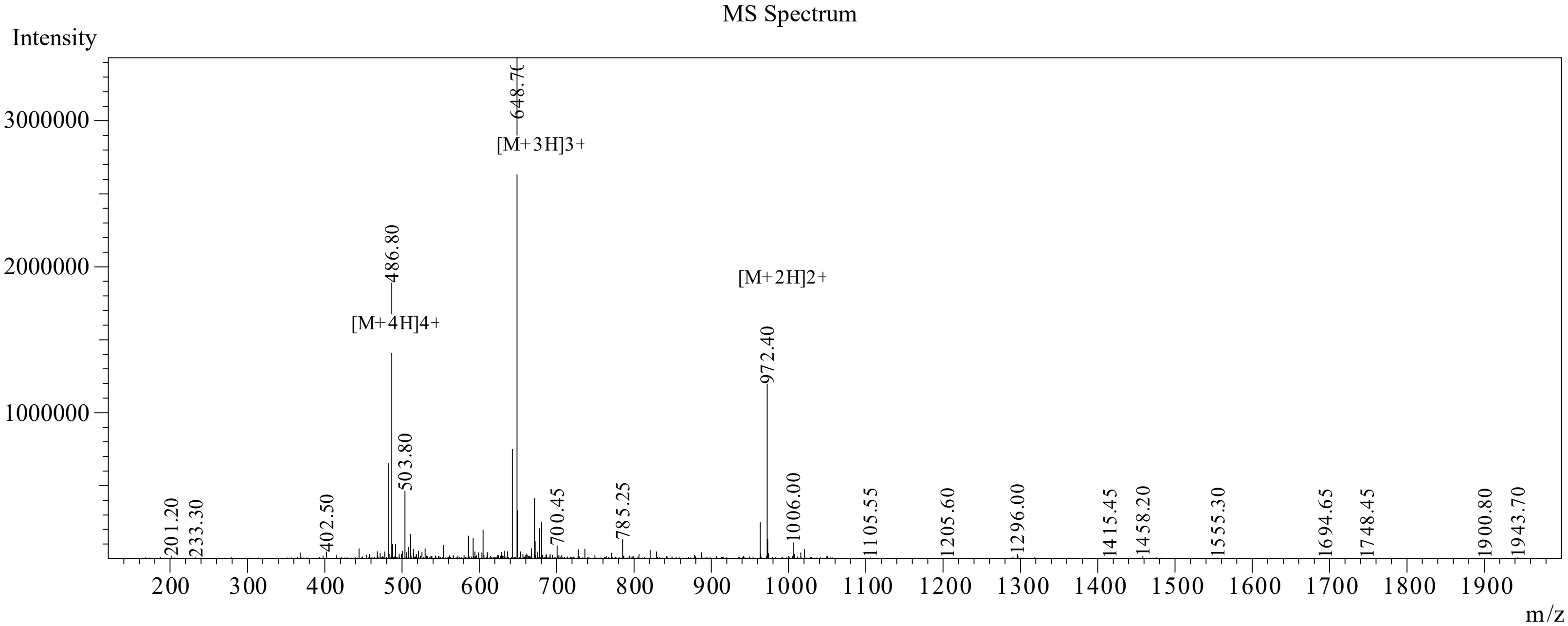

Sample Information
Dissolution method :0.1mg sample dissolved to 0.5mL by 100%H2O
Injection Volume :1ul

Name :P20571-4 (Pep133-DGL)
Sequence :SAAERKHRHLPVADADGL
Modification :N/A
Lot No. :P20571-4 (Pep133-DGL)
Theoretical :1943.13
observed :1943.1

Interface :ESI
Nebulizing Gas Flow :1.50L/min
CDL Temp :250℃
CDL Volt :0v
Block Temp :200

Prerod Bias :+4.5kv
Detector :-0.2kv
T.Flow :0.2ml/min
B.conc :50%H2O/50%MeOH

生工生物工程（上海）股份有限公司

地址：上海市松江区香闵路698号
电话/Tel: 400-821-0268
邮箱/Email: Sales@sangon.com

Add: 698 Xiang Min Road Songjiang Shanghai China
传真/Fax: 86-21-37772170
网址/Web: wwww.sangon.com

MS Spectrum

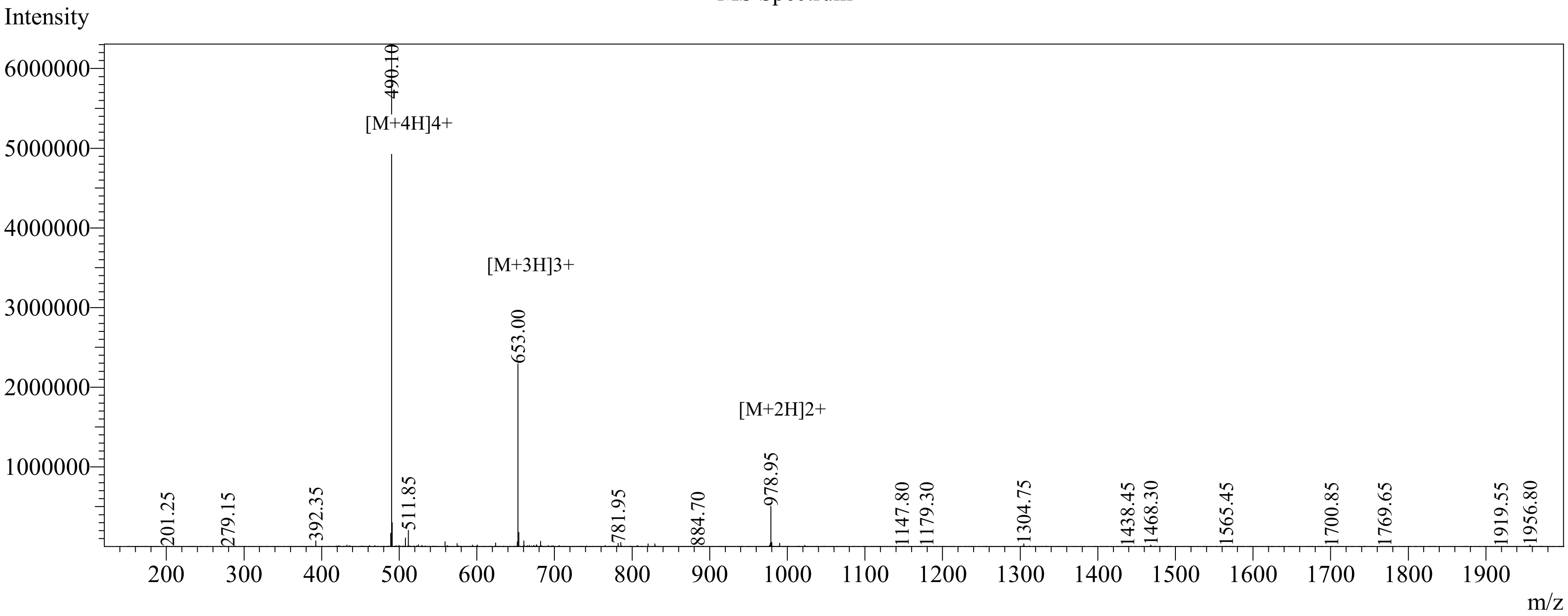

Sample Information
Dissolution method :0.1mg sample dissolved to 0.5mL by 100%H2O
Injection Volume :1ul
Name :P20571-5 (Pep133-KGL)
Sequence :SAAERKHRHLPVADAKGL
Modification :N/A
Lot No. :P20571-5 (Pep133-KGL)
Theoretical :1956.219
bserved :1956.40

Interface :ESI
Nebulizing Gas Flow :1.50L/min
CDL Temp :250℃
CDL Volt :0v
Block Temp :200

Prerod Bias :+4.5kv
Detector :-0.2kv
T.Flow :0.2ml/min
B.conc :50%H2O/50%MeOH

生工生物工程（上海）股份有限公司

地址：上海市松江区香闵路698号
电话/Tel: 400-821-0268
邮箱/Email: Sales@sangon.com

Add: 698 Xiang Min Road Songjiang Shanghai China
传真/Fax: 86-21-37772170
网址/Web: wwww.sangon.com

MS Spectrum

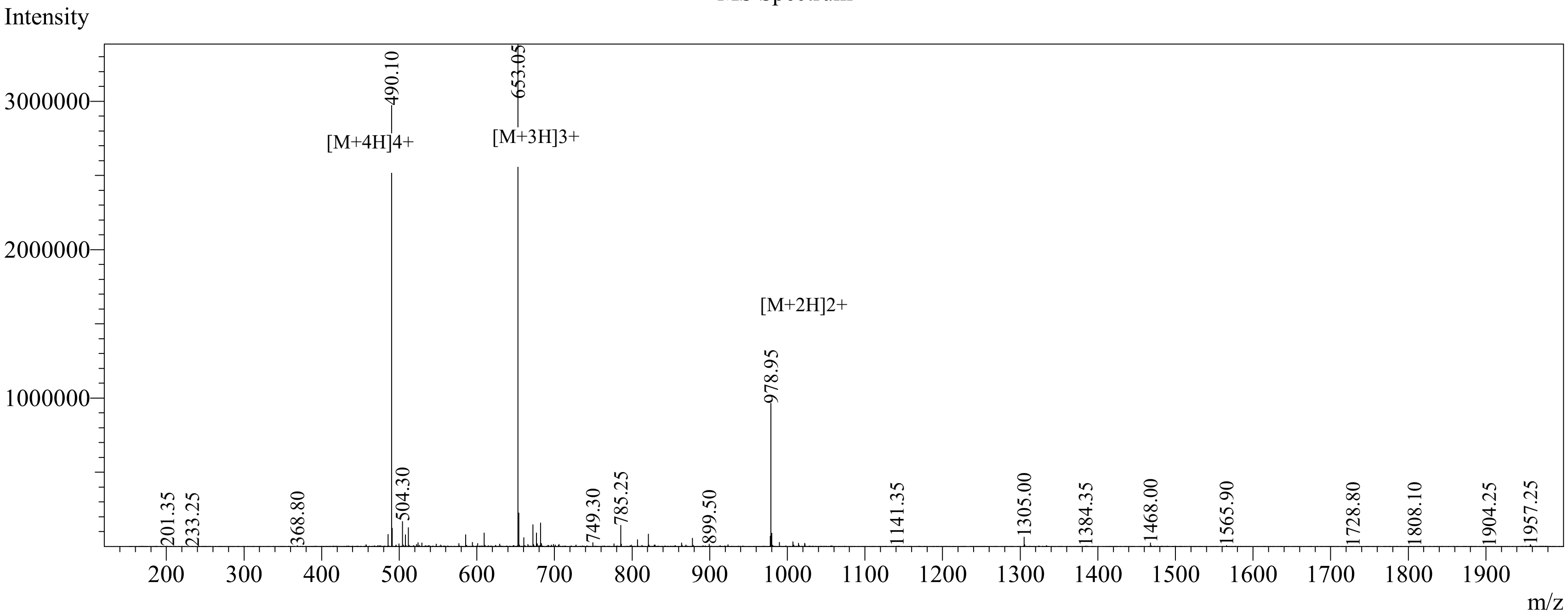

Sample Information
Dissolution method :0.1mg sample dissolved to 0.5mL by 100%H2O
Injection Volume :1ul

Name :P20571-6 (Pep133-NAL)
Sequence :SAAERKHRHLPVADANAL
Modification :N/A
Lot No. :P20571-6 (Pep133-NAL)
Theoretical :1956.18
observed :1956.15

Interface :ESI
Nebulizing Gas Flow :1.50L/min
CDL Temp :250℃
CDL Volt :0v
Block Temp :200

Prerod Bias :+4.5kv
Detector :-0.2kv
T.Flow :0.2ml/min
B.conc :50%H2O/50%MeOH

生工生物工程（上海）股份有限公司

地址：上海市松江区香闵路698号
电话/Tel: 400-821-0268
邮箱/Email: Sales@sangon.com

Add: 698 Xiang Min Road Songjiang Shanghai China
传真/Fax: 86-21-37772170
网址/Web: wwww.sangon.com

生工® Sangon Biotech

MS Spectrum

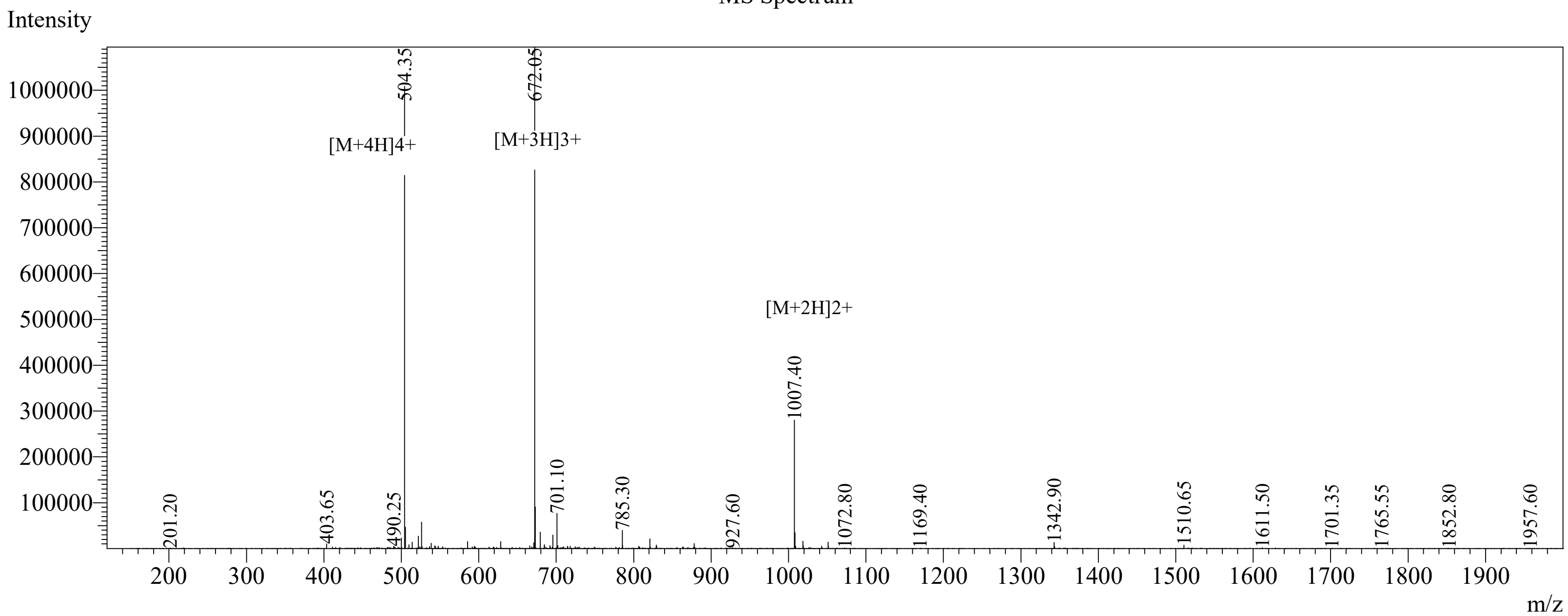

Sample Information
Dissolution method :0.1mg sample dissolved to 0.5mL by 100%H2O
Injection Volume :1ul

Name :P20571-7 (Pep133-NQL)
Sequence :SAAERKHRHLPVADANQL
Modification :N/A
Lot No. :P20571-7 (Pep133-NQL)
Theoretical :2013.232
bserved :2013.15

Interface :ESI
Nebulizing Gas Flow :1.50L/min
CDL Temp :250°C
CDL Volt :0v
Block Temp :200

Prerod Bias :+4.5kv
Detector :-0.2kv
T.Flow :0.2ml/min
B.conc :50%H2O/50%MeOH

生工生物工程（上海）股份有限公司

地址：上海市松江区香闵路698号
电话/Tel: 400-821-0268
邮箱/Email: Sales@sangon.com

Add: 698 Xiang Min Road Songjiang Shanghai China
传真/Fax: 86-21-37772170
网址/Web: wwww.sangon.com

MS Spectrum

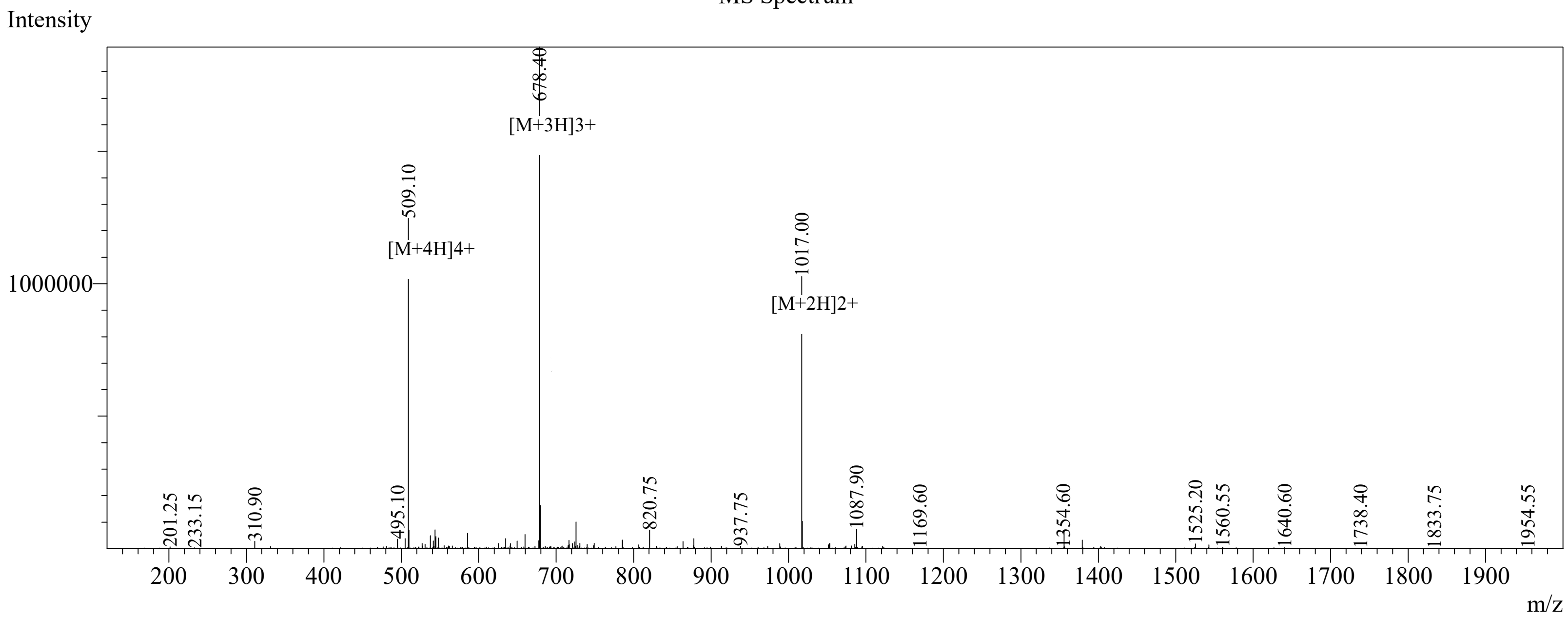

Sample Information
Dissolution method :0.1mg sample dissolved to 0.5mL by 100%H2O
Injection Volume :1ul

Name :P20571-8 (Pep133-NFL)
Sequence :SAAERKHRHLPVADANFL
Modification :N/A
Lot No. :P20571-8 (Pep133-NFL)
Theoretical :2032.278
observed :2032.20

Interface :ESI
Nebulizing Gas Flow :1.50L/min
CDL Temp :250°C
CDL Volt :0v
Block Temp :200

Prerod Bias :+4.5kv
Detector :-0.2kv
T.Flow :0.2ml/min
B.conc :50%H2O/50%MeOH

生工生物工程（上海）股份有限公司

地址：上海市松江区香闵路698号
电话/Tel: 400-821-0268
邮箱/Email: Sales@sangon.com

Add: 698 Xiang Min Road Songjiang Shanghai China
传真/Fax: 86-21-37772170
网址/Web: wwww.sangon.com

MS Spectrum

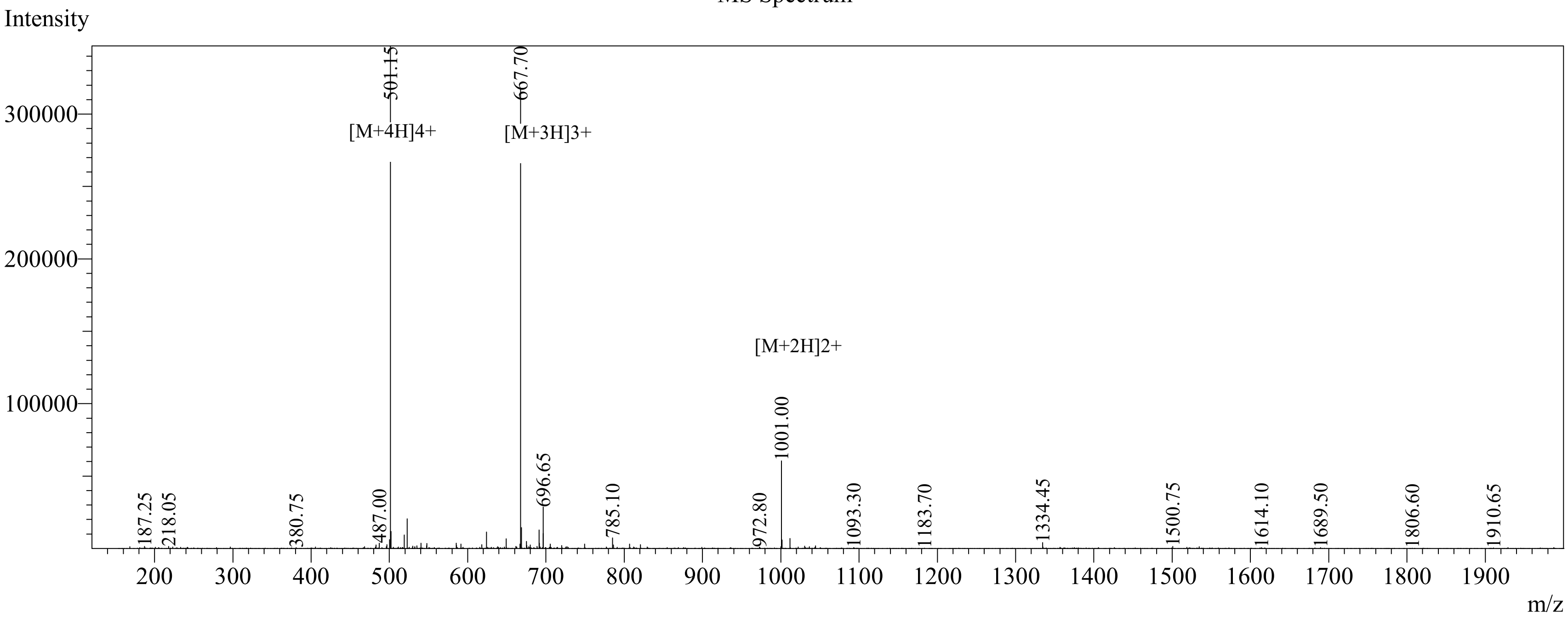

Sample Information
Dissolution method :0.1mg sample dissolved to 0.5mL by 100%H2O
Injection Volume :1ul
Name :P20571-9 (Pep133-NDL)
Sequence :SAAERKHRHLPVADANDL
Modification :N/A
Lot No. :P20571-9 (Pep133-NDL)
Theoretical :2000.19
observed :2000.60

Interface :ESI
Nebulizing Gas Flow :1.50L/min
CDL Temp :250℃
CDL Volt :0v
Block Temp :200

Prerod Bias :+4.5kv
Detector :-0.2kv
T.Flow :0.2ml/min
B.conc :50%H2O/50%MeOH

生工生物工程（上海）股份有限公司

地址：上海市松江区香闵路698号
Add: 698 Xiang Min Road Songjiang Shanghai China
电话/Tel: 400-821-0268
传真/Fax: 86-21-37772170
邮箱/Email: Sales@sangon.com
网址/Web: wwww.sangon.com

生工® Sangon Biotech

MS Spectrum

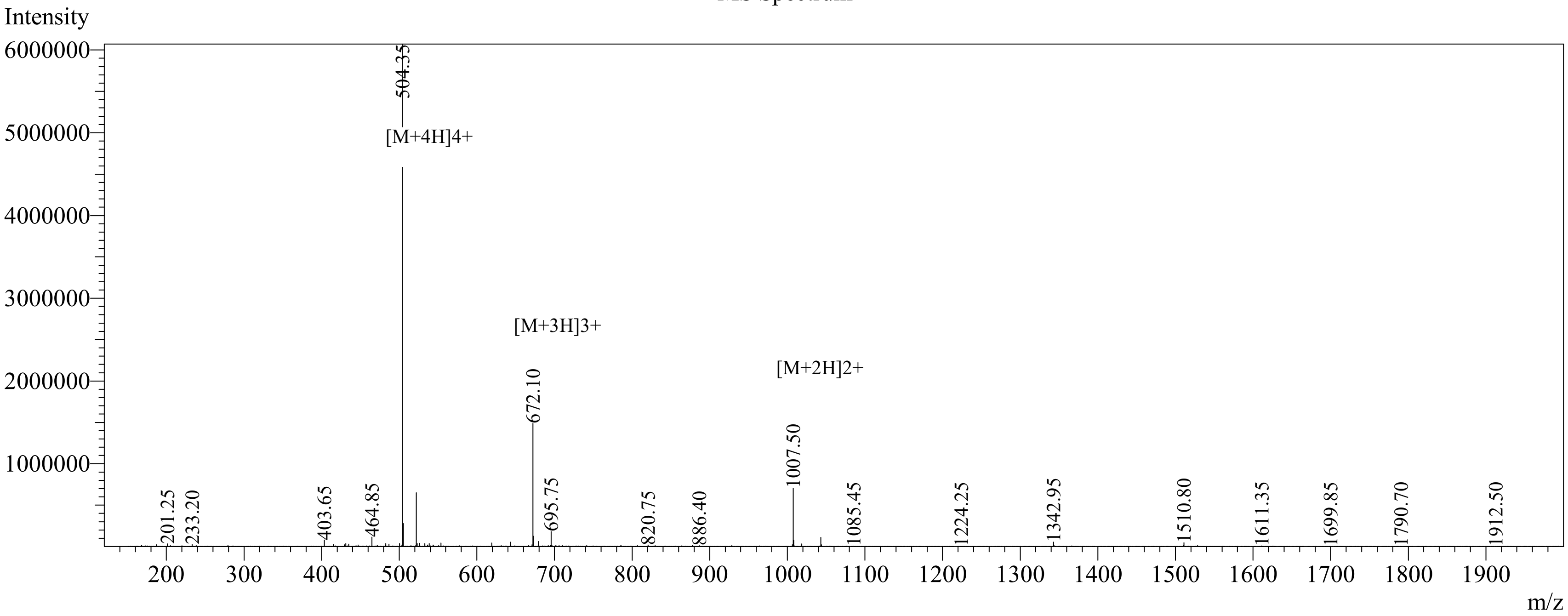

Sample Information
Dissolution method :0.1mg sample dissolved to 0.5mL by 100%H2O
Injection Volume :1ul
Name :P20571-10 (Pep133-NKL)
Sequence :SAAERKHRHLPVADANKL
Modification :N/A
Lot No. :P20571-10 (Pep133-NKL)
Theoretical :2013.271
observed :2013.40

Interface :ESI
Nebulizing Gas Flow :1.50L/min
CDL Temp :250℃
CDL Volt :0v
Block Temp :200

Prerod Bias :+4.5kv
Detector :-0.2kv
T.Flow :0.2ml/min
B.conc :50%H2O/50%MeOH

生工生物工程（上海）股份有限公司

地址：上海市松江区香闵路698号
电话/Tel: 400-821-0268
邮箱/Email: Sales@sangon.com

Add: 698 Xiang Min Road Songjiang Shanghai China
传真/Fax: 86-21-37772170
网址/Web: wwww.sangon.com

生工® Sangon Biotech

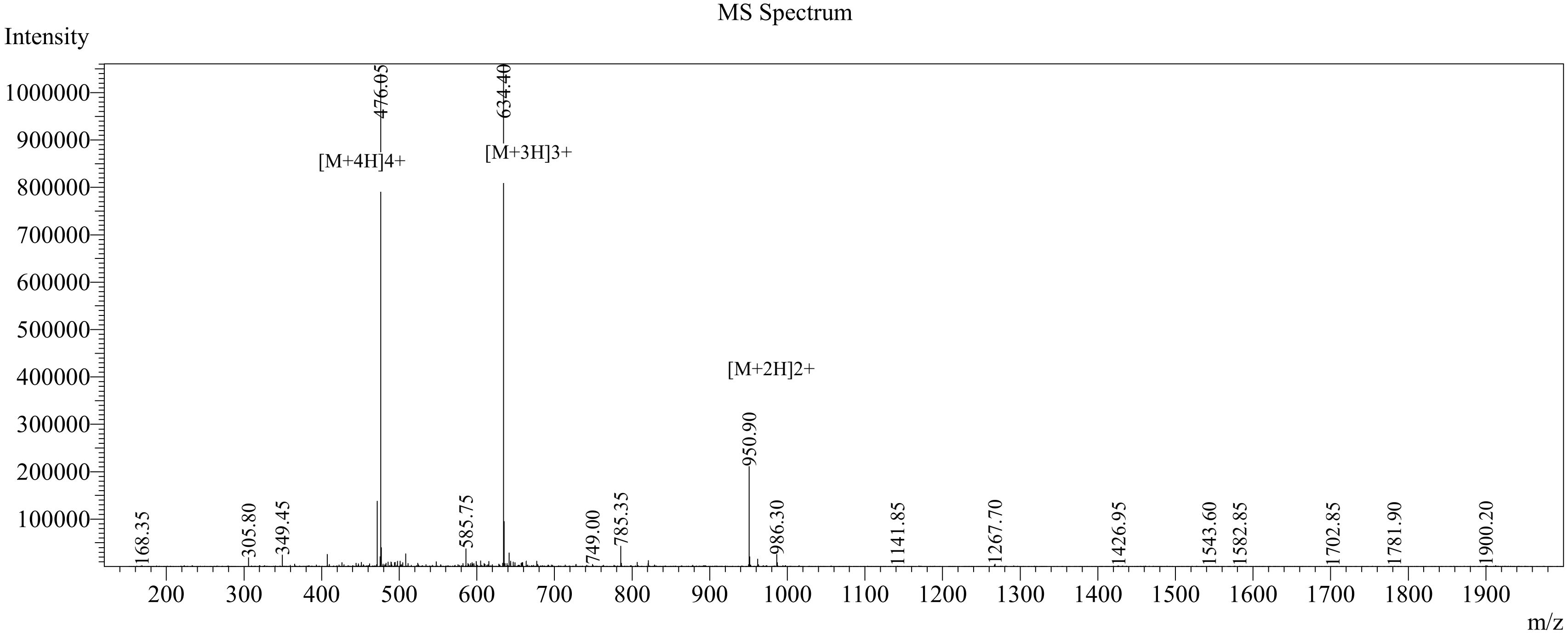

Sample Information
Dissolution method :0.1mg sample dissolved to 0.5mL by 100%H2O
Injection Volume :1ul
Name :P20571-11 (Pep133-NGA)
Sequence :SAAERKHRHLPVADANGA
Modification :N/A
Lot No. :P20571-11 (Pep133-NGA)
Theoretical :1900.072
Observed :1900.20

Interface :ESI
Nebulizing Gas Flow :1.50L/min
CDL Temp :250℃
CDL Volt :0v
Block Temp :200

Prerod Bias :+4.5kv
Detector :-0.2kv
T.Flow :0.2ml/min
B.conc :50%H2O/50%MeOH

生工生物工程（上海）股份有限公司

地址：上海市松江区香闵路698号
电话/Tel: 400-821-0268
邮箱/Email: Sales@sangon.com

Add: 698 Xiang Min Road Songjiang Shanghai China
传真/Fax: 86-21-37772170
网址/Web: wwww.sangon.com

生工® Sangon Biotech

MS Spectrum

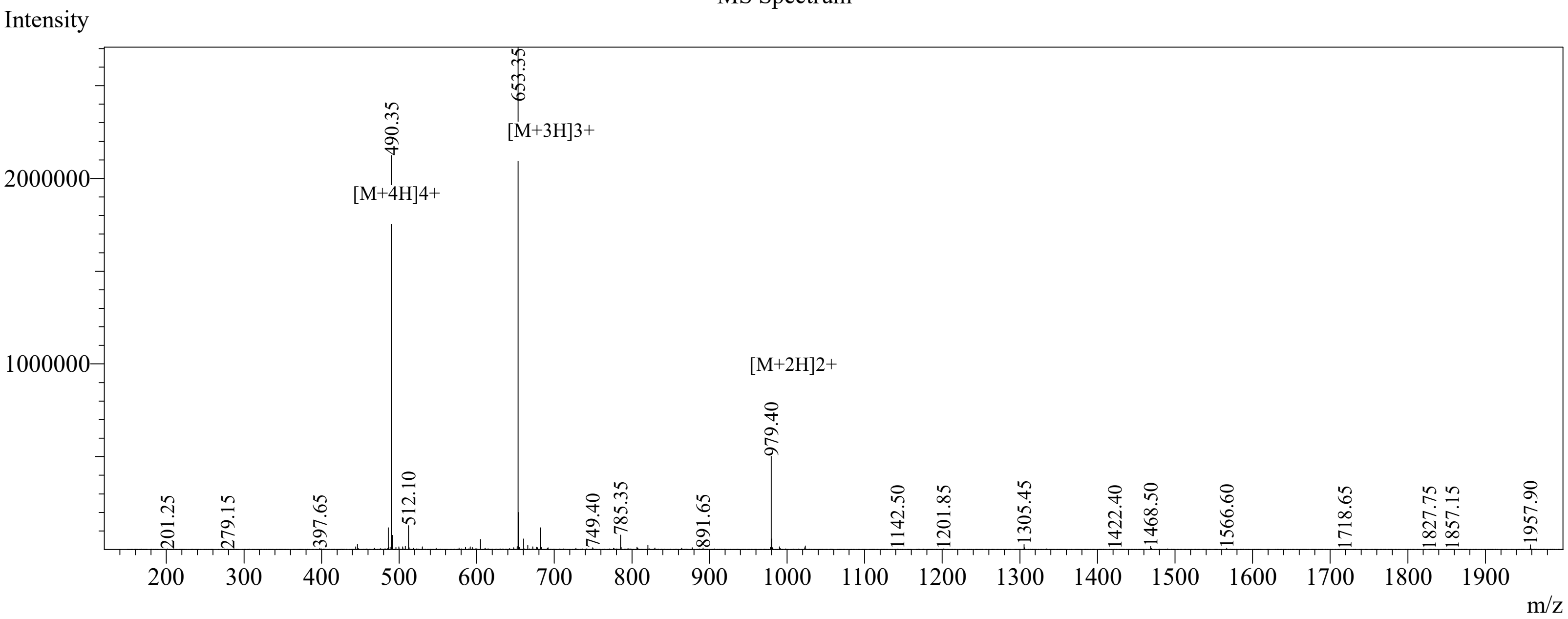

Sample Information
Dissolution method :0.1mg sample dissolved to 0.5mL by 100%H2O
Injection Volume :1ul
Name :P20571-12 (Pep133-NGQ)
Sequence :SAAERKHRHLPVADANGQ
Modification :N/A
Lot No. :P20571-12 (Pep133-NGQ)
Theoretical :1957.124
Observed :1957.05

Interface :ESI
Nebulizing Gas Flow :1.50L/min
CDL Temp :250℃
CDL Volt :0v
Block Temp :200

Prerod Bias :+4.5kv
Detector :-0.2kv
T.Flow :0.2ml/min
B.conc :50%H2O/50%MeOH

生工生物工程（上海）股份有限公司

地址：上海市松江区香闵路698号
Add: 698 Xiang Min Road Songjiang Shanghai China
电话/Tel: 400-821-0268
传真/Fax: 86-21-37772170
邮箱/Email: Sales@sangon.com
网址/Web: wwww.sangon.com

MS Spectrum

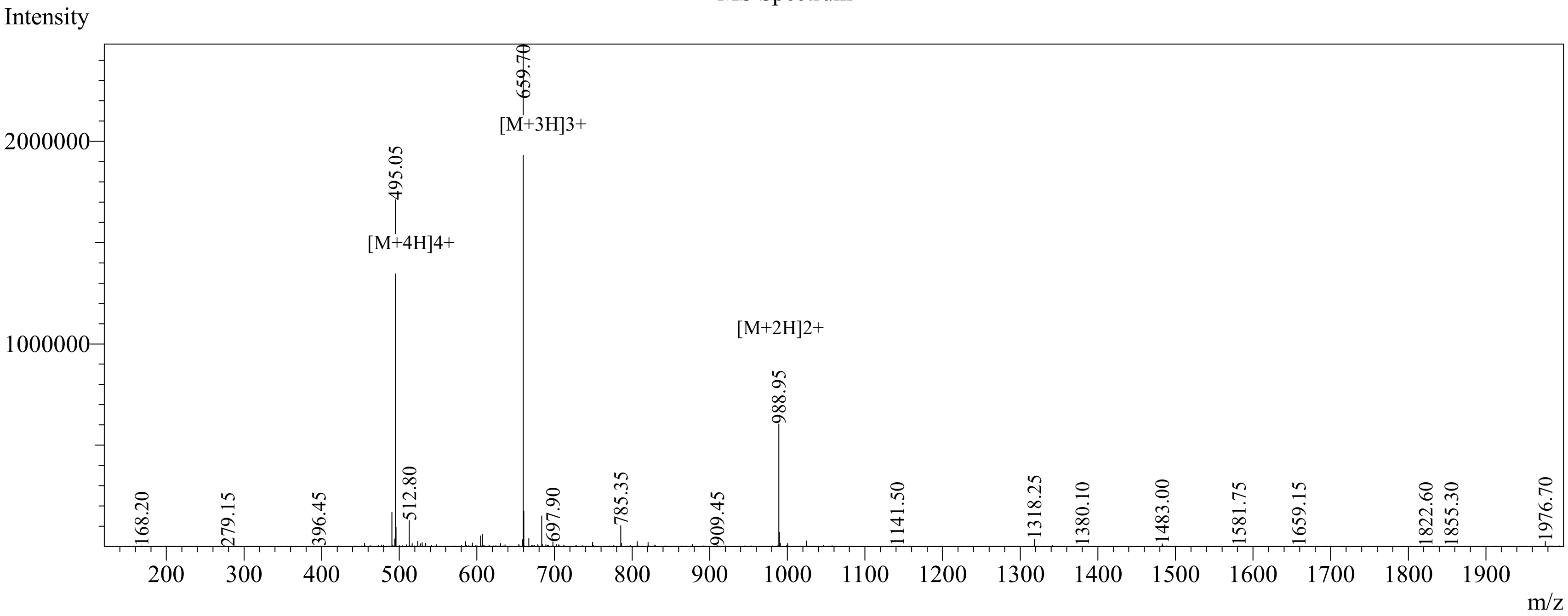

Sample Information
Dissolution method :0.1mg sample dissolved to 0.5mL by 100%H2O
Injection Volume :1ul
Name :P20571-13（Pep133-NGF)
Sequence :SAAERKHRHLPVADANGF
Modification :N/A
Lot No. :P20571-13 (Pep133-NGF)
Theoretical :1976.17
Observed :1976.1

Interface :ESI
Nebulizing Gas Flow :1.50L/min
CDL Temp :250℃
CDL Volt :0v
Block Temp :200

Prerod Bias :+4.5kv
Detector :-0.2kv
T.Flow :0.2ml/min
B.conc :50%H2O/50%MeOH

生工生物工程（上海）股份有限公司

地址：上海市松江区香闵路698号
电话/Tel: 400-821-0268
邮箱/Email: Sales@sangon.com

Add: 698 Xiang Min Road Songjiang Shanghai China
传真/Fax: 86-21-37772170
网址/Web: wwww.sangon.com

生工® Sangon Biotech

MS Spectrum

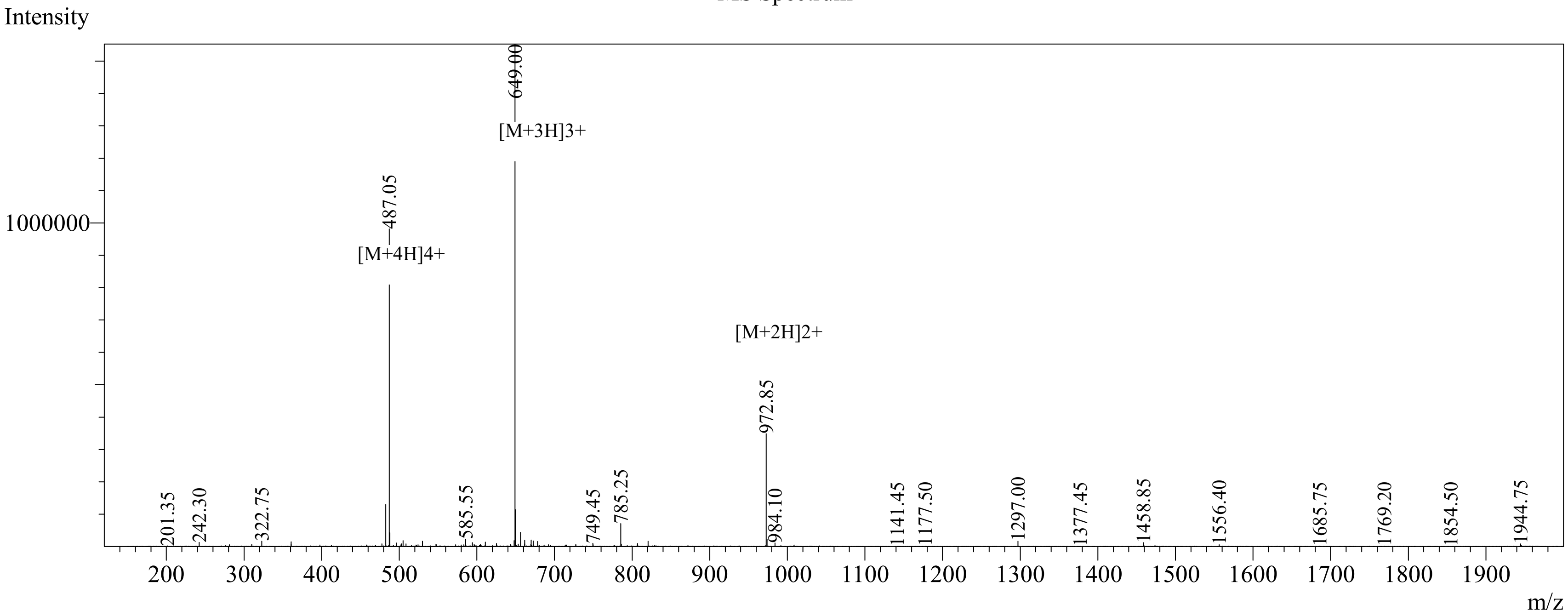

Sample Information
Dissolution method :0.1mg sample dissolved to 0.5mL by 100%H2O
Injection Volume :1ul

Name :P20571-14（Pep133-NGD)
Sequence :SAAERKHRHLPVADANGD
Modification :N/A
Lot No. :P20571-14（Pep133-NGD)
Theoretical :1944.082
observed :1944.00

Interface :ESI
Nebulizing Gas Flow :1.50L/min
CDL Temp :250°C
CDL Volt :0v
Block Temp :200

Prerod Bias :+4.5kv
Detector :-0.2kv
T.Flow :0.2ml/min
B.conc :50%H2O/50%MeOH

生工生物工程（上海）股份有限公司

地址：上海市松江区香闵路698号
Add: 698 Xiang Min Road Songjiang Shanghai China
电话/Tel: 400-821-0268
传真/Fax: 86-21-37772170
邮箱/Email: Sales@sangon.com
网址/Web: wwww.sangon.com

MS Spectrum

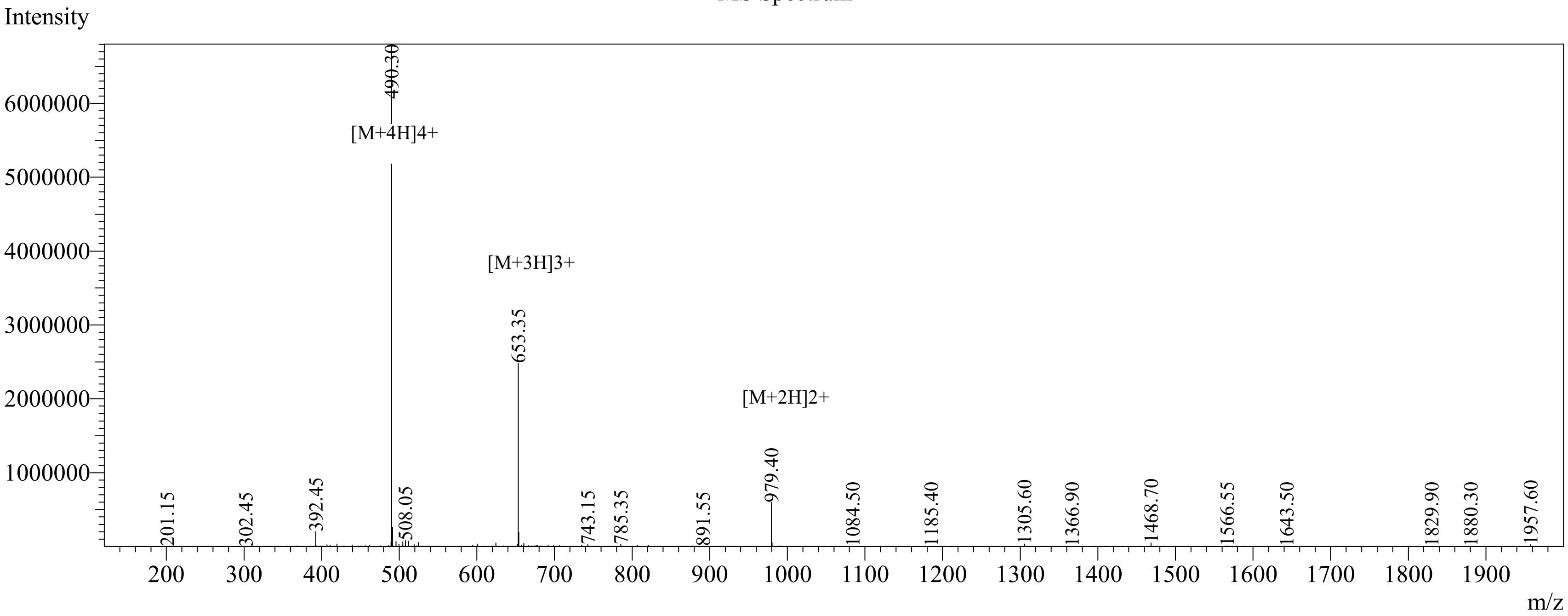

Sample Information
Dissolution method :0.1mg sample dissolved to 0.5mL by 100%H2O
Injection Volume :1ul
Name :P20571-15 (Pep133-NGK)
Sequence :SAAERKHRHLPVADANGK
Modification :N/A
Lot No. :P20571-15 (Pep133-NGK)
Theoretical :1957.163
observed :1957.20

Interface :ESI
Nebulizing Gas Flow :1.50L/min
CDL Temp :250℃
CDL Volt :0v
Block Temp :200

Prerod Bias :+4.5kv
Detector :-0.2kv
T.Flow :0.2ml/min
B.conc :50%H2O/50%MeOH

生工生物工程（上海）股份有限公司

地址：上海市松江区香闵路698号
电话/Tel: 400-821-0268
邮箱/Email: Sales@sangon.com

Add: 698 Xiang Min Road Songjiang Shanghai China
传真/Fax: 86-21-37772170
网址/Web: wwww.sangon.com

MS Spectrum

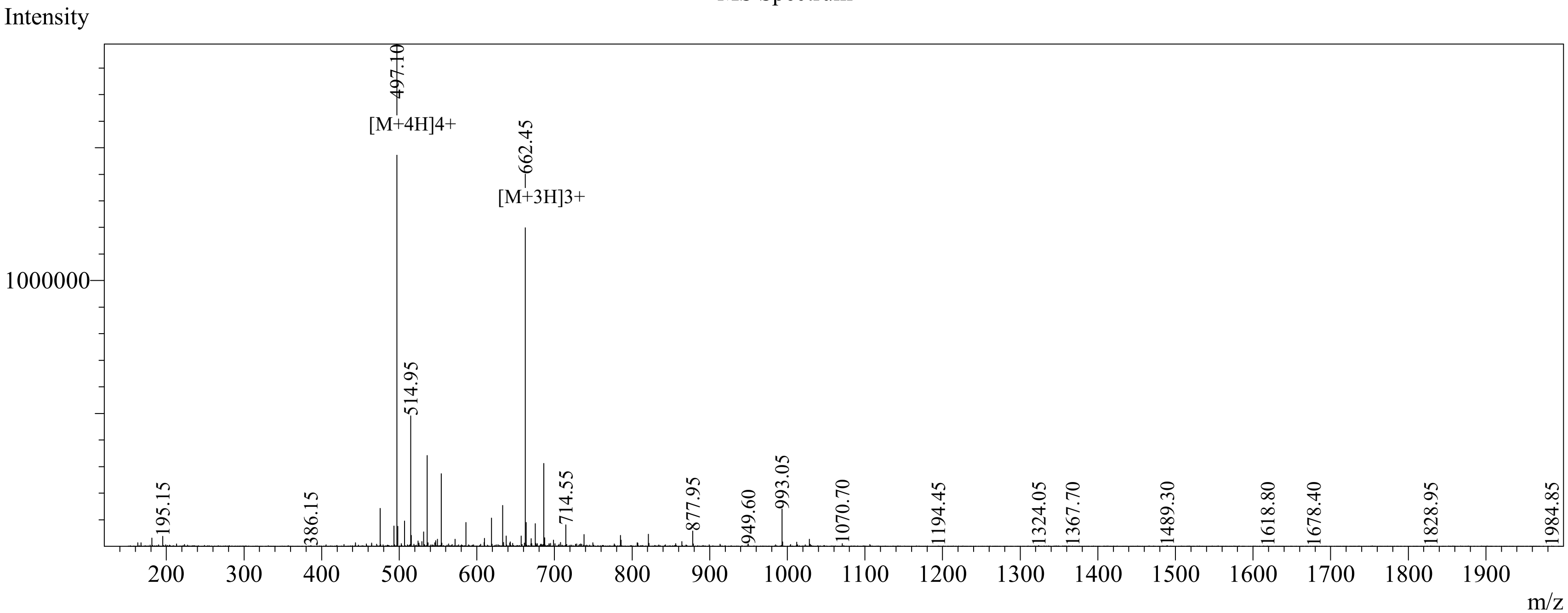

Sample Information
Dissolution method :0.1mg sample dissolved to 0.5mL by 100%H2O
Injection Volume :1ul

Name :P21421-3-Pep133-NVL
Sequence :SAAERKHRHLPVADANVL
Modification :N/A
Lot No. :P21421-3-Pep133-NVL
Theoretical :1984.234
bserved :1984.40

Interface :ESI
Nebulizing Gas Flow :1.50L/min
CDL Temp :250℃
CDL Volt :0v
Block Temp :200

Prerod Bias :+4.5kv
Detector :-0.2kv
T.Flow :0.2ml/min
B.conc :50%H2O/50%MeOH

生工生物工程（上海）股份有限公司

地址：上海市松江区香闵路698号
电话/Tel: 400-821-0268
邮箱/Email: Sales@sangon.com

Add: 698 Xiang Min Road Songjiang Shanghai China
传真/Fax: 86-21-37772170
网址/Web: wwww.sangon.com

MS Spectrum

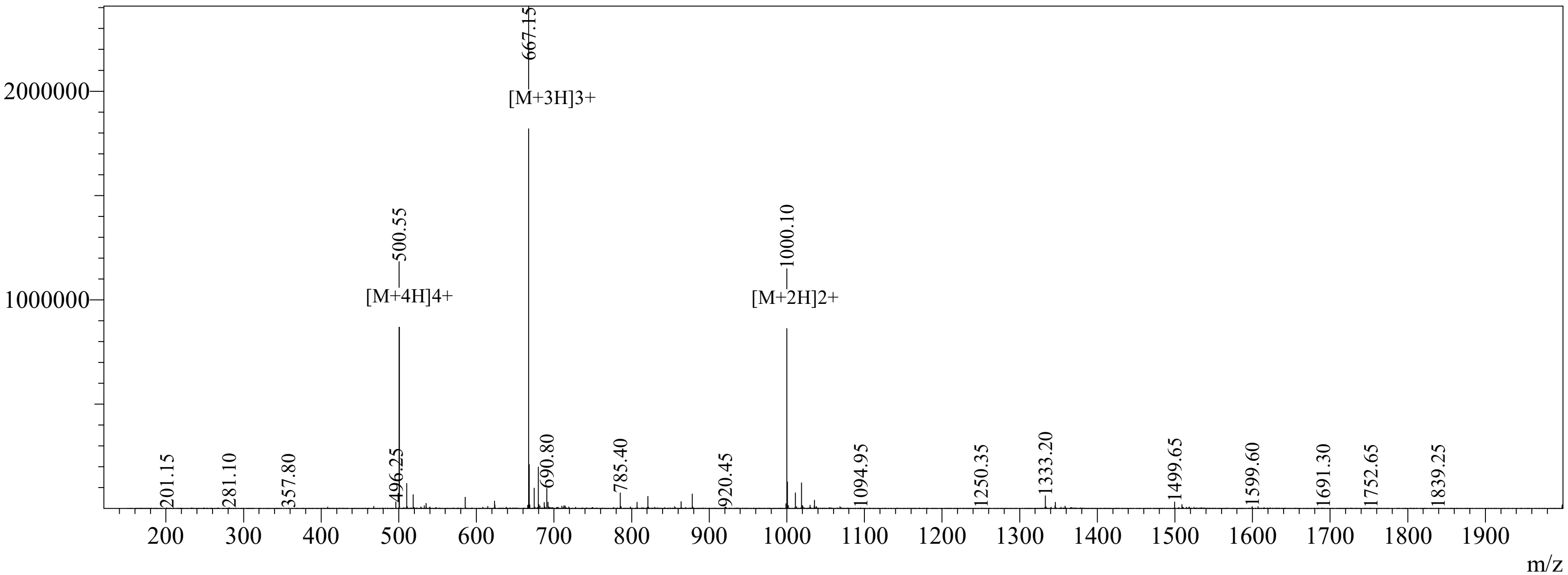

Sample Information
Dissolution method :0.1mg sample dissolved to 0.5mL by 100%H2O
Injection Volume :1ul

Name :P21421-4-Pep133-NLL
Sequence :SAAERKHRHLPVADANLL
Modification :N/A
Lot No. :P21421-4-Pep133-NLL
Theoretical :1998.261
observed :1998.45

Interface :ESI
Nebulizing Gas Flow :1.50L/min
CDL Temp :250°C
CDL Volt :0v
Block Temp :200

Prerod Bias :+4.5kv
Detector :-0.2kv
T.Flow :0.2ml/min
B.conc :50%H2O/50%MeOH

生工生物工程（上海）股份有限公司

地址：上海市松江区香闵路698号
电话/Tel: 400-821-0268
邮箱/Email: Sales@sangon.com

Add: 698 Xiang Min Road Songjiang Shanghai China
传真/Fax: 86-21-37772170
网址/Web: wwww.sangon.com

MS Spectrum

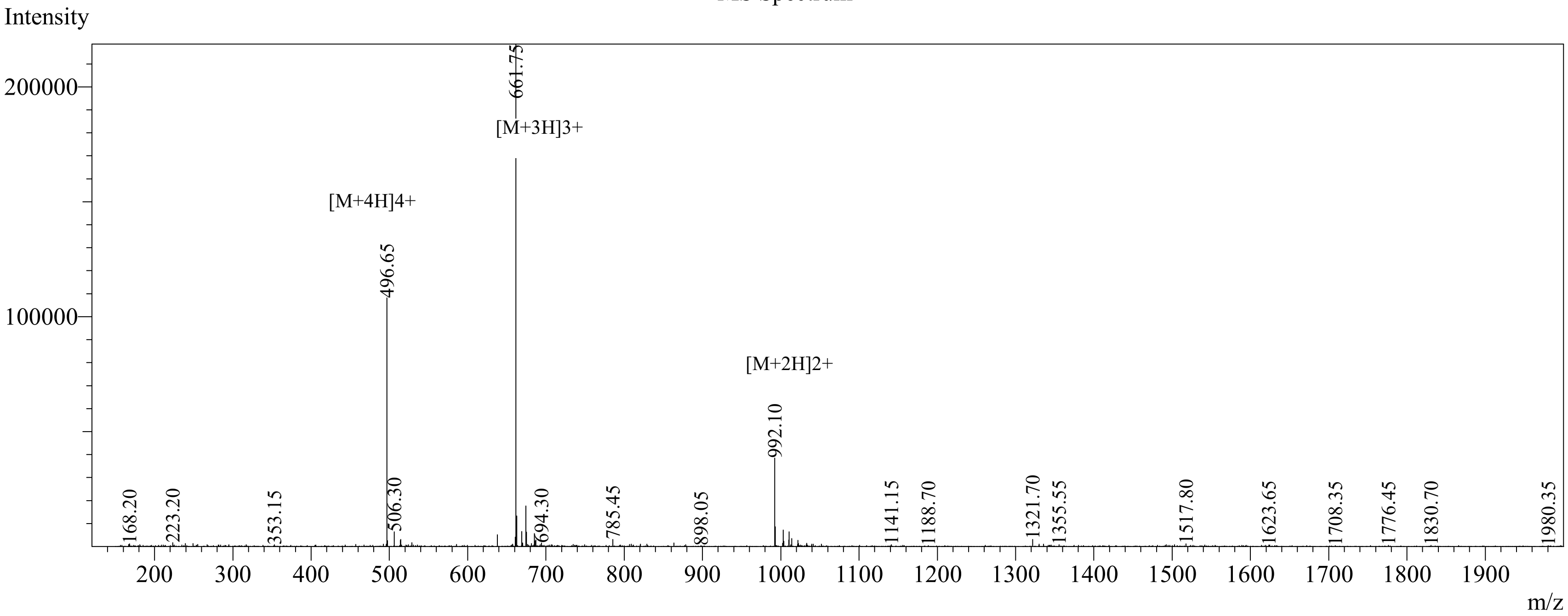

Sample Information
Dissolution method :0.1mg sample dissolved to 0.5mL by 100%H2O
Injection Volume :1ul
Name :P21421-6-Pep133-NPL
Sequence :SAAERKHRHLPVADANPL
Modification :N/A
Lot No. :P21421-6-Pep133-NPL
Theoretical :1982.218
observed :1982.25

Interface :ESI
Nebulizing Gas Flow :1.50L/min
CDL Temp :250℃
CDL Volt :0v
Block Temp :200

Prerod Bias :+4.5kv
Detector :-0.2kv
T.Flow :0.2ml/min
B.conc :50%H2O/50%MeOH

生工生物工程（上海）股份有限公司

地址：上海市松江区香闵路698号
Add: 698 Xiang Min Road Songjiang Shanghai China
电话/Tel: 400-821-0268
传真/Fax: 86-21-37772170
邮箱/Email: Sales@sangon.com
网址/Web: wwww.sangon.com

生工® Sangon Biotech

MS Spectrum

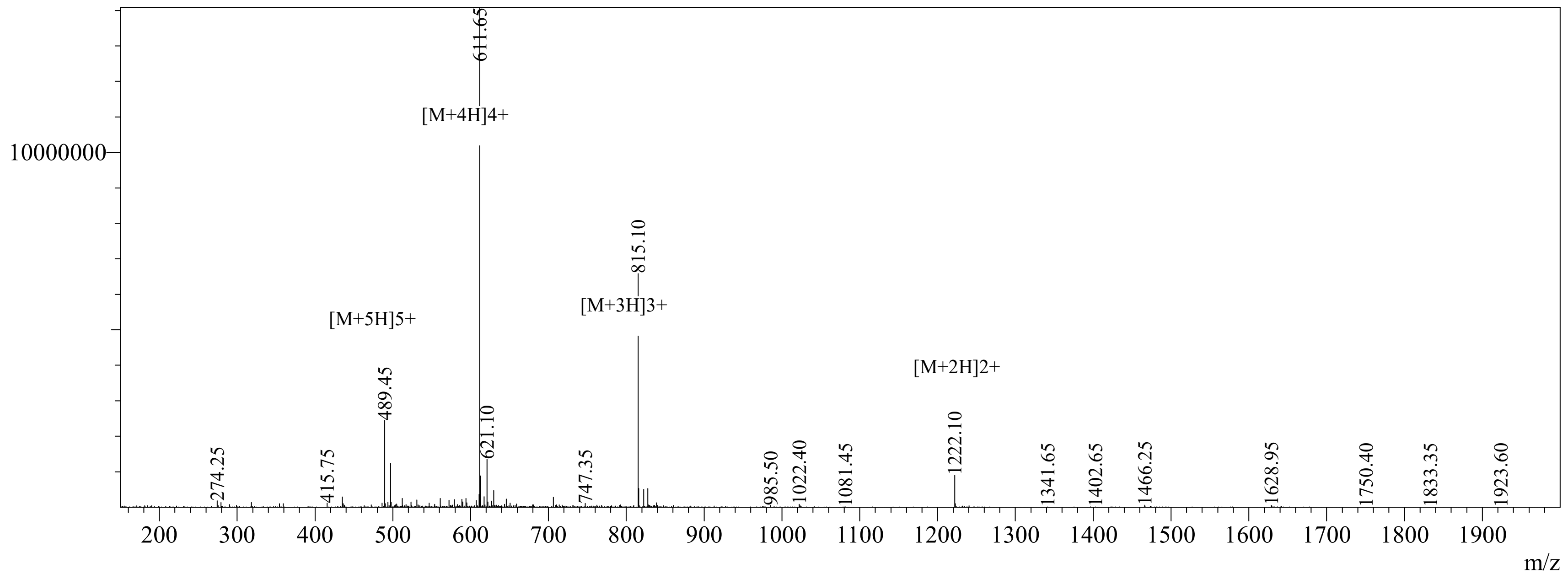

Sample Information
Dissolution method :0.1mg sample dissolved to 0.5mL by 50%ACN and 50%H2O
Injection Volume :1ul

Name	:P27704-2-Pep133-NAL (FAM labeled)
Sequence	:SAAERKHRHLPVADAK (Fam)NAL
Modification	:K (Fam)
Lot No.	:P27704-2-Pep133-NAL (FAM labeled)
Theoretical	:2442.65
observed	:2442.60

Interface	:ESI
Nebulizing Gas Flow	:1.50L/min
CDL Temp	:250℃
CDL Volt	:0v
Block Temp	:200

Prerod Bias	:+4.5kv
Detector	:-0.2kv
T.Flow	:0.2ml/min
B.conc	:50%H2O/50%MeOH

生工生物工程（上海）股份有限公司

地址：上海市松江区香闵路698号
Add: 698 Xiang Min Road Songjiang Shanghai China
电话/Tel: 400-821-0268
传真/Fax: 86-21-37772170
邮箱/Email: Sales@sangon.com
网址/Web: wwww.sangon.com

生工® Sangon Biotech

MS Spectrum

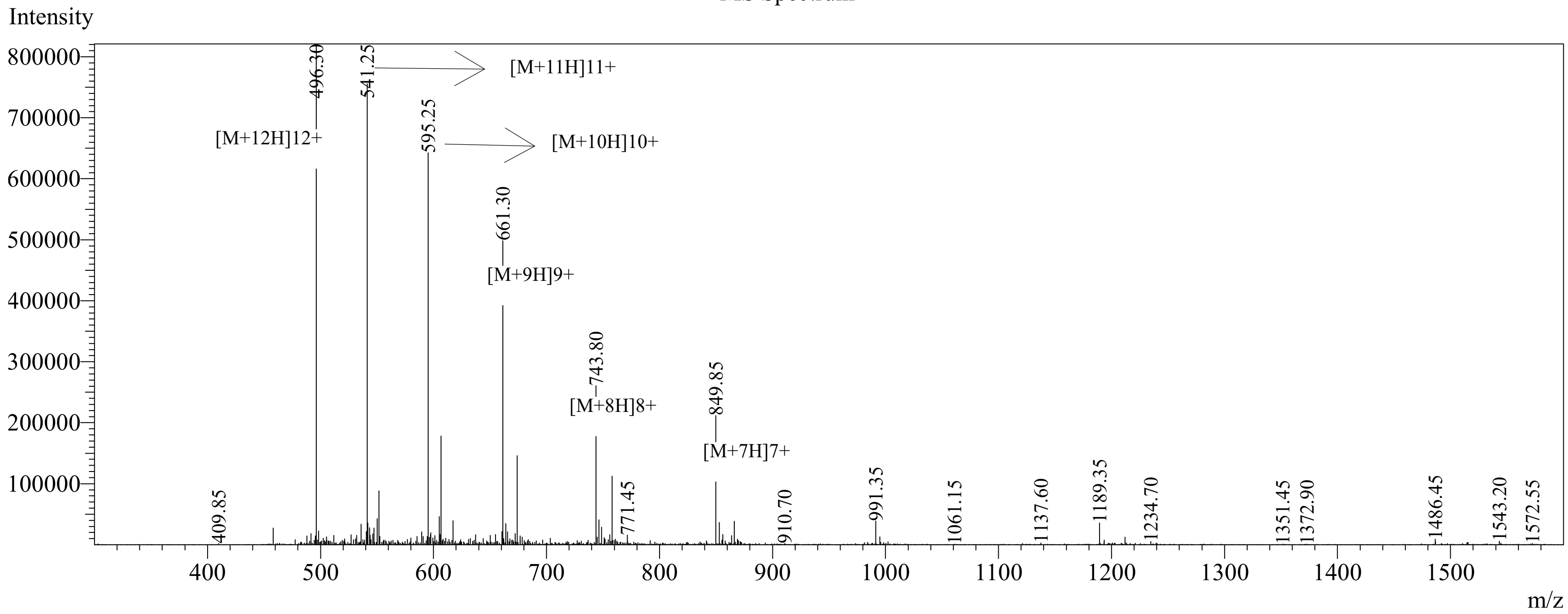

Sample Information
Dissolution method :0.1mg sample dissolved to 0.5mL by 100%H2O
Injection Volume :1ul

Interface :ESI
Nebulizing Gas Flow :1.50L/min
CDL Temp :250℃
CDL Volt :0v
Block Temp :200

Prerod Bias :+4.5kv
Detector :-0.2kv
T.Flow :0.2ml/min
B.conc :50%H2O/50%MeOH

Name :P20955-1 (C50-NAL)
Sequence :PQRKTKRNTNRRPQDVKFPGGGQIVGGVYLLPRRGPRLGVRATRKTSERSNAL
Modification :N/A
Lot No. :P20955-1 (C50-NAL)
Theoretical :5941.838
observed :5943.60

生工生物工程（上海）股份有限公司

地址：上海市松江区香闵路698号
Add: 698 Xiang Min Road Songjiang Shanghai China
电话/Tel: 400-821-0268
传真/Fax: 86-21-37772170
邮箱/Email: Sales@sangon.com
网址/Web: wwww.sangon.com

MS Spectrum

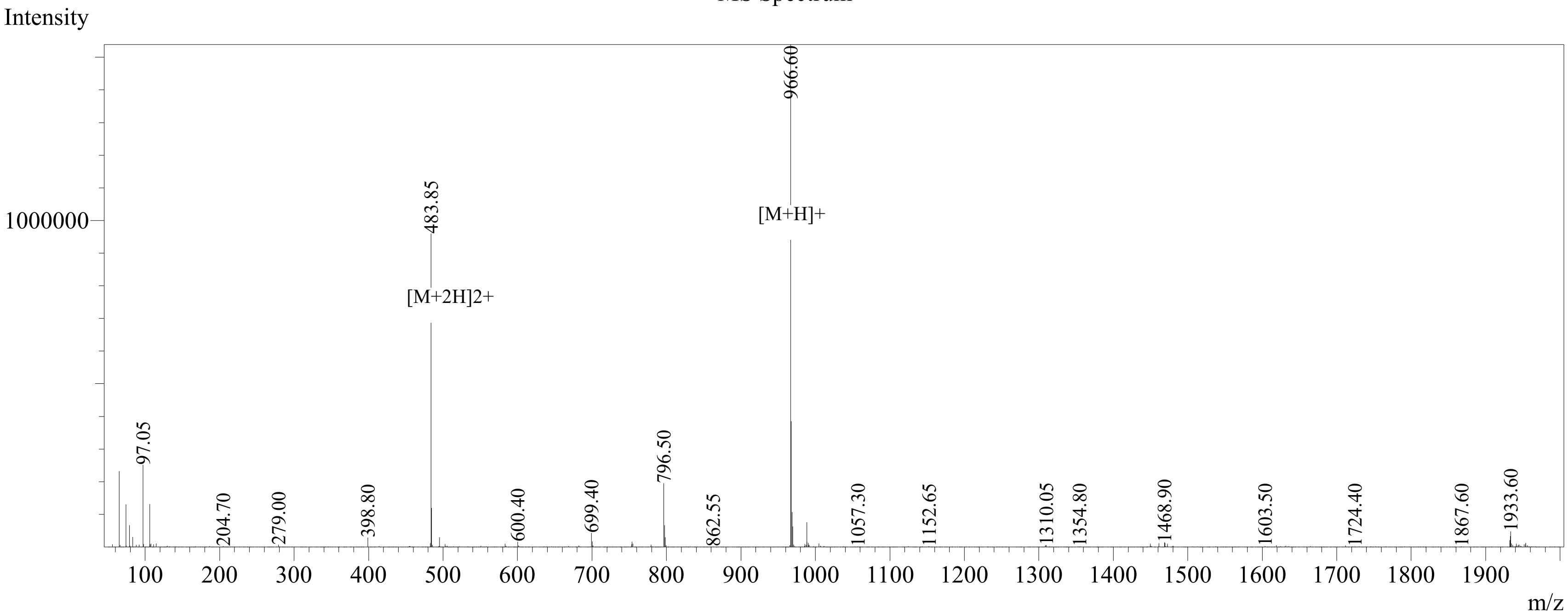

Sample Information
Dissolution method :0.1mg sample dissolved to 0.5mL by 100%H2O
Injection Volume :1ul
Name :P16698（GL-(biotin-labeled peptide)）
Sequence :GLPV(K-Biotin)AR
Modification :K-Biotin
Lot No. :P16698（GL-(biotin-labeled peptide)）
Theoretical :966.22
Observed :965.60

Interface :ESI
Nebulizing Gas Flow :1.50L/min
CDL Temp :250℃
CDL Volt :0v
Block Temp :200

Prerod Bias :+4.5kv
Detector :-0.2kv
T.Flow :0.2ml/min
B.conc :50%H2O/50%MeOH

生工生物工程（上海）股份有限公司

地址：上海市松江区香闵路698号
电话/Tel: 400-821-0268
邮箱/Email: Sales@sangon.com

Add: 698 Xiang Min Road Songjiang Shanghai China
传真/Fax: 86-21-37772170
网址/Web: wwww.sangon.com

MS Spectrum

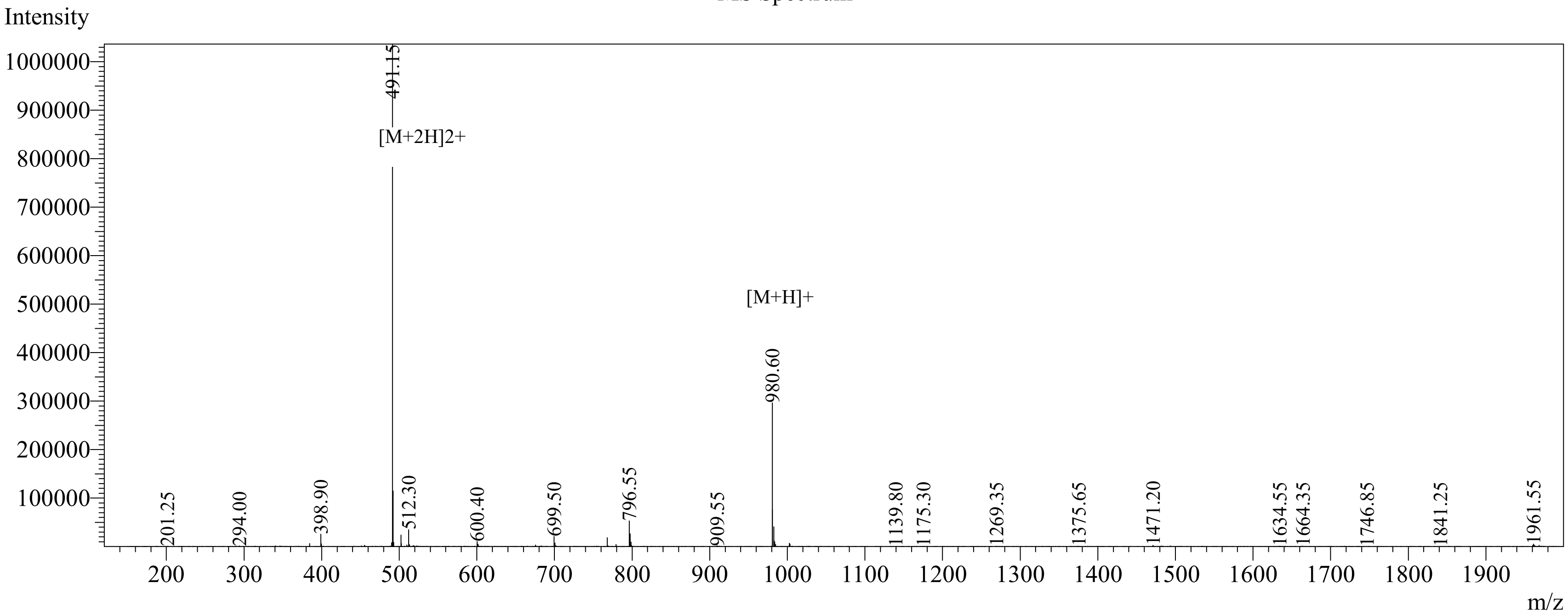

Sample Information
Dissolution method :0.1mg sample dissolved to 0.5mL by 100%H2O
Injection Volume :1ul
Name :P20955-2（AL-(biotin-labeled) peptide）
Sequence :ALPVKAR
Modification :K-Biotin
Lot No. :P20955-2（AL-(biotin-labeled) peptide）
Theoretical :980.236
Observed :980.30

Interface :ESI
Nebulizing Gas Flow :1.50L/min
CDL Temp :250℃
CDL Volt :0v
Block Temp :200

Prerod Bias :+4.5kv
Detector :-0.2kv
T.Flow :0.2ml/min
B.conc :50%H2O/50%MeOH

生工生物工程（上海）股份有限公司

地址：上海市松江区香闵路698号
Add: 698 Xiang Min Road Songjiang Shanghai China
电话/Tel: 400-821-0268
传真/Fax: 86-21-37772170
邮箱/Email: Sales@sangon.com
网址/Web: wwww.sangon.com

生工® Sangon Biotech

MS Spectrum

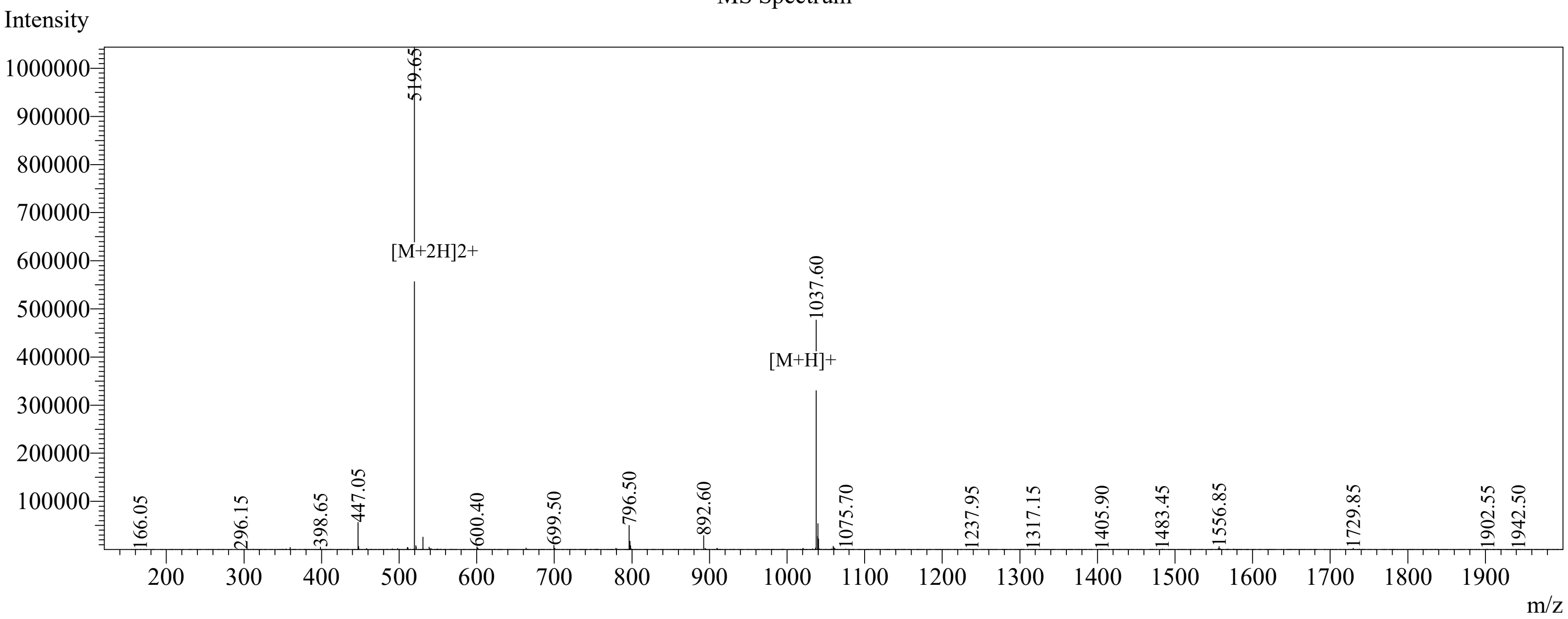

Sample Information
Dissolution method :0.1mg sample dissolved to 0.5mL by 100%H2O
Injection Volume :1ul
Name :P20955-3（QL-(biotin-labeled) peptide）
Sequence :QLPVKAR
Modification :K-Biotin
Lot No. :P20955-3（QL-(biotin-labeled) peptide）
Theoretical :1037.288
Observed :1037.30

Interface :ESI
Nebulizing Gas Flow :1.50L/min
CDL Temp :250°C
CDL Volt :0v
Block Temp :200

Prerod Bias :+4.5kv
Detector :-0.2kv
T.Flow :0.2ml/min
B.conc :50%H2O/50%MeOH

生工生物工程（上海）股份有限公司

地址：上海市松江区香闵路698号
Add: 698 Xiang Min Road Songjiang Shanghai China
电话/Tel: 400-821-0268
传真/Fax: 86-21-37772170
邮箱/Email: Sales@sangon.com
网址/Web: wwww.sangon.com

生工® Sangon Biotech

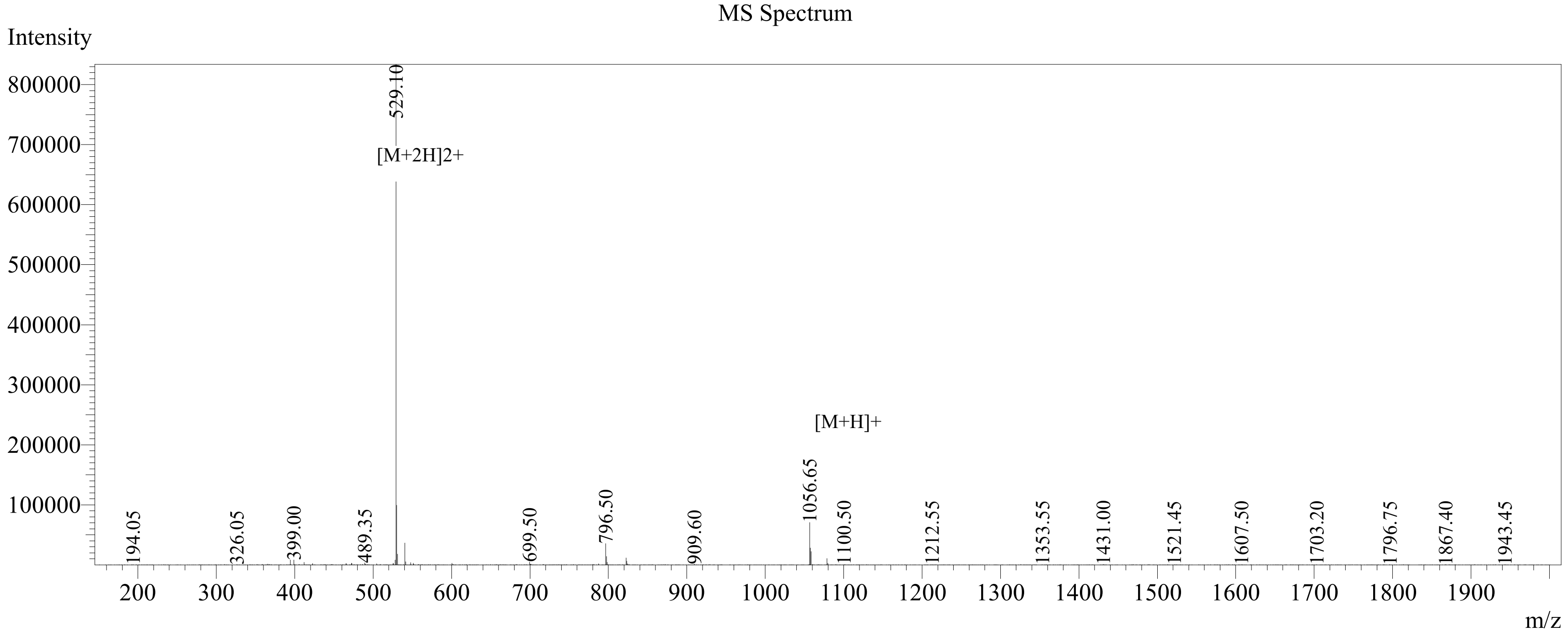

Sample Information
Dissolution method :0.1mg sample dissolved to 0.5mL by 100%H2O
Injection Volume :1ul
Name :P20955-4（FL-(biotin-labeled) peptide）
Sequence :FLPVKAR
Modification :K-Biotin
Lot No. :P20955-4（FL-(biotin-labeled) peptide）
Theoretical :1056.334
Observed :1056.20

Interface :ESI
Nebulizing Gas Flow :1.50L/min
CDL Temp :250℃
CDL Volt :0v
Block Temp :200

Prerod Bias :+4.5kv
Detector :-0.2kv
T.Flow :0.2ml/min
B.conc :50%H2O/50%MeOH

生工生物工程（上海）股份有限公司

地址：上海市松江区香闵路698号
电话/Tel: 400-821-0268
邮箱/Email: Sales@sangon.com

Add: 698 Xiang Min Road Songjiang Shanghai China
传真/Fax: 86-21-37772170
网址/Web: wwww.sangon.com

MS Spectrum

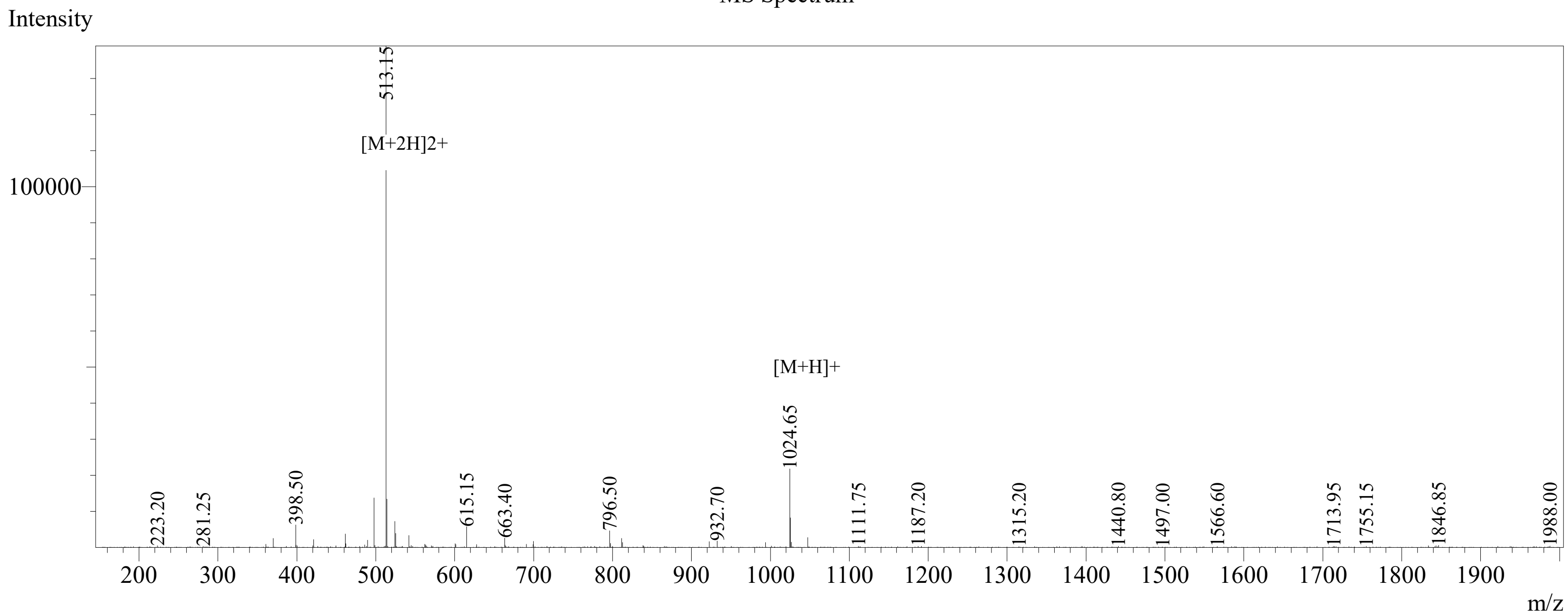

Sample Information
Dissolution method :0.1mg sample dissolved to 0.5mL by 100%H2O
Injection Volume :1ul
Name :P20955-5（DL-(biotin-labeled) peptide）
Sequence :DLPVKAR
Modification :K-Biotin
Lot No. :P20955-5（DL-(biotin-labeled) peptide）
Theoretical :1024.246
Observed :1024.30

Interface :ESI
Nebulizing Gas Flow :1.50L/min
CDL Temp :250℃
CDL Volt :0v
Block Temp :200

Prerod Bias :+4.5kv
Detector :-0.2kv
T.Flow :0.2ml/min
B.conc :50%H2O/50%MeOH

MS Spectrum

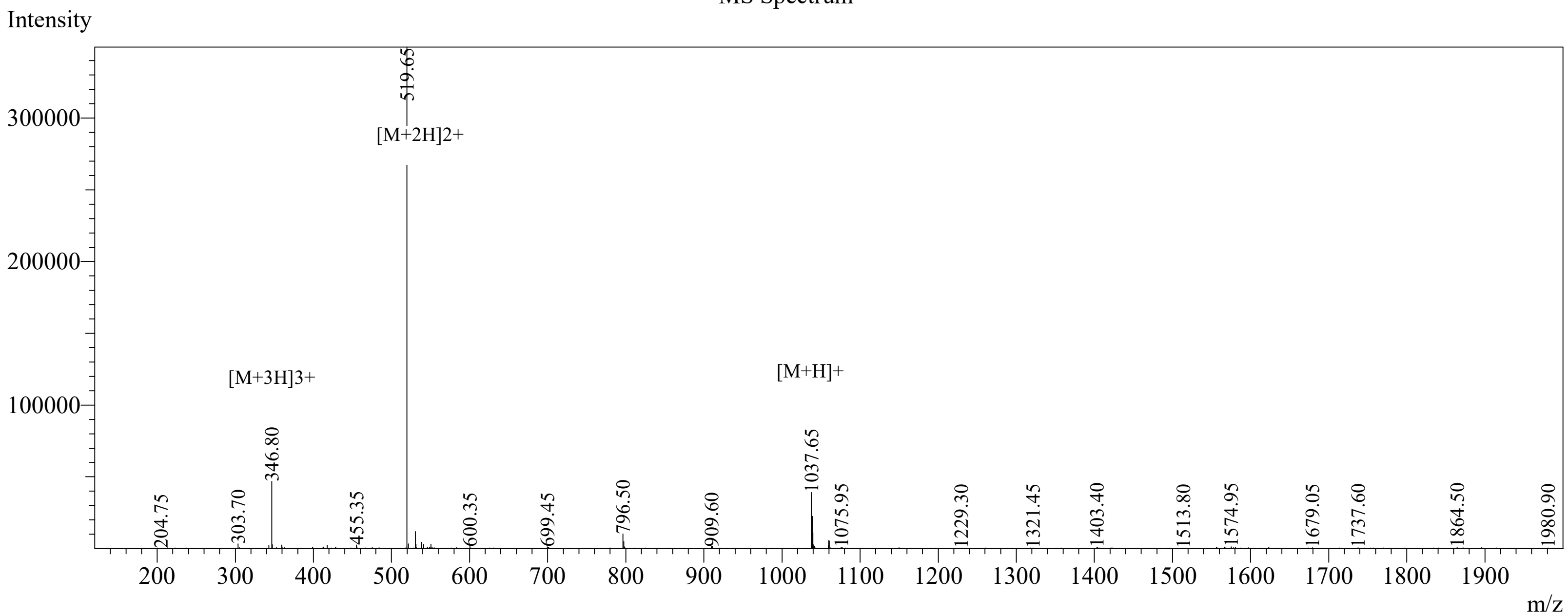

Sample Information
Dissolution method :0.1mg sample dissolved to 0.5mL by 100%H2O
Injection Volume :1ul
Name :P20955-6（KL-(biotin-labeled) peptide）
Sequence :KLPVKAR
Modification :K-Biotin
Lot No. :P20955-6（KL-(biotin-labeled) peptide）
Theoretical :1037.327
Observed :1037.30

Interface :ESI
Nebulizing Gas Flow :1.50L/min
CDL Temp :250°C
CDL Volt :0v
Block Temp :200

Prerod Bias :+4.5kv
Detector :-0.2kv
T.Flow :0.2ml/min
B.conc :50%H2O/50%MeOH

生工生物工程（上海）股份有限公司

地址：上海市松江区香闵路698号
电话/Tel: 400-821-0268
邮箱/Email: Sales@sangon.com

Add: 698 Xiang Min Road Songjiang Shanghai China
传真/Fax: 86-21-37772170
网址/Web: wwww.sangon.com

MS Spectrum

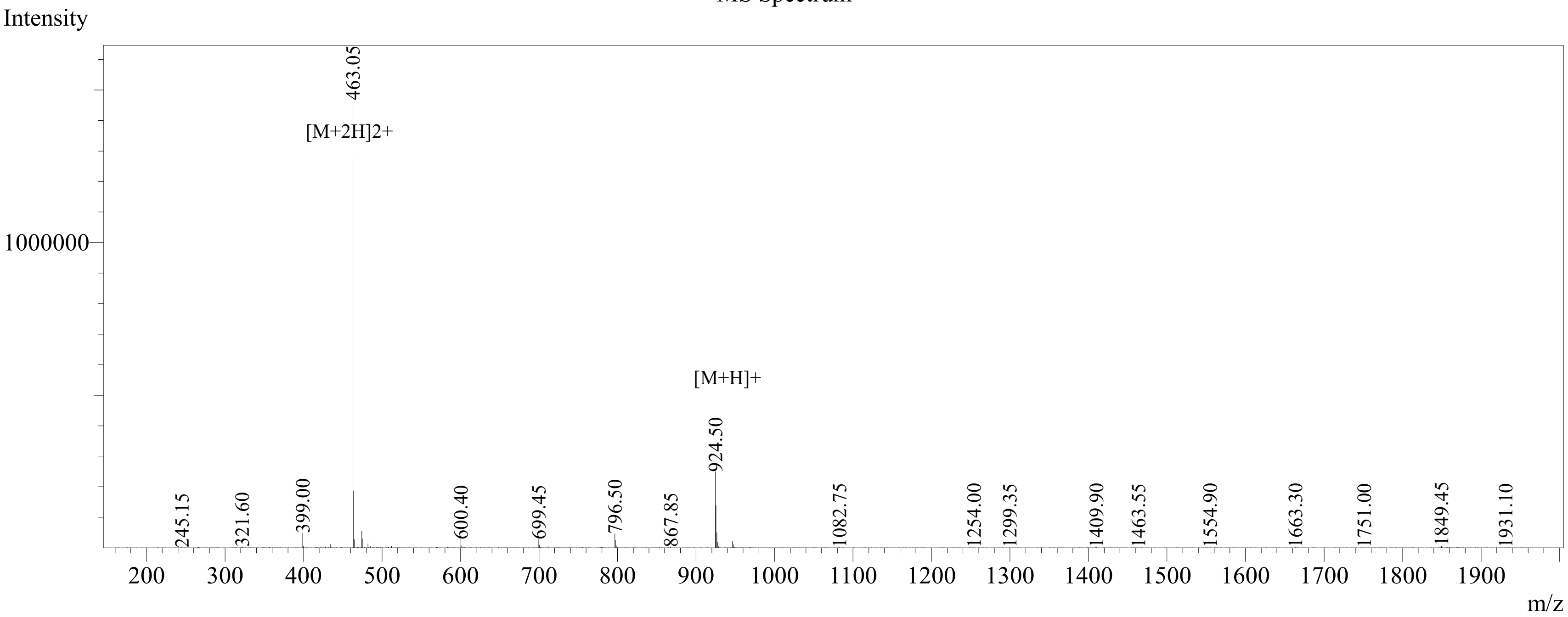

Sample Information
Dissolution method :0.1mg sample dissolved to 0.5mL by 100%H2O
Injection Volume :1ul
Name :P20955-7（GA-(biotin-labeled) peptide）
Sequence :GAPVKAR
Modification :K-Biotin
Lot No. :P20955-7（GA-(biotin-labeled) peptide）
Theoretical :924.128
Observed :924.10

Interface :ESI
Nebulizing Gas Flow :1.50L/min
CDL Temp :250℃
CDL Volt :0v
Block Temp :200

Prerod Bias :+4.5kv
Detector :-0.2kv
T.Flow :0.2ml/min
B.conc :50%H2O/50%MeOH

生工生物工程（上海）股份有限公司

地址：上海市松江区香闵路698号
Add: 698 Xiang Min Road Songjiang Shanghai China
电话/Tel: 400-821-0268
传真/Fax: 86-21-37772170
邮箱/Email: Sales@sangon.com
网址/Web: wwww.sangon.com

MS Spectrum

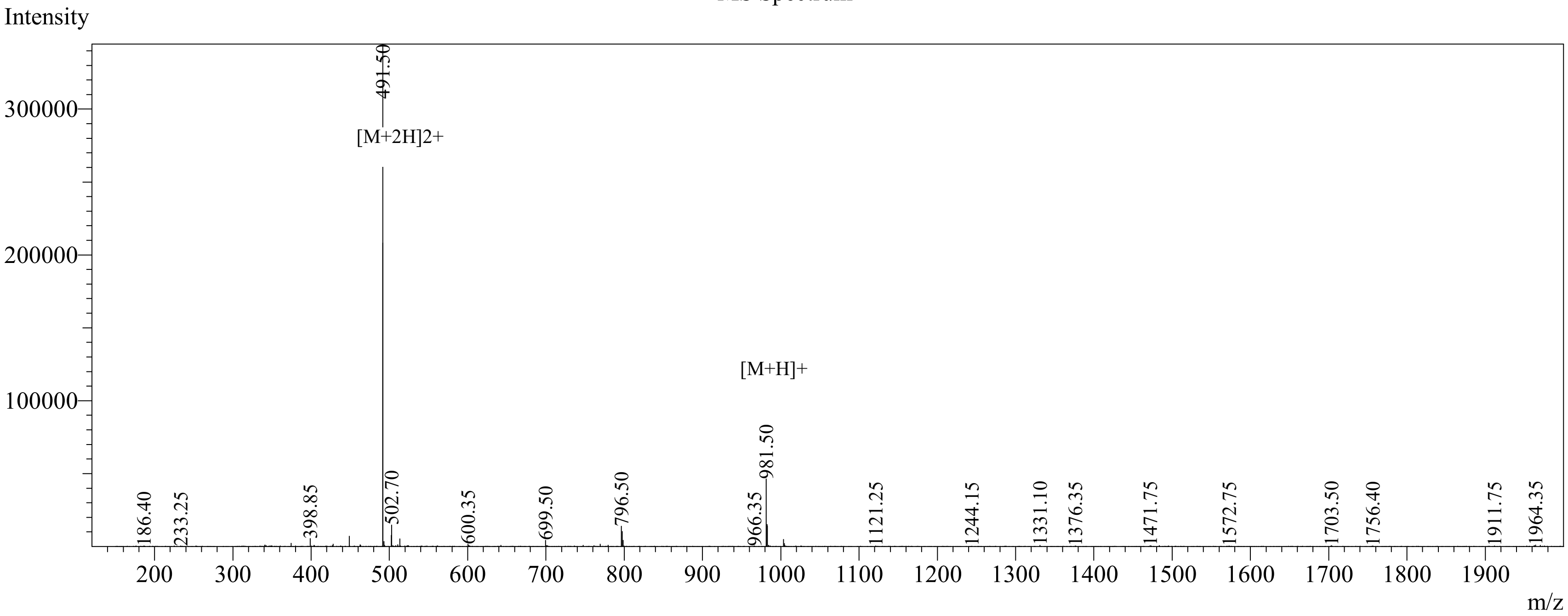

Sample Information
Dissolution method :0.1mg sample dissolved to 0.5mL by 100%H2O
Injection Volume :1ul
Name :P20955-8（GQ-(biotin-labeled) peptide）
Sequence :GQPVKAR
Modification :K-Biotin
Lot No. :P20955-8（GQ-(biotin-labeled) peptide）
Theoretical :981.18
Observed :981.00

Interface :ESI
Nebulizing Gas Flow :1.50L/min
CDL Temp :250℃
CDL Volt :0v
Block Temp :200

Prerod Bias :+4.5kv
Detector :-0.2kv
T.Flow :0.2ml/min
B.conc :50%H2O/50%MeOH

生工生物工程（上海）股份有限公司

地址：上海市松江区香闵路698号
Add: 698 Xiang Min Road Songjiang Shanghai China
电话/Tel: 400-821-0268
传真/Fax: 86-21-37772170
邮箱/Email: Sales@sangon.com
网址/Web: wwww.sangon.com

MS Spectrum

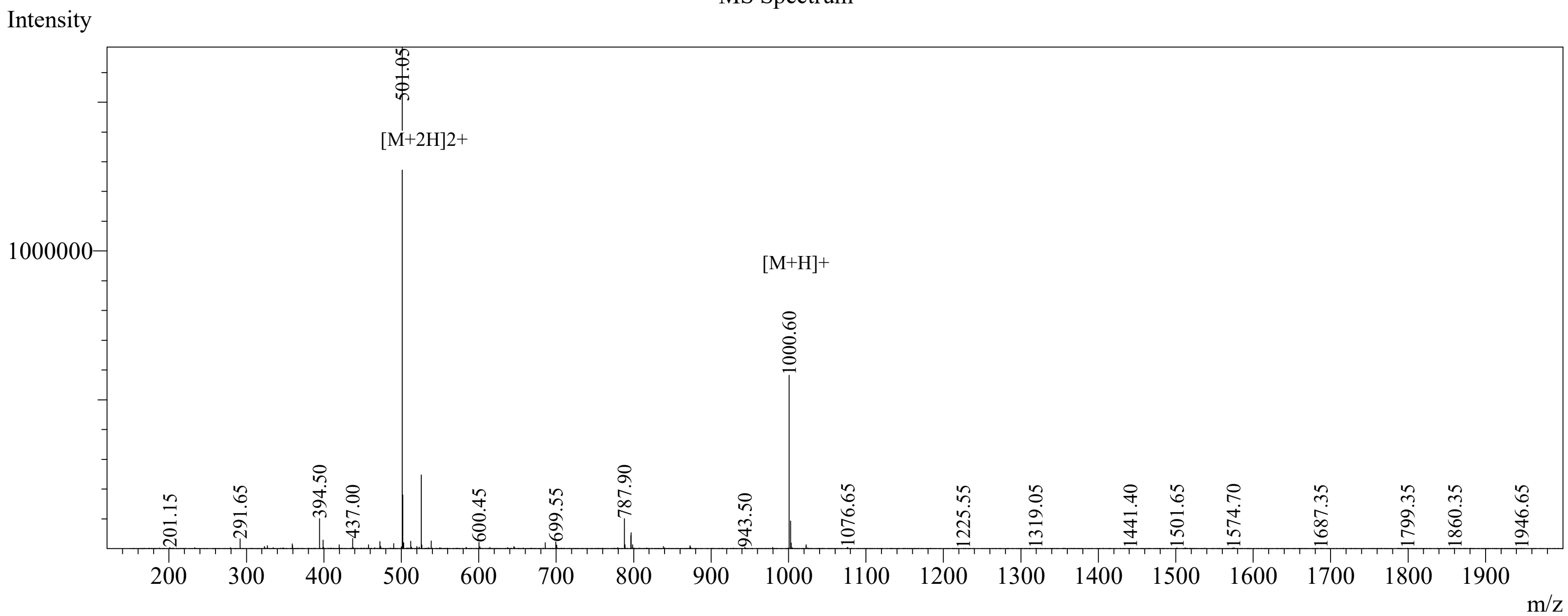

Sample Information
Dissolution method :0.1mg sample dissolved to 0.5mL by 100%H2O
Injection Volume :1ul

Name :P20955-9（GF-(biotin-labeled) peptide)
Sequence :GFPVKAR
Modification :K-Biotin
Lot No. :P20955-9（GF-(biotin-labeled) peptide)
Theoretical :1000.226
Observed :1000.10

Interface :ESI
Nebulizing Gas Flow :1.50L/min
CDL Temp :250°C
CDL Volt :0v
Block Temp :200

Prerod Bias :+4.5kv
Detector :-0.2kv
T.Flow :0.2ml/min
B.conc :50%H2O/50%MeOH

生工生物工程（上海）股份有限公司

地址：上海市松江区香闵路698号
电话/Tel: 400-821-0268
邮箱/Email: Sales@sangon.com

Add: 698 Xiang Min Road Songjiang Shanghai China
传真/Fax: 86-21-37772170
网址/Web: wwww.sangon.com

MS Spectrum

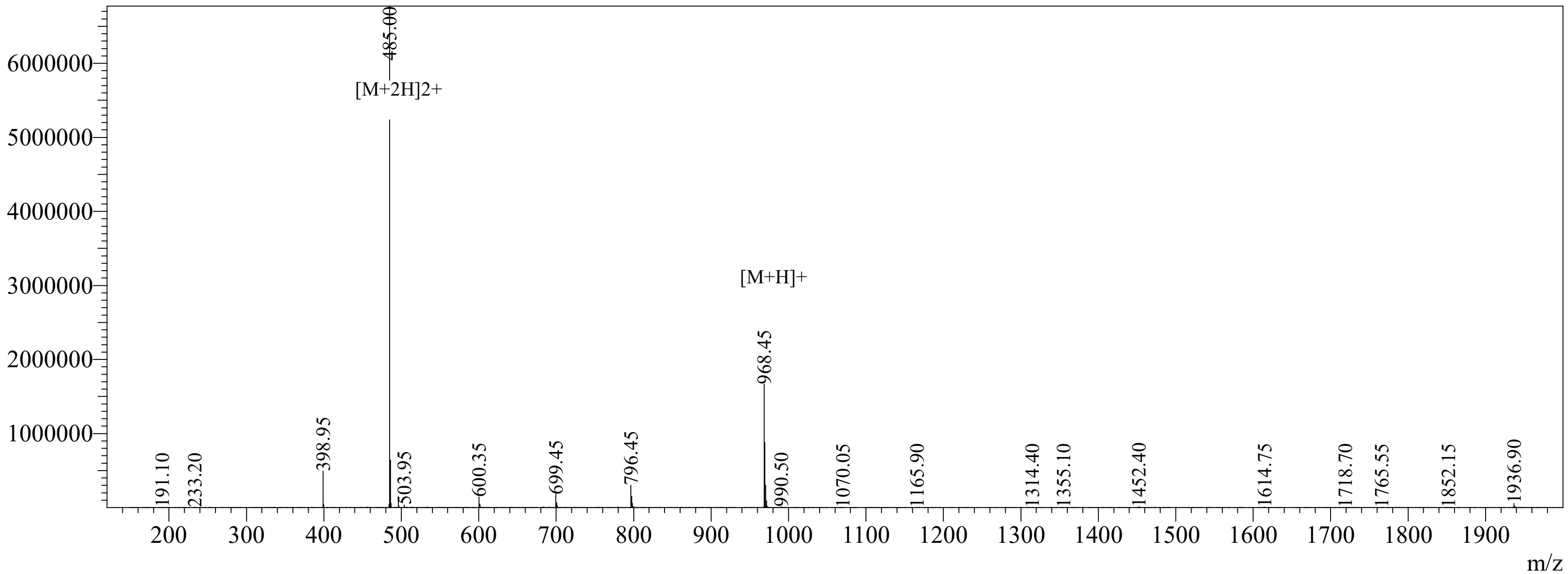

Sample Information
Dissolution method :0.1mg sample dissolved to 0.5mL by 100%H2O
Injection Volume :1ul
Name :P20955-10（GD-(biotin-labeled) peptide）
Sequence :GDPVKAR
Modification :K-Biotin
Lot No. :P20955-10（GD-(biotin-labeled) peptide）
Theoretical :968.138
Observed :968.00

Interface :ESI
Nebulizing Gas Flow :1.50L/min
CDL Temp :250°C
CDL Volt :0v
Block Temp :200

Prerod Bias :+4.5kv
Detector :-0.2kv
T.Flow :0.2ml/min
B.conc :50%H2O/50%MeOH

生工生物工程（上海）股份有限公司

地址：上海市松江区香闵路698号
电话/Tel: 400-821-0268
邮箱/Email: Sales@sangon.com

Add: 698 Xiang Min Road Songjiang Shanghai China
传真/Fax: 86-21-37772170
网址/Web: wwww.sangon.com

MS Spectrum

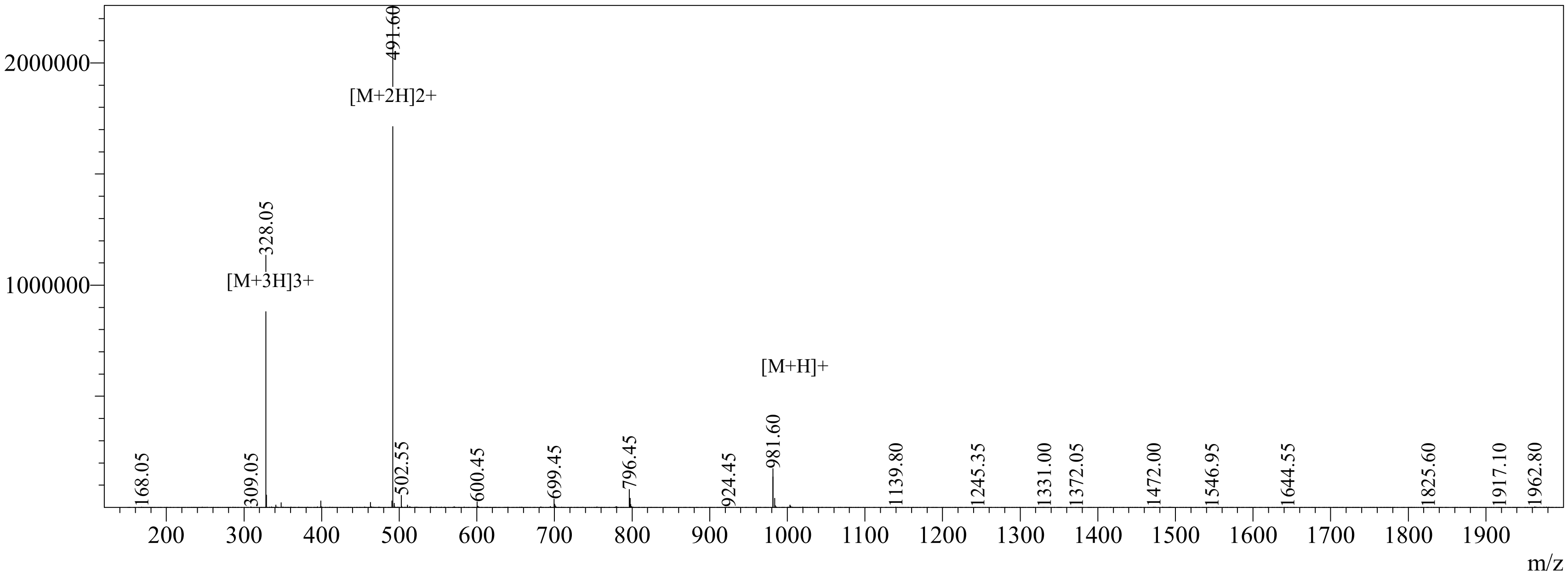

Sample Information
Dissolution method :0.1mg sample dissolved to 0.5mL by 100%H2O
Injection Volume :1ul

Interface	:ESI
Nebulizing Gas Flow	:1.50L/min
CDL Temp	:250℃
CDL Volt	:0v
Block Temp	:200

Prerod Bias	:+4.5kv
Detector	:-0.2kv
T.Flow	:0.2ml/min
B.conc	:50%H2O/50%MeOH

Name	:P20955-11（GK-(biotin-labeled) peptide）
Sequence	:GKPVKAR
Modification	:K-Biotin
Lot No.	:P20955-11（GK-(biotin-labeled) peptide）
Theoretical	:981.219
Observed	:981.20

生工生物工程（上海）股份有限公司

地址：上海市松江区香闵路698号
电话/Tel: 400-821-0268
邮箱/Email: Sales@sangon.com
Add: 698 Xiang Min Road Songjiang Shanghai China
传真/Fax: 86-21-37772170
网址/Web: wwww.sangon.com

生工® Sangon Biotech

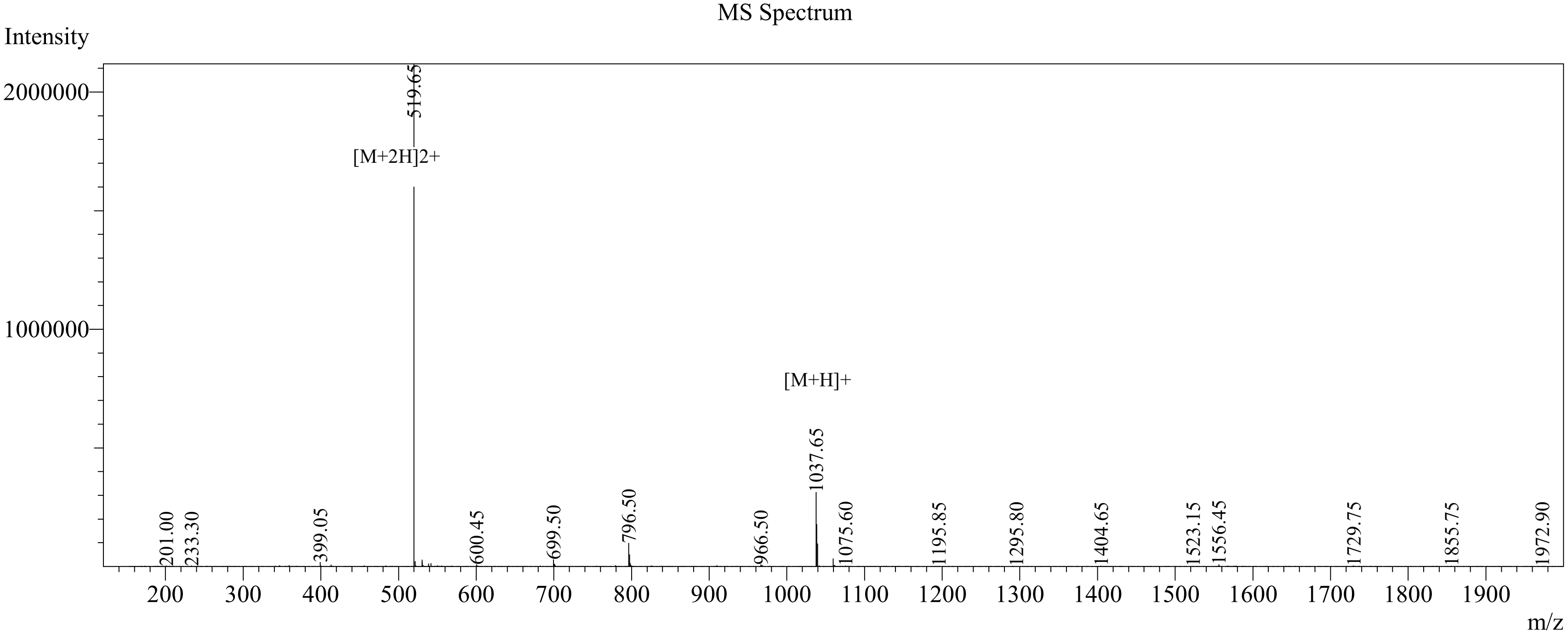

Sample Information
Dissolution method :0.1mg sample dissolved to 0.5mL by 100%H2O
Injection Volume :1ul
Name :P20955-12 AGL-(biotin-labeled peptide)
Sequence :AGLPVKAR
Modification :K-Biotin
Lot No. :P20955-12 AGL-(biotin-labeled peptide)
Theoretical :1037.288
observed :1037.30

Interface :ESI
Nebulizing Gas Flow :1.50L/min
CDL Temp :250℃
CDL Volt :0v
Block Temp :200

Prerod Bias :+4.5kv
Detector :-0.2kv
T.Flow :0.2ml/min
B.conc :50%H2O/50%MeOH

生工生物工程（上海）股份有限公司

地址：上海市松江区香闵路698号
电话/Tel: 400-821-0268
邮箱/Email: Sales@sangon.com

Add: 698 Xiang Min Road Songjiang Shanghai China
传真/Fax: 86-21-37772170
网址/Web: wwww.sangon.com

生工® Sangon Biotech

MS Spectrum

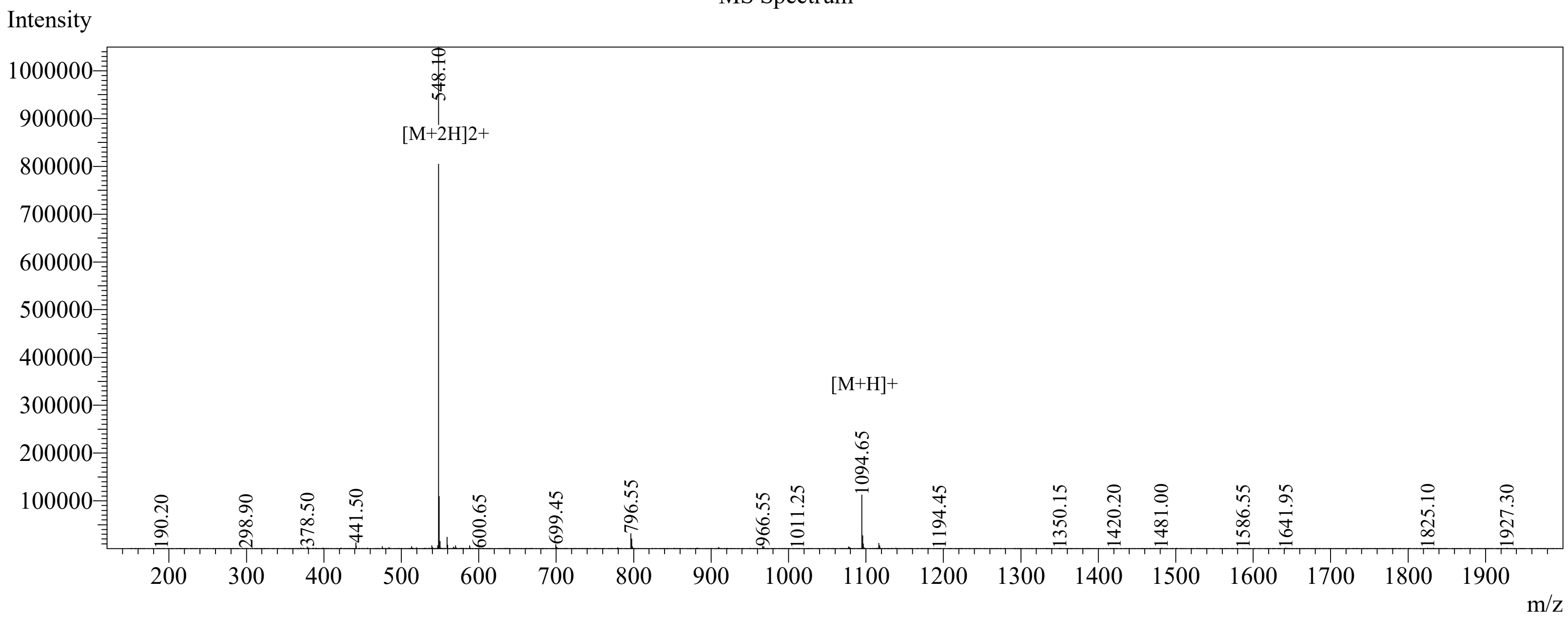

Sample Information
Dissolution method :0.1mg sample dissolved to 0.5mL by 100%H2O
Injection Volume :1ul

Name :P20955-13 QGL-(biotin-labeled peptide)
Sequence :QGLPVKAR
Modification :K-Biotin
Lot No. :P20955-13 QGL-(biotin-labeled peptide)
Theoretical :1094.34
bserved :1094.20

Interface :ESI
Nebulizing Gas Flow :1.50L/min
CDL Temp :250°C
CDL Volt :0v
Block Temp :200

Prerod Bias :+4.5kv
Detector :-0.2kv
T.Flow :0.2ml/min
B.conc :50%H2O/50%MeOH

生工生物工程（上海）股份有限公司

地址：上海市松江区香闵路698号
电话/Tel: 400-821-0268
邮箱/Email: Sales@sangon.com

Add: 698 Xiang Min Road Songjiang Shanghai China
传真/Fax: 86-21-37772170
网址/Web: wwww.sangon.com

MS Spectrum

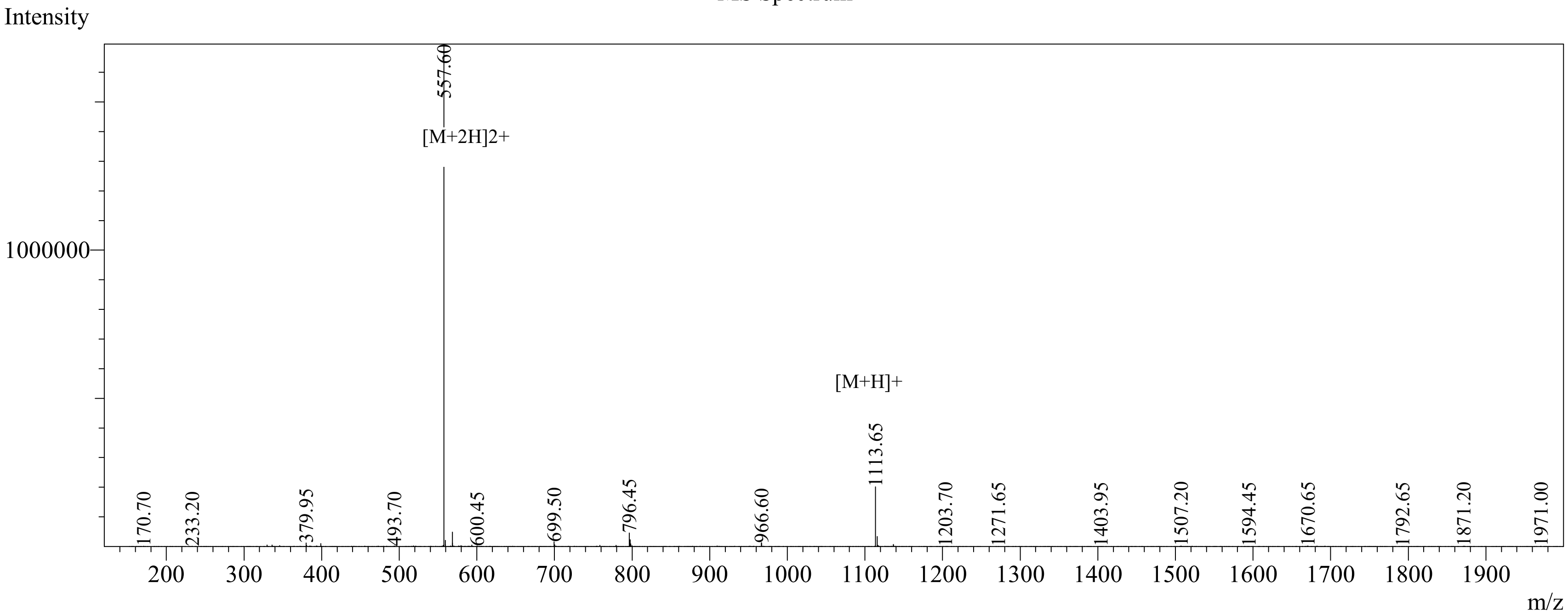

Sample Information
Dissolution method :0.1mg sample dissolved to 0.5mL by 100%H2O
Injection Volume :1ul
Name :P20955-14 FGL-(biotin-labeled peptide)
Sequence :FGLPVKAR
Modification :K-Biotin
Lot No. :P20955-14 FGL-(biotin-labeled peptide)
Theoretical :1113.386
observed :1113.20

Interface :ESI
Nebulizing Gas Flow :1.50L/min
CDL Temp :250℃
CDL Volt :0v
Block Temp :200

Prerod Bias :+4.5kv
Detector :-0.2kv
T.Flow :0.2ml/min
B.conc :50%H2O/50%MeOH

生工生物工程（上海）股份有限公司

地址：上海市松江区香闵路698号
Add: 698 Xiang Min Road Songjiang Shanghai China
电话/Tel: 400-821-0268
传真/Fax: 86-21-37772170
邮箱/Email: Sales@sangon.com
网址/Web: wwww.sangon.com

MS Spectrum

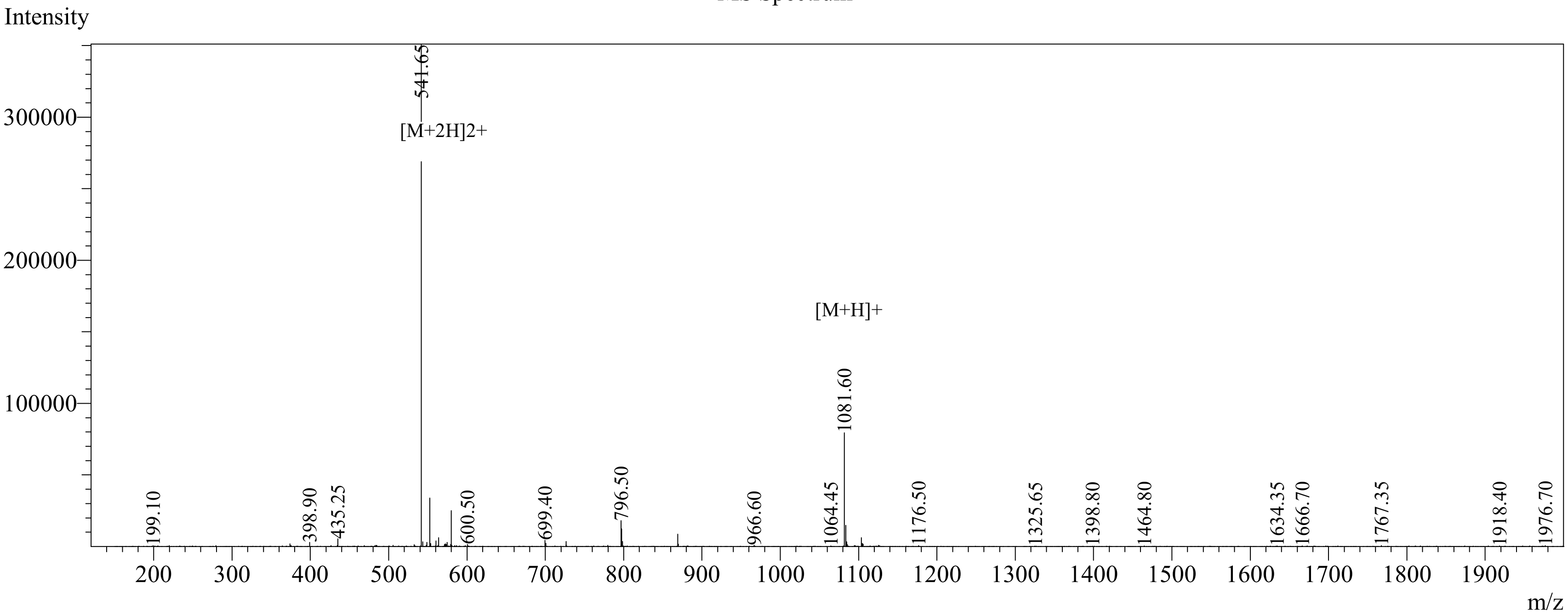

Sample Information
Dissolution method :0.1mg sample dissolved to 0.5mL by 100%H2O
Injection Volume :1ul
Name :P20955-15 DGL-(biotin-labeled peptide)
Sequence :DGLPVKAR
Modification :K-Biotin
Lot No. :P20955-15 DGL-(biotin-labeled peptide)
Theoretical :1081.298
Observed :1081.30

Interface :ESI
Nebulizing Gas Flow :1.50L/min
CDL Temp :250℃
CDL Volt :0v
Block Temp :200

Prerod Bias :+4.5kv
Detector :-0.2kv
T.Flow :0.2ml/min
B.conc :50%H2O/50%MeOH

生工生物工程（上海）股份有限公司

地址：上海市松江区香闵路698号
电话/Tel: 400-821-0268
邮箱/Email: Sales@sangon.com

Add: 698 Xiang Min Road Songjiang Shanghai China
传真/Fax: 86-21-37772170
网址/Web: wwww.sangon.com

MS Spectrum

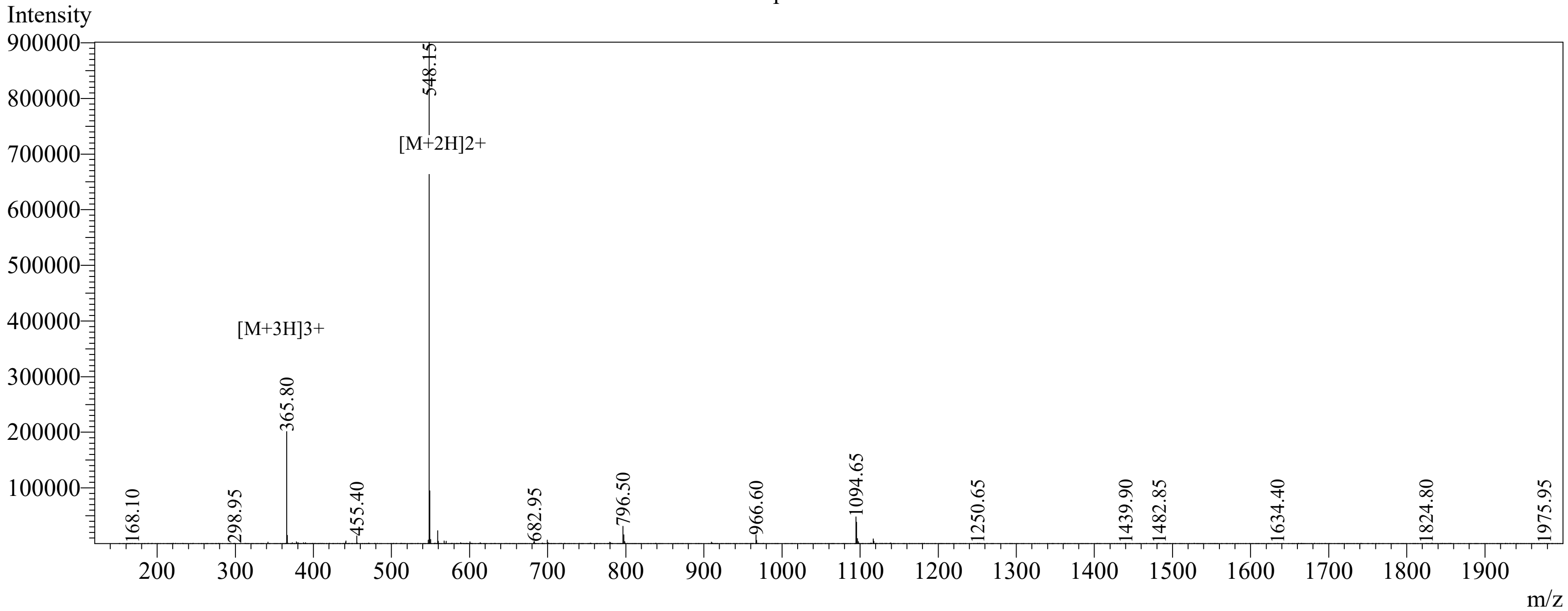

Sample Information
Dissolution method :0.1mg sample dissolved to 0.5mL by 100%H2O
Injection Volume :1ul
Name :P20955-16 KGL-(biotin-labeled peptide)
Sequence :KGLPVKAR
Modification :K-Biotin
Lot No. :P20955-16 KGL-(biotin-labeled peptide)
Theoretical :1094.379
Observed :1094.3[illegible]

Interface :ESI
Nebulizing Gas Flow :1.50L/min
CDL Temp :250℃
CDL Volt :0v
Block Temp :200

Prerod Bias :+4.5kv
Detector :-0.2kv
T.Flow :0.2ml/min
B.conc :50%H2O/50%MeOH

MS Spectrum

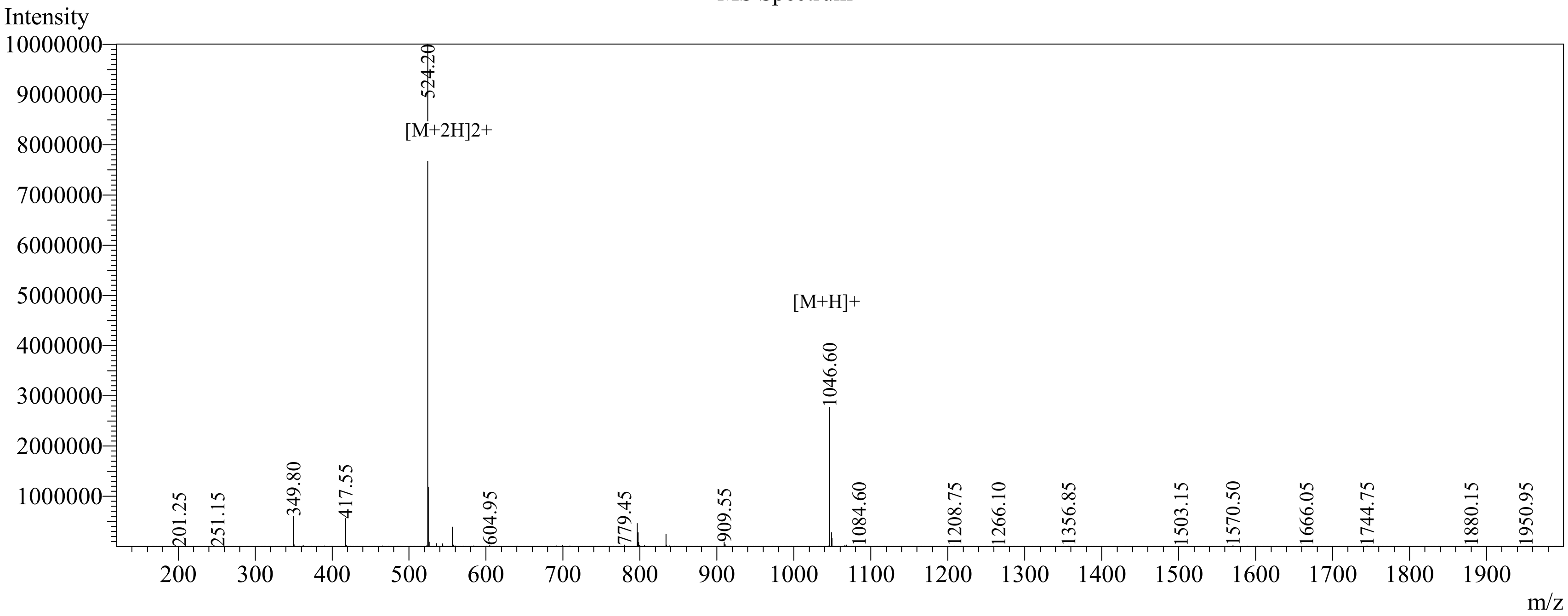

Sample Information
Dissolution method :0.1mg sample dissolved to 0.5mL by 100%H2O
Injection Volume :1ul
Name :P21421-2-HL-(biotin labeled)peptide
Sequence :HLPVK(Biotin)AR
Modification :K(Biotin)
Lot No. :P21421-2-HL-(biotin labeled)peptide
Theoretical :1046.298
Observed :1046.40

Interface :ESI
Nebulizing Gas Flow :1.50L/min
CDL Temp :250℃
CDL Volt :0v
Block Temp :200

Prerod Bias :+4.5kv
Detector :-0.2kv
T.Flow :0.2ml/min
B.conc :50%H2O/50%MeOH

生工生物工程（上海）股份有限公司

地址：上海市松江区香闵路698号
Add: 698 Xiang Min Road Songjiang Shanghai China
电话/Tel: 400-821-0268
传真/Fax: 86-21-37772170
邮箱/Email: Sales@sangon.com
网址/Web: wwww.sangon.com

生工® Sangon Biotech

MS Spectrum

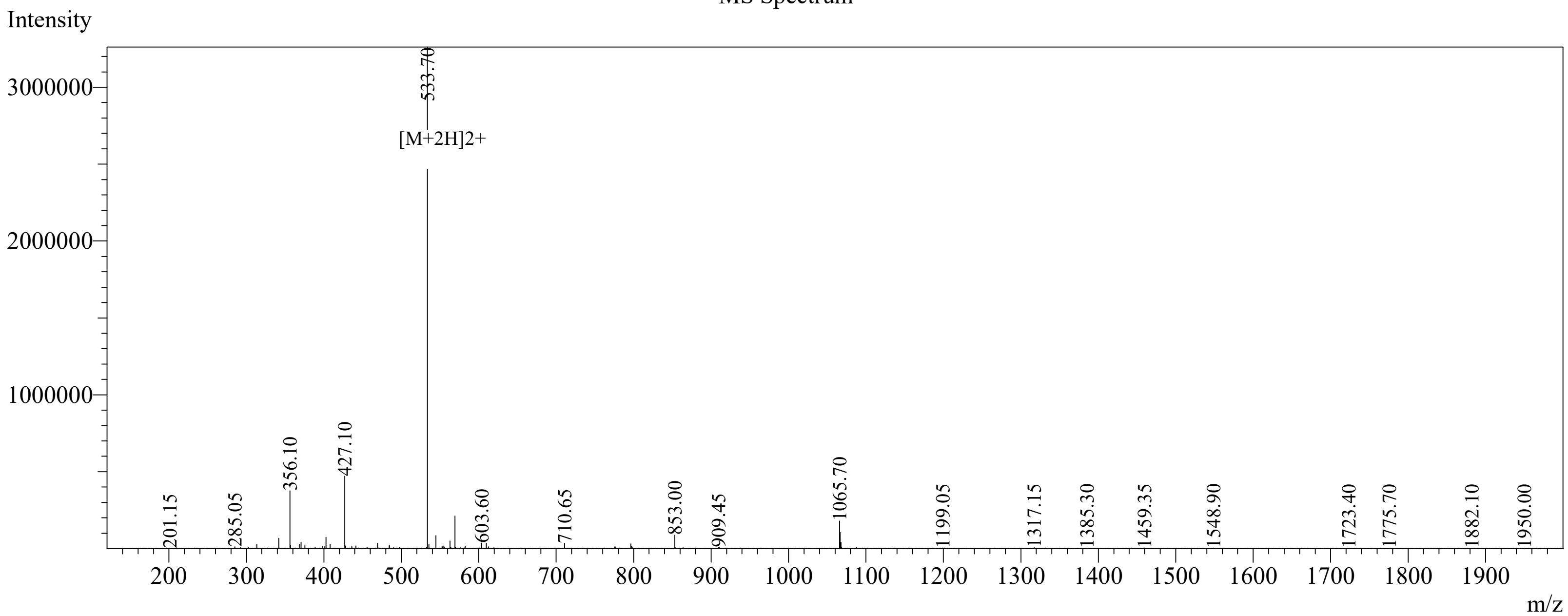

Sample Information
Dissolution method :0.1mg sample dissolved to 0.5mL by 100%H2O
Injection Volume :1ul

Name :P21421-1-RL-(biotin labeled)peptide
Sequence :RLPVK(Biotin)AR
Modification :K(Biotin)
Lot No. :P21421-1-RL-(biotin labeled)peptide
Theoretical :1065.345
Observed :1065.40

Interface :ESI
Nebulizing Gas Flow :1.50L/min
CDL Temp :250℃
CDL Volt :0v
Block Temp :200

Prerod Bias :+4.5kv
Detector :-0.2kv
T.Flow :0.2ml/min
B.conc :50%H2O/50%MeOH

生工生物工程（上海）股份有限公司

地址：上海市松江区香闵路698号
Add: 698 Xiang Min Road Songjiang Shanghai China
电话/Tel: 400-821-0268
传真/Fax: 86-21-37772170
邮箱/Email: Sales@sangon.com
网址/Web: wwww.sangon.com

MS Spectrum

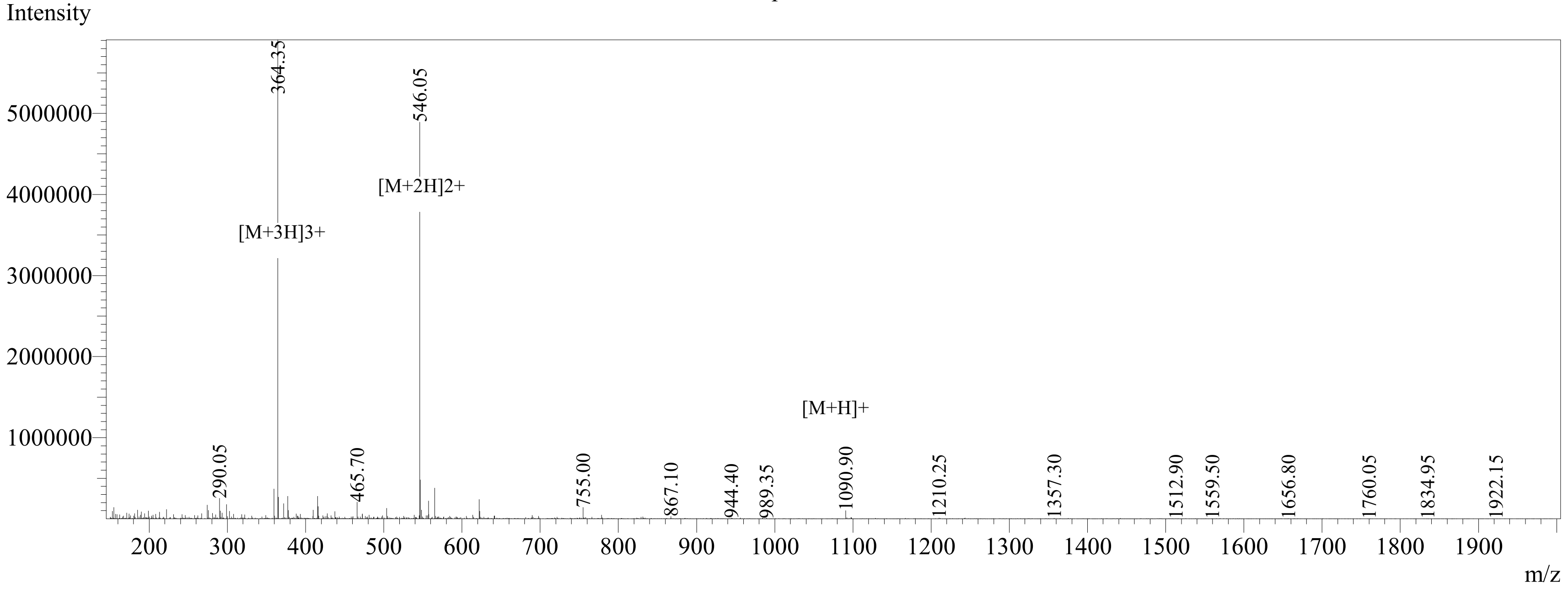

Sample Information
Dissolution method :0.1mg sample dissolved to 0.5mL by 50%ACN and 50%H2O

Injection Volume :1ul

Name :P27704-3 -RL-(Dabcyl labeled) peptide
Sequence :RLPVK（Dabcyl）AR
Modification :K（Dabcyl）
Lot No. :P27704-3-RL-(Dabcyl labeled) peptide
Theoretical :1090.335
Observed :1090.05

Interface :ESI
Nebulizing Gas Flow :1.50L/min
CDL Temp :250℃
CDL Volt :0v
Block Temp :200

Prerod Bias :+4.5kv
Detector :-0.2kv
T.Flow :0.2ml/min
B.conc :50%H2O/50%MeOH

生工生物工程（上海）股份有限公司

地址：上海市松江区香闵路698号
电话/Tel: 400-821-0268
邮箱/Email: Sales@sangon.com

Add: 698 Xiang Min Road Songjiang Shanghai China
传真/Fax: 86-21-37772170
网址/Web: wwww.sangon.com
