# Supplementary material for: Design of a recombinant asparaginyl ligase for site-specific modification using efficient recognition and nucleophile motifs
Source: Commun Chem. 2024 Apr 18;7:87. doi: 10.1038/s42004-024-01173-8 (PMC11026461; doi:10.1038/s42004-024-01173-8)
Supplement: Supplementary file 6 — Reporting Summary [file 42004_2024_1173_MOESM6_ESM.pdf]

nature portfolio

Corresponding author(s): Tingdong Li & Shengxiang Ge

Last updated by author(s): Mar 26, 2024

Reporting Summary

Nature Portfolio wishes to improve the reproducibility of the work that we publish. This form provides structure for consistency and transparency in reporting. For further information on Nature Portfolio policies, see our Editorial Policies and the Editorial Policy Checklist.

Statistics

For all statistical analyses, confirm that the following items are present in the figure legend, table legend, main text, or Methods section.

n/a	Confirmed	
☐	☒	The exact sample size (*n*) for each experimental group/condition, given as a discrete number and unit of measurement
☐	☒	A statement on whether measurements were taken from distinct samples or whether the same sample was measured repeatedly
☐	☒	The statistical test(s) used AND whether they are one- or two-sided<br>*Only common tests should be described solely by name; describe more complex techniques in the Methods section.*
☒	☐	A description of all covariates tested
☒	☐	A description of any assumptions or corrections, such as tests of normality and adjustment for multiple comparisons
☐	☒	A full description of the statistical parameters including central tendency (e.g. means) or other basic estimates (e.g. regression coefficient) AND variation (e.g. standard deviation) or associated estimates of uncertainty (e.g. confidence intervals)
☐	☒	For null hypothesis testing, the test statistic (e.g. *F*, *t*, *r*) with confidence intervals, effect sizes, degrees of freedom and *P* value noted<br>*Give P values as exact values whenever suitable.*
☒	☐	For Bayesian analysis, information on the choice of priors and Markov chain Monte Carlo settings
☒	☐	For hierarchical and complex designs, identification of the appropriate level for tests and full reporting of outcomes
☒	☐	Estimates of effect sizes (e.g. Cohen's *d*, Pearson's *r*), indicating how they were calculated

Our web collection on statistics for biologists contains articles on many of the points above.

Software and code

Policy information about availability of computer code

Data collection	1. The gene encoding OaAEP1 was codon-optimized using the Codon Adaptation Tool (http://www.jcat.de/); 2. Protein concentrations were quantified by measuring the absorbance at 280 nm on Multiskan® GO spectrophotometer; 3. The optical density at 450 nm with reference wavelength at 630nm of enzyme-linked immunosorbent assays was determined by a microplate spectrophotometer (Autobio, Zhengzhou, China); 4. For kinetics assay, the fluorescence was measured using a Gentier 96E real-time PCR system (Tianlong, Xi'an, China); 5. The mass spectrometry raw data was performed on an Thermo Fisher Orbitrap Eclipse Tribrid mass spectrometer.
Data analysis	1. Sequence coverage view of OaAEP1-C247A-aa55-351 protein by LC-MS/MS analysis was performed on an Thermo Fisher Orbitrap Eclipse Tribrid mass spectrometer; 2. Statistical analyses were performed using GraphPad Prism version 9.0.

For manuscripts utilizing custom algorithms or software that are central to the research but not yet described in published literature, software must be made available to editors and reviewers. We strongly encourage code deposition in a community repository (e.g. GitHub). See the Nature Portfolio guidelines for submitting code & software for further information.

Data

Policy information about availability of data

All manuscripts must include a data availability statement. This statement should provide the following information, where applicable:

- Accession codes, unique identifiers, or web links for publicly available datasets
- A description of any restrictions on data availability
- For clinical datasets or third party data, please ensure that the statement adheres to our policy

All data supporting the findings of this research are available within the article and its corresponding supplementary information file. The HPLC chromatograms and LC/MS spectra of the peptides used in this manuscript are available in Supplementary Data 1. All other data or sources are available from the corresponding author on reasonable request.

Research involving human participants, their data, or biological material

Policy information about studies with human participants or human data. See also policy information about sex, gender (identity/presentation), and sexual orientation and race, ethnicity and racism.

Reporting on sex and gender	Not involved in this study.
Reporting on race, ethnicity, or other socially relevant groupings	Not involved in this study.
Population characteristics	Not involved in this study.
Recruitment	Not involved in this study.
Ethics oversight	Not involved in this study.

Note that full information on the approval of the study protocol must also be provided in the manuscript.

Field-specific reporting

Please select the one below that is the best fit for your research. If you are not sure, read the appropriate sections before making your selection.

☒ Life sciences ☐ Behavioural & social sciences ☐ Ecological, evolutionary & environmental sciences

For a reference copy of the document with all sections, see nature.com/documents/nr-reporting-summary-flat.pdf

Life sciences study design

All studies must disclose on these points even when the disclosure is negative.

Sample size	All the enzyme-linked immunosorbent assays were performed triplicates.
Data exclusions	No data excluded.
Replication	All experiments were performed twice at least or by different instruments with consistent results.
Randomization	The assays performed in this study require a reasonable approach, so randomization was not acceptable in our experiment set up.
Blinding	Blinding was not relevant to this study.

Reporting for specific materials, systems and methods

We require information from authors about some types of materials, experimental systems and methods used in many studies. Here, indicate whether each material, system or method listed is relevant to your study. If you are not sure if a list item applies to your research, read the appropriate section before selecting a response.

Materials & experimental systems

n/a	Involved in the study
☐	☒ Antibodies
☒	☐ Eukaryotic cell lines
☒	☐ Palaeontology and archaeology
☒	☐ Animals and other organisms
☒	☐ Clinical data
☒	☐ Dual use research of concern
☒	☐ Plants

Methods

n/a	Involved in the study
☒	☐ ChIP-seq
☒	☐ Flow cytometry
☒	☐ MRI-based neuroimaging

Antibodies

Antibodies used

The antibody 5D6 against Pep133 and 17H11 against SARS-CoV-2 nucleocapsid protein was prepared in-house using mouse hybridoma technology, in which the Balb/c mice were immunized with keyhole limpet hemocyanin-coupled Pep133 or SARS-CoV-2 nucleocapsid protein.

Validation

The aboved antibodies have been verified by positive controls.

Plants

Seed stocks

Not involved in this study.

Novel plant genotypes

Not involved in this study.

Authentication

Not involved in this study.
